# Association of Insulin Resistance and Type 2 Diabetes With Gut Microbial Diversity: A Microbiome-Wide Analysis From Population Studies

**DOI:** 10.1001/jamanetworkopen.2021.18811

**Published:** 2021-07-29

**Authors:** Zhangling Chen, Djawad Radjabzadeh, Lianmin Chen, Alexander Kurilshikov, Maryam Kavousi, Fariba Ahmadizar, M. Arfan Ikram, Andre G. Uitterlinden, Alexandra Zhernakova, Jingyuan Fu, Robert Kraaij, Trudy Voortman

**Affiliations:** 1 Department of Epidemiology, Erasmus MC, University Medical Center, Rotterdam, the Netherlands; 2 Department of Nutrition, Harvard T.H. Chan School of Public Health, Boston, Massachusetts; 3 Department of Internal Medicine, Erasmus MC, University Medical Center, Rotterdam, the Netherlands; 4 Department of Genetics, University of Groningen, University Medical Center Groningen, Groningen, the Netherlands; 5 Department of Pediatrics, University of Groningen, University Medical Center Groningen, Groningen, the Netherlands; 6 Department of Cardiology, Nanjing Medical University, The First Affiliated Hospital of Nanjing Medical University, Nanjing.

## Abstract

**Importance:**

Previous studies have indicated that gut microbiome may be associated with development of type 2 diabetes. However, these studies are limited by small sample size and insufficient for confounding. Furthermore, which specific taxa play a role in the development of type 2 diabetes remains unclear.

**Objective:**

To examine associations of gut microbiome composition with insulin resistance and type 2 diabetes in a large population-based setting controlling for various sociodemographic and lifestyle factors.

**Design, Setting, and Participants:**

This cross-sectional analysis included 2166 participants from 2 Dutch population-based prospective cohorts: the Rotterdam Study and the LifeLines-DEEP study.

**Exposures:**

The 16S ribosomal RNA method was used to measure microbiome composition in stool samples collected between January 1, 2012, and December 31, 2013. The α diversity (Shannon, richness, and Inverse Simpson indexes), β diversity (Bray-Curtis dissimilarity matrix), and taxa (from domain to genus level) were identified to reflect gut microbiome composition.

**Main Outcomes and Measures:**

Associations among α diversity, β diversity, and taxa with the Homeostatic Model Assessment of Insulin Resistance (HOMA-IR) and with type 2 diabetes were examined. Glucose and insulin were measured to calculate the HOMA-IR. Type 2 diabetes cases were identified based on glucose levels and medical records from January 2012 to December 2013. Analyses were adjusted for technical covariates, lifestyle, sociodemographic, and medical factors. Data analysis was performed from January 1, 2018, to December 31, 2020.

**Results:**

There were 2166 participants in this study: 1418 from the Rotterdam Study (mean [SD] age, 62.4 [5.9] years; 815 [57.5%] male) and 748 from the LifeLines-DEEP study (mean [SD] age, 44.7 [13.4] years; 431 [57.6%] male); from this total, 193 type 2 diabetes cases were identified. Lower microbiome Shannon index and richness were associated with higher HOMA-IR (eg, Shannon index, −0.06; 95% CI, −0.10 to −0.02), and patients with type 2 diabetes had a lower richness than participants without diabetes (odds ratio [OR], 0.93; 95% CI, 0.88-0.99). The β diversity (Bray-Curtis dissimilarity matrix) was associated with insulin resistance (*R*^2^ = 0.004, *P* = .001 in the Rotterdam Study and *R*^2^ = 0.005, *P* = .002 in the LifeLines-DEEP study). A total of 12 groups of bacteria were associated with HOMA-IR or type 2 diabetes. Specifically, a higher abundance of Christensenellaceae (β = −0.08; 95% CI, −0.12 to −0.03: *P* < .001), Christensenellaceae R7 group (β = −0.07; 95% CI, −0.12 to −0.03; *P* < .001), *Marvinbryantia* (β = −0.07; 95% CI, −0.11 to −0.03; *P* < .001), Ruminococcaceae UCG005 (β = −0.09; 95% CI, −0.13 to −0.05; *P* < .001), Ruminococcaceae UCG008 (β = −0.07; 95% CI, −0.11 to −0.03; *P* < .001), Ruminococcaceae UCG010 (β = −0.08; 95% CI, −0.12 to −0.04; *P* < .001), or Ruminococcaceae NK4A214 group (β = −0.09; 95% CI, −0.13 to −0.05; *P* < .001) was associated with lower HOMA-IR. A higher abundance of Clostridiaceae 1 (OR, 0.51; 95% CI, 0.41-0.65; *P* < .001), Peptostreptococcaceae (OR, 0.56; 95% CI, 0.45-0.70; *P* < .001), *C sensu stricto* 1 (OR, 0.51; 95% CI, 0.40-0.65; *P* < .001), *Intestinibacter* (OR, 0.60; 95% CI, 0.48-0.76; *P* < .001), or *Romboutsia* (OR, 0.55; 95% CI, 0.44-0.70; *P* < .001) was associated with less type 2 diabetes. These bacteria are all known to produce butyrate.

**Conclusions and Relevance:**

In this cross-sectional study, higher microbiome α diversity, along with more butyrate-producing gut bacteria, was associated with less type 2 diabetes and with lower insulin resistance among individuals without diabetes. These findings could help provide insight into the etiology, pathogenesis, and treatment of type 2 diabetes.

## Key Points

**Question:**

Which gut microbial taxa are associated with the development of type 2 diabetes?

**Findings:**

In this cross-sectional study of 2166 participants in 2 large population-based studies, lower microbiome Shannon index and richness were associated with less insulin resistance, and patients with type 2 diabetes (n = 193) had lower richness than participants without diabetes. Furthermore, 12 groups of butyrate-producing bacteria were significantly linked to insulin resistance or type 2 diabetes.

**Meaning:**

These findings suggest that higher gut microbial diversity, along with specifically more butyrate-producing bacteria, may play a role in the development of type 2 diabetes, which may help guide future prevention and treatment strategies.

## Teaser

This cross-sectional study assesses whether gut microbial taxa play a role in the development of type 2 diabetes using data from 2 Dutch population-based prospective cohorts.

## Introduction

Type 2 diabetes is a common complex metabolic disorder. Currently, more than 380 million people live with type 2 diabetes globally, and this number is expected to increase to more than 550 million by 2030.^1^ Recently, studies^2,3,4,5^ have indicated a role of gut microbiome in type 2 diabetes. Differences in gut microbiome composition with type 2 diabetes status may comprise pathways on how dietary and other environmental factors affect development of insulin resistance and type 2 diabetes. Several studies^2,3,4^ have indicated that, compared with healthy participants, patients with type 2 diabetes have a lower overall α diversity of gut microbiome composition. More specifically, lower abundance of certain butyrate-producing bacteria, such as class Clostridia and genus *Faecalibacterium*, have been observed in patients with type 2 diabetes.^2,3,4,5^ For example, Larsen et al^3^ reported a lower abundance of Clostridia in patients with type 2 diabetes, and Qin et al^5^ observed that patients with type 2 diabetes had lower abundance of *Faecalibacterium*. In addition, some nonbutyrate bacteria, such as *Haemophilus parainfluenzae*, have also been reported to be associated with type 2 diabetes.^5^ However, these associations were not very strong, and most of these specific bacteria have not been reproduced in other cohorts.^6^ Furthermore, these previous studies^2,3,4,5,6^ had several limitations. They were limited in sample sizes, ranging from 20^3^ to 784^2^ participants. Larger samples with more statistical power may be required to detect true associations. Moreover, in most of these previous observational studies,^2,3,4,5^ key confounders, such as lifestyle and socioeconomic status, that are known to be determinants of gut microbiome and type 2 diabetes^7^ were not controlled for. Finally, because almost all these previous studies^2,3,4,5^ were conducted in patient populations or case-control settings, it is unclear whether associations are also relevant on a population level and whether type 2 diabetes–associated bacteria are associated to key subclinical parameters, such as insulin resistance and fasting glucose, among groups without diabetes. Examining associations for gut microbiome and such subclinical parameters will help investigators infer potential pathways behind gut microbiome and type 2 diabetes risk. Acquiring and updating this knowledge are particularly relevant, considering the increasing recognition that gut microbiome may play an important role in the development of type 2 diabetes and could be a promising target for prevention and treatment of type 2 diabetes. Therefore, we aimed to investigate the associations between gut microbiome composition with insulin resistance and type 2 diabetes in 2 large population-based cohorts, using a microbiome-wide approach and taking into account various sociodemographic and lifestyle factors.

## Methods

### Study Design

The current study was embedded within 2 ongoing, population-based, prospective cohorts in the Netherlands: the Rotterdam Study (RS) and LifeLines-DEEP (LLD) study. The RS is a prospective cohort study of participants 45 years or older at baseline living in the Ommoord District of Rotterdam, the Netherlands. It consists of 3 subcohorts, and the details on its design are described elsewhere.^8^ The data from the RS population that were used in the current analyses were collected within the 2012-2014 visit of the third subcohort in which 3132 individuals participated. The RS was approved by the Medical Ethics Committee of Erasmus Medical Center and by the review board of The Netherlands Ministry of Health, Welfare, and Sports. All participants gave written informed consent. The LLD study is a prospective, population-based cohort study of 1539 participants 18 years or older living in the 3 provinces in the northern part of the Netherlands: Groningen, Friesland, and Drenthe. More details can be found elsewhere.^9^ The current analyses in the LLD study population were embedded within the baseline visit (in 2013). The LLD study was approved by the ethics committee of the University Medical Center Groningen. All participants provided written informed consent before enrollment. Data analysis was performed from January 1, 2018, to December 31, 2020. All data were deidentified. This current study followed the Strengthening the Reporting of Observational Studies in Epidemiology (STROBE) reporting guideline for cross-sectional studies.^10^

### Participants in the Current Analyses

In the 2 cohorts of the 4671 participants (3132 from the RS and 1539 from the LLD study), 2607 had data on gut microbiome composition (1427 from the RS and 1180 from the LLD study). From these 2607 participants, we excluded participants without information on type 2 diabetes, resulting in 2166 participants for analyses on gut microbiome and type 2 diabetes status (1418 from the RS and 748 from the LLD study). For analyses on insulin resistance, we additionally excluded participants with prevalent type 2 diabetes or without data on insulin resistance, resulting in 1984 participants (1253 for the RS and 731 for the LLD study) (eFigure 1 in the Supplement). eTable 1 in the Supplement compares the included and excluded participants.

### Collection of Gut Microbiome Data

Details on microbiome data collection in the RS^11,12^ and the LLD study^9,13^ are described elsewhere. Briefly, for the RS, participants were requested to collect a stool sample at their home using a Commode Specimen Collection System (Covidien) and feces collection tube (Minigrip Nederland) and to send the sample through regular mail to the Erasmus Medical Center. On arrival, samples were recorded and stored at −20 °C. The time each sample was in the mail was recorded^14^ and adjusted for in our analyses. An automated stool DNA isolation kit (Diasorin) was used to isolate bacterial DNA. In the RS sample, a confounding effect driven by DNA isolation batches was observed and therefore adjusted for in analyses. The V3 and V4 hypervariable regions of the bacterial 16S ribosomal RNA gene were amplified and sequenced on the IIIumina MiSeq platform (Illumina Inc). For the LLD study, the stool samples were retrieved from participants’ homes by students of University Medical Center Groningen. DNA was isolated with the AllPrep DNA/RNA Mini Kit (Qiagen). The V4 hypervariable region of the bacterial 16S ribosomal RNA gene were amplified and sequenced on the Illumina MiSeq platform. To decrease domain-dependent bias associated with different hypervariable regions between the 2 cohorts, a direct classification of 16S sequencing reads using a naive bayesian classifier from the Ribosomal Database Project and SILVA 16S database release 128 was used to reconstruct taxonomic composition of studied communities, with binning posterior probability cutoff of 0.8.^14^ The eMethods in the Supplement give more details for collecting gut microbiome data. As a result, in the RS, the microbiome data contained information on 2 domains, 8 phyla, 15 classes, 18 orders, 33 families, and 126 genera. In the LLD study, the microbiome data contained 2 domain, 12 phyla, 21 classes, 27 orders, 48 families, and 184 genera (eFigure 2 in the Supplement). We also calculated α diversity (Shannon, richness, and Inverse Simpson indexes), and β diversity (Bray-Curtis dissimilarity matrix) at the genus level using the R package vegan (R Foundation for Statistical Computing) in both cohorts separately.

### Assessment of Insulin Resistance and Type 2 Diabetes

Insulin resistance and type 2 diabetes were the primary outcomes of the current analysis. In both cohorts, fasting blood samples were collected from 2012 through 2013. In the RS, glucose levels were examined with the glucose hexokinase method. Serum insulin was measured by electrochemiluminescence immunoassay technology. In the LLD study, glucose levels were measured by hydrogen 1 nuclear magnetic resonance, and serum insulin was measured on an architect system (Abbott Laboratories). Insulin resistance in the 2 cohorts was calculated using the Homeostatic Model Assessment of Insulin Resistance (HOMA-IR) as follows: fasting insulin × fasting glucose/22.5.^15^

Data on type 2 diabetes status in both cohorts were collected in 2013. In the RS, information was collected from general practitioners’ records, pharmacies’ databases, structured home interview, hospital discharge letters, and glucose levels measured in the research center. In the LLD study, information on type 2 diabetes was collected through self-reported questionnaires and fasting glucose measured in the research center.^9^ Cases of type 2 diabetes were identified according to World Health Organization criteria^16^: a fasting blood glucose concentration of 126 mg/dL or higher, nonfasting blood glucose of 200 mg/dL (when fasting samples were unavailable) (to convert glucose to millimoles per liter, multiply by 0.0555), and/or the use of blood glucose-lowering drugs (insulin or oral hypoglycemic agent) or prescribed dietary treatment and registration of the diagnosis diabetes. All potential events of type 2 diabetes were independently adjudicated by 2 study physicians. In case of disagreement, consensus was sought from an endocrinologist.^17^

### Assessment of Covariates

In both cohorts, information on educational level, smoking status, dietary intake, and physical activity was assessed through interviews and questionnaires. Furthermore, information on medication use was obtained from general practitioners, pharmacies’ databases, the nationwide medical registry, or follow-up examinations.^18,19^ Details on the collection of data on these covariates aref provided in the eMethods in the Supplement.

### Data Analyses

We examined associations between diversity measures representing wide gut microbiome composition with insulin resistance (HOMA-IR, continuous variable) and type 2 diabetes (yes/no) and of specific taxa with insulin resistance (HOMA-IR) and type 2 diabetes. We used natural log-transformed HOMA-IR for all analyses to obtain a normal distribution. For diversity measures, we investigated associations for Shannon index, richness, and Inverse Simpson index and insulin resistance using linear regression and type 2 diabetes using logistic regression. We analyzed associations of Bray-Curtis dissimilarity matrix with insulin resistance and type 2 diabetes using permutation analysis of variance (1000 permutations). For analyses for gut microbial taxa, we first added 1 to all taxa counts to prevent missingness derived from log zero. Subsequently, to reduce the skewness of the distribution of microbial taxa counts, we performed natural log transformation. The associations between microbial taxa and insulin resistance and type 2 diabetes were assessed by linear regression and logistic regression, respectively.

For all analyses, we adjusted for age, sex, time in mail (RS only), and DNA batch effect (RS only) in model 1. In model 2, we additionally adjusted for alcohol intake, energy intake, smoking, educational level (RS only), and physical activity. In model 3, we additionally adjusted for body mass index (BMI; calculated as weight in kilograms divided by height in meters squared). Last, in model 4, we additionally adjusted for use of lipid-lowering drugs and proton pump inhibitors.

### Sensitivity Analyses

We conducted 3 sets of sensitivity analyses based on model 4. First, we examined interaction effects of α diversity or taxa with age, sex, or BMI by including these interaction terms (eg, Shannon index × sex) 1 at a time into model 4. In case of statically significant interaction terms, stratified analyses by these factors would be conducted (eg, separate for men and women). Second, we reexamined associations of α diversity, β diversity, and taxa with HOMA-IR and type 2 diabetes by additionally adjusting for diet quality score and blood pressure in the RS. Third, because use of blood glucose–lowering drugs, such as metformin, was a criterion of type 2 diabetes diagnosis in our study, metformin itself may be also associated with gut microbiome composition^20^; therefore, we performed a sensitivity analysis in the RS in which we reexamined associations between α diversity and type 2 diabetes after excluding patients who used metformin (n = 1337 participants, 95 cases) and after excluding patients who did not use metformin (n = 1323 participants, 81 cases). We did not examine associations between taxa and type 2 diabetes after excluding these patients because excluding these participants resulted in smaller groups and fewer cases, which combined with the multiple tests of taxa would result in extremely low statistical power.

Because some data (eg, data on physical activity and energy intake) were collected using different questionnaires between 2 cohorts, to better achieve control for confounding for all main analyses, we first conducted analyses in the 2 studies separately and then combined the associations for α diversity and for taxa available in both cohorts using fixed-effects meta-analysis. Associations of β diversity could not be pooled and were presented for each cohort separately.

### Statistical Analysis

Although we used 3 indexes in the analysis of α diversity and 1 for β diversity, cutoff values for statistical significance were set at *P* < .05 for analyses of α and β diversity because a single hypothesis was tested. For analyses of taxa, *P* < .0005 was set for analyses of taxa, taking into account that we performed 103 independent tests (0.05/103) among microbial taxa as calculated based on the method of Li and Ji.^21^ All analyses were conducted in R, version 3.1.2 (R Foundation for Statistical Computing).

## Results

### Baseline Characteristics

A total of 193 type 2 diabetes cases were identified among 2166 participants, 1418 from the Rotterdam Study (mean [SD] age, 62.4 [5.9] years; 815 [57.5%] male; 176 [12.4%] with type 2 diabetes) and 748 from the LLD study (mean [SD] age, 44.7 [13.4] years; 431 [57.6%] male; 17 [2.3%] with type 2 diabetes). Characteristics of the study population are given in Table 1.

**Table 1.** Characteristics of Participants

Characteristic	Finding^a^ (N = 2166)	
	Rotterdam Study (n = 1418)	LifeLines-DEEP study (n = 748)
Age, mean (SD), y	62.4 (5.9)	44.7 (13.4)
Sex		
Female	815 (57.5)	431 (57.6)
Male	603 (42.5)	317 (42.4)
Smoking		
Current	193 (13.6)	155 (20.7)
Nonsmoker		593 (79.3)
Ever, quit	706 (49.8)	
Never	519 (36.6)	
Educational level		
Primary	108 (7.6)	NA
Lower	474 (33.4)	
Intermediate	397 (28.0)	
Higher	435 (30.7)	
Physical activity, median (IQR)^b^	42.9 (17.7-82.8)	55.5 (25.8-57.8)
BMI, mean (SD)	27.5 (4.5)	25.2 (4.1)
Alcohol intake, median (IQR), g/d	8.1 (1.4-19.7)	2.2 (0.7-11.1)
Energy intake, median (IQR), kcal/d	2243.2 (1869.4-2733.3)	1862.0 (1526.1-2282.8)
Lipid-lowering medication use^c^	393 (27.7)	32 (4.3)
Proton pump inhibitor use	257 (18.1)	63 (8.4)

Abbreviations: BMI, body mass index (calculated as weight in kilograms divided by height in meters squared); IQR, interquartile range; NA, not available.

^a^ Data are presented as number (percentage) of participants unless otherwise indicated.

^b^ Physical activity level is expressed as metabolic equivalent of task-hours per week in the Rotterdam Study, and is calculated as a continuous score with a theoretical range of 1-100 in the LifeLines-DEEP study.

^c^ Lipid-lowering medication is defined as statin use in the LifeLines-DEEP study.

### Associations Between Gut Microbial α Diversity With Insulin Resistance and Type 2 Diabetes

Associations were similar across all 4 models (Table 2). For the main model (model 4), higher Shannon index and richness were associated with lower HOMA-IR (Shannon index, −0.06; 95% CI, −0.10 to −0.02; *P* = .02; richness, −0.07; 95% CI, −0.11 to −0.03; *P* = .03). For type 2 diabetes, higher richness was associated with a lower prevalence of type 2 diabetes (odds ratio [OR], 0.93; 95% CI, 0.88-0.99; *P* = .04) (Table 3). A higher Shannon index was not associated with a lower prevalence of type 2 diabetes (OR, 0.83; 95% CI, 0.66-1.03; *P* = .06). Inverse Simpson index was not associated with HOMA-IR (β = −0.04; 95% CI, −0.08 to 0.002) or type 2 diabetes (OR, 0.91; 95% CI, 0.73-1.14: *P* = .25) (Table 2 and Table 3).

**Table 2.** Association of α Diversity and Insulin Resistance

Model^a^	β (95% CI)			*P* value^c^
	Rotterdam Study (n = 1253)	LifeLines-DEEP study (n = 731)	Pooled results (n = 1984)^b^	
**Shannon index**				
Model 1	−0.16 (−0.25 to −0.07)	−0.09 (−0.16 to −0.02)	−0.11 (−0.15 to −0.07)	.01
Model 2	−0.08 (−0.14 to −0.03)	−0.06 (−0.12 to 0.0004)	−0.07 (−0.11 to −0.03)	.01
Model 3	−0.08 (−0.14 to −0.03)	−0.06 (−0.12 to −0.03)	−0.07 (−0.11 to −0.03)	.01
Model 4	−0.07 (−0.12 to −0.01)	−0.05 (−0.11 to 0.004)	−0.06 (−0.10 to −0.02)	.02
**Richness**				
Model 1	−0.12 (−0.18 to −0.07)	−0.12 (−0.19 to −0.05)	−0.12 (−0.16 to −0.08)	.01
Model 2	−0.08 (−0.14 to −0.02)	−0.06 (−0.12 to −0.01)	−0.07 (−0.11 to −0.03)	.03
Model 3	−0.08 (−0.14 to −0.02)	−0.06 (−0.12 to −0.01)	−0.07 (−0.11 to −0.03)	.03
Model 4	−0.07 (−0.12 to −0.02)	−0.06 (−0.12 to −0.001)	−0.07 (−0.11 to −0.03)	.03
**Inverse Simpson index**				
Model 1	−0.22 (−0.40 to −0.06)	−0.30 (−0.78 to 0.18)	−0.23 (−0.40 to −0.06)	.03
Model 2	−0.06 (−0.11 to −0.004)	−0.03 (−0.09 to 0.03)	−0.05 (−0.09 to −0.01)	.04
Model 3	−0.06 (−0.11 to −0.004)	−0.03 (−0.09 to 0.03)	−0.05 (−0.09 to −0.01)	.04
Model 4	−0.04 (−0.10 to 0.01)	−0.03 (−0.09 to 0.03)	−0.04 (−0.08 to 0.002)	.05

^a^ Model 1: age, sex, time in mail (the Rotterdam Study), and batch (the Rotterdam Study). Model 2: model 1 plus smoking, educational level (the Rotterdam Study), physical activity, alcohol intake, and total energy intake. Model 3: model 2 plus body mass index. Model 4: model 3 plus lipid-lowering medication and proton pump inhibitor use.

^b^ Pooled results are calculated based on an inverse variance–weighted, mixed-effect meta-analysis. No significant heterogeneity was observed across cohorts.

^c^ *P* < .05 was considered statistically significant.

**Table 3.** Association of α Diversity and Type 2 Diabetes

Model^a^	Odds ratio (95% CI)			*P* value^c^
	Rotterdam Study (n = 1418)	LifeLines-DEEP study (n = 748)	Pooled results (n = 2166)^b^	
**Shannon index**				
Model 1	0.80 (0.67-0.96)	0.74 (0.46-1.20)	0.79 (0.67-0.94)	.03
Model 2	0.71 (0.57-0.89)	0.85 (0.53-1.38)	0.73 (0.60-0.90)	.03
Model 3	0.76 (0.60-0.96)	0.84 (0.51-1.38)	0.78 (0.63-0.96)	.03
Model 4	0.80 (0.63-1.02)	0.94 (0.55-1.62)	0.83 (0.66-1.03)	.06
**Richness**				
Model 1	0.74 (0.64-0.91)	0.78 (0.48-1.28)	0.76 (0.64-0.90)	.04
Model 2	0.73 (0.58-0.90)	0.83 (0.50-1.36)	0.74 (0.61-0.90)	.04
Model 3	0.78 (0.62-0.98)	0.86 (0.52-1.45)	0.79 (0.64-0.98)	.04
Model 4	0.80 (0.63-1.02)	0.95 (0.87-1.00)	0.93 (0.88-0.99)	.04
**Inverse Simpson index**				
Model 1	0.88 (0.73-1.05)	0.91 (0.57-1.45)	0.88 (0.74-1.04)	.10
Model 2	0.79 (0.64-0.99)	1.05 (0.66-1.66)	0.84 (0.68-1.02)	.08
Model 3	0.84 (0.66-1.06)	1.00 (0.63-1.62)	0.87 (0.70-1.07)	.23
Model 4	0.88 (0.69-1.13)	1.08 (0.64-1.82)	0.91 (0.73-1.14)	.25

^a^ Model 1: age, sex, time in mail (the Rotterdam Study), and batch (the Rotterdam Study). Model 2: model 1 plus smoking, educational level (the Rotterdam Study), physical activity, alcohol intake, and total energy intake. Model 3: model 2 plus body mass index. Model 4: model 3 plus lipid-lowering medication use and proton pump inhibitor use.

^b^ Pooled results are calculated based on an inverse variance–weighted, mixed-effect meta-analysis. No significant heterogeneity was observed across cohorts.

^c^ *P* < .05 was considered statistically significant.

### Associations Between Gut Microbial β Diversity With Insulin Resistance and Type 2 Diabetes

The β diversity (Bray-Curtis dissimilarity matrix) was associated with insulin resistance (*R*^2^ = 0.004, *P* = .001 in the RS and *R*^2^ = 0.005, *P* = .002 in the LLD study). Furthermore, the Bray-Curtis dissimilarity matrix was significantly different between individuals with and without type 2 diabetes in the RS, although not in the LLD study (*R*^2^ = 0.003, *P* = .001 in the RS and *R*^2^ = 0.001, *P* = .65 in the LLD study).

### Associations Between Gut Microbial Taxa With Insulin Resistance and Type 2 Diabetes

After multiple adjustment (model 4), we observed 7 taxa to be associated with HOMA-IR and 5 taxa with type 2 diabetes in the meta-analysis. Specifically, a higher abundance of Christensenellaceae (β = −0.08; 95% CI, −0.12 to −0.03: *P* < .001), Christensenellaceae R7 group (β = −0.07; 95% CI, −0.12 to −0.03; *P* < .001), *Marvinbryantia* (β = −0.07; 95% CI, −0.11 to −0.03; *P* < .001), Ruminococcaceae UCG005 (β = −0.09; 95% CI, −0.13 to −0.05; *P* < .001), Ruminococcaceae UCG008 (β = −0.07; 95% CI, −0.11 to −0.03; *P* < .001), Ruminococcaceae UCG010 (β = −0.08; 95% CI, −0.12 to −0.04; *P* < .001), or Ruminococcaceae NK4A214 group (β = −0.09; 95% CI, −0.13 to −0.05; *P* < .001) was associated with lower HOMA-IR (Table 4). A higher abundance of Clostridiaceae 1 (OR, 0.51; 95% CI, 0.41-0.65; *P* < .001), Peptostreptococcaceae (OR, 0.56; 95% CI, 0.45-0.70; *P* < .001), *Clostridium sensu stricto* 1 (OR, 0.51; 95% CI, 0.40-0.65; *P* < .001), *Intestinibacter* (OR, 0.60; 95% CI, 0.48-0.76; *P* < .001), or *Romboutsia* (OR, 0.55; 95% CI, 0.44-0.70; *P* < .001) was associated with less type 2 diabetes (Table 5). eTables 2 and 3 in the Supplement give all results in models 1 to 4 in the separated analyses of the 2 cohorts (including overlapping and nonoverlapping taxa) and the meta-analysis (overlapping taxa).

**Table 4.** Statistically Significant Pooled Associations Between Taxa and Insulin Resistance^a^

Taxon	β (95% CI)			*P* value^c^
	Rotterdam Study (n = 1253)	LifeLines-DEEP study (n = 731)	Pooled results (n = 1984)^b^	
Christensenellaceae	−0.09 (−0.14 to −0.03)	−0.06 (−0.12 to −0.002)	−0.08 (−0.12 to −0.03)	<.001
Christensenellaceae R7 group	−0.09 (−0.15 to −0.03)	−0.06 (−0.12 to 0.002)	−0.07 (−0.12 to −0.03)	<.001
*Marvinbryantia*	−0.07 (−0.13 to −0.02)	−0.08 (−0.13 to −0.02)	−0.07 (−0.11 to −0.03)	<.001
Ruminococcaceae UCG005	−0.11 (−0.16 to −0.05)	−0.07 (−0.12 to −0.01)	−0.09 (−0.13 to −0.05)	<.001
Ruminococcaceae UCG008	−0.10 (−0.14 to −0.04)	−0.04 (−0.09 to 0.02)	−0.07 (−0.11 to −0.03)	<.001
Ruminococcaceae UCG010	−0.10 (−0.16 to −0.05)	−0.06 (−0.12 to 0.0001)	−0.08 (−0.12 to −0.04)	<.001
Ruminococcaceae NK4A214 group	−0.09 (−0.15 to −0.04)	−0.08 (−0.14 to −0.03)	−0.09 (−0.13 to −0.05)	<.001

^a^ The current meta-analysis combined associations for 1 domain, 7 phyla, 14 classes, 16 orders, 30 families, and 112 genera. This table gives only significant pooled associations. For these significant pooled associations, no significant heterogeneity was observed across cohorts. eTable 2 in the Supplement gives the results for all overlapping taxa and insulin resistance in separated analyses and meta-analysis of the 2 studies. Model (corresponding model 4) is adjusted for age, sex, time in mail (the Rotterdam Study), batch (the Rotterdam Study), alcohol intake, total energy intake, smoking status, physical activity, body mass index, proton pump inhibitor use, lipid-lowering medication use, and educational level (the Rotterdam Study).

^b^ Pooled results are calculated based on an inverse variance–weighted, mixed-effect meta-analysis.

^c^ *P* < .001 was considered to be statistically significant.

**Table 5.** Statistically Significant Pooled Associations Between Taxa and Type 2 Diabetes^a^

Taxon	β (95% CI)			*P* value^c^
	Rotterdam Study (n = 1418)	LifeLines-DEEP study (n = 748)	Pooled results (n = 2166)^b^	
Clostridiaceae 1	0.42 (0.32-0.54)	1.07 (0.65-1.77)	0.51 (0.41-0.65)	<.001
Peptostreptococcaceae	0.52 (0.41-0.66)	0.89 (0.50-1.59)	0.56 (0.45-0.70)	<.001
*Clostridium sensu stricto* 1	0.42 (0.32-0.54)	1.08 (0.64-1.81)	0.51 (0.40-0.65)	<.001
*Intestinibacter*	0.50 (0.38-0.65)	1.06 (0.67-1.65)	0.60 (0.48-0.76)	<.001
*Romboutsia*	0.56 (0.44-0.71)	0.44 (0.13-1.45)	0.55 (0.44-0.70)	<.001

^a^ The current meta-analysis combined associations for 1 domain, 7 phyla, 14 classes, 16 orders, 30 families, and 112 genera. This table gives only significant pooled associations. For these significant pooled associations, no significant heterogeneity was observed across cohorts. eTable 2 in the Supplement gives the results for all overlapping taxa and insulin resistance in separated analyses and meta-analysis of the 2 studies. Model (corresponding model 4) is adjusted for age, sex, time in mail (the Rotterdam Study), batch (the Rotterdam Study), alcohol intake, total energy intake, smoking status, physical activity, body mass index, proton pump inhibitor use, lipid-lowering medication use, and educational level (the Rotterdam Study).

^b^ Pooled results are calculated based on an inverse variance–weighted, mixed-effect meta-analysis.

^c^ *P* < .001 was considered to be statistically significant.

### Additional Analyses

First, we observed that associations for α diversity or taxa with HOMA-IR or type 2 diabetes did not differ by age, sex, or BMI. Second, we observed similar results after additionally adjusting for diet quality score and blood pressure (eg, for HOMA-IR, β = −0.08; 95% CI, −0.12 to −0.03: *P* < .001 for *Clostridiales vadin* BB60 group) (eTables 4-6 in the Supplement). We also observed that the effect estimates for the associations between α diversity and type 2 diabetes were similar when excluding patients using metformin (OR, 0.84; 95% CI, 0.45-1.12 for Shannon index; OR, 0.99; 95% CI, 0.98-1.03 for richness; and OR, 1.01; 95% CI. 0.99-1.03 for Inverse Simpson index) or excluding patients without use of metformin (OR, 0.73; 95% CI, 0.46-1.17 for Shannon index; OR, 1.00; 95% CI, 0.99-1.02 for richness; and OR, 1.02; 95% CI, 0.98-1.05 for Inverse Simpson index), although none were statistically significant, likely because of the strongly reduced statistical power.

## Discussion

This cross-sectional study of a large, population-based sample found associations between gut microbiome composition and type 2 diabetes prevalence and with insulin resistance among individuals without diabetes, independent of several sociodemographic and lifestyle factors. Specifically, the study found that higher α diversity was associated with lower insulin resistance and lower prevalence of type 2 diabetes and that variations of gut microbial β diversity were associated with insulin resistance. The study also found that a higher abundance of these 12 taxa may benefit risk of insulin resistance and type 2 diabetes: Christensenellaceae, Clostridiaceae 1, Peptostreptococcaceae, Christensenellaceae R7 group, *Marvinbryantia*, Ruminococcaceae UCG005, Ruminococcaceae UCG008, Ruminococcaceae UCG010, Ruminococcaceae NK4A214 group, *C sensu stricto* 1, *Intestinibacter*, and *Romboutsia*.

Several previous studies^2,3,5^ have examined associations with type 2 diabetes but were limited by small sample size, restriction to patient settings, and the lack of adjustment for important confounders, such as energy intake, physical activity, and socioeconomic factors. The current study is the first, to our knowledge, to comprehensively investigate the associations between gut microbiome composition with type 2 diabetes in a large population-based sample for which we adjusted for a series of key confounders.

Similar to a previous study,^5^ the current study found that higher α diversity was associated with lower prevalence of type 2 diabetes. These associations were independent of energy intake, physical activity, educational level, smoking, and medication use. Furthermore, this evidence was extended to indicate that α and β diversity are linked to insulin resistance, further confirming that variation of gut microbiome composition is also closely associated with earlier stages in the development of type 2 diabetes.

Furthermore, 12 taxa were associated with insulin resistance or type 2 diabetes. All 12 are known to be butyrate-producing bacteria.^22,23,24^ These findings were in general similar to the inverse associations between several butyrate-producing species with insulin resistance observed by Pedersen et al^25^ among 277 Danish individuals without diabetes. For instance, *Clostridium* species and *Clostridiales* species were inversely associated with insulin resistance. The current findings were also in line with previous studies^5,6^ that reported that a higher abundance of the 2 butyrate-producing bacteria, Clostridiaceae 1 and *C sensu stricto *1, were associated with lower prevalence of type 2 diabetes. However, the current study yielded 10 novel associations. These 10, also all butyrate-producing bacteria, were all inversely associated with insulin resistance or type 2 diabetes: Christensenellaceae, Peptostreptococcaceae, Christensenellaceae R7 group, *Marvinbryantia*, Ruminococcaceae UCG005, Ruminococcaceae UCG008, Ruminococcaceae UCG010, Ruminococcaceae NK4A214 group, *Intestinibacter*, and *Romboutsia*. These findings further extend the evidence that higher abundance of butyrate-producing bacteria is associated with lower risk of type 2 diabetes. Of interest, some of these newly identified bacteria associated with type 2 diabetes have been previously reported in relation to obesity, which is closely associated with insulin resistance and development of type 2 diabetes. For example, a previous study by Goodrich et al^26^ reported that higher abundance of Christensenellaceae was linked to a lower BMI. In the current analyses, associations with insulin resistance and type 2 diabetes independent of BMI were observed, suggesting a role in the development of type 2 diabetes beyond obesity. Furthermore, this study found similar effect estimates for α diversity and type 2 diabetes when excluding patients using metformin and associations with HOMA-IR among individuals who did not use metformin, suggesting that the observed associations between gut microbiome and diabetes were not driven by use of metformin. In addition, although the observed bacteria associated with insulin resistance and type 2 diabetes were all butyrate-producing bacteria, the specific butyrate-producing bacteria that were identified differed between the insulin resistance and type 2 diabetes analysis. This finding may be explained by actual differences of gut microbiome composition among different severities of insulin resistance, by residual confounding of medication or other treatments, or by chance and small differences in effect sizes.

Possible explanations for the observed associations may involve potential beneficial effects of the butyrate that are produced by these bacteria.^6^ Butyrate is short-chain fatty acid produced from fermentation of dietary fiber.^27^ Production of butyrate in the gut and the concentrations of butyrate in the gut and circulation can be modulated by dietary means, particularly through the content and composition of fermentable dietary fiber. Butyrate has been suggested to induce beneficial metabolic effects through enhancement of mitochondrial activity, improvement of energy metabolism, activation of intestinal gluconeogenesis, and prevention of metabolic endotoxemia and inflammation via different routes of gene expression and hormone regulation.^6^ Unfortunately, stool or circulating butyrate concentrations were not measured in this study, and the study has an observational design; therefore, the role of butyrate in the observed associations could not be confirmed. Future research should validate the hypothesis of butyrate-producing bacteria affecting glucose metabolism and diabetes risk via production of butyrate.

### Strengths and Limitations

This study has several strengths. First, the use of a large, population-based, microbiome-wide association analysis (n = 2166) afforded high statistical power to pick up associations that have previously not been identified. Second, the study adjusted for various confounders in the analyses, such as alcohol use, physical activity, BMI, educational level, and smoking status. Most previous studies^2,3,4^ did not adjust for these important confounders. Third, although temporality cannot be studied in the cross-sectional design, to minimize reverse bias and potential effects of medication use, associations between gut microbiome not only with type 2 diabetes status but also with insulin resistance were examined among participants without diabetes, and similar results were observed, suggesting that microbiome composition may already play a role in earlier phases of development of type 2 diabetes.

The study also has several limitations. First, it is a cross-sectional study, and thus the ability to assess temporality and causality is limited. Second, data on the concentrations of butyrate in stool or blood samples were not available, which limited conclusions about a role of butyrate in the observed associations. Third, gut microbiome composition was determined from stool samples. Because gut microbiome composition varies throughout the gut with respect to the anatomical location along the gut and at the given site, a more complete picture of the gut microbiome might be obtained by obtaining samples from different locations along the intestines. Fourth, because of the use of 16S ribosomal RNA data, associations at species and lower levels or associations of functional profiles of gut microbiome composition could not be explored. Metagenomics approaches could overcome these limitations. Fifth, although several covariates were adjusted for, the possibility of residual confounding (eg, by occupation or annual income) could not be excluded. Moreover, gut microbiome data were not available for many of the participants of the original cohorts, which might have resulted in selection bias if associations of gut microbiome with development of type 2 diabetes differed in those included and those not included in the current analyses. In addition, to account for potential bias from missing data on covariates, multiple imputations were used, which should improve the precision of the estimated associations. Sixth, the identification of type 2 diabetes cases was based on the medical records, cases were self-reported, and/or glucose levels were measured at the research centers, and data on glycated hemoglobin and 2-hour oral glucose tolerance test were not available, and diabetes cases might be misidentified or missed. However, all potential events of type 2 diabetes were independently adjudicated by 2 study physicians and disagreements resolved by consensus with an endocrinologist. Therefore, the possibility of misidentification is extremely limited, and even if it existed, the limited misidentification or missing data should not have largely affected associations. In addition, a small sample of participants with type 2 diabetes in the LLD study limited additional analysis for gut microbiome and type 2 diabetes (eg, by metformin use). In addition, this study may be generalized to populations with similar age and races/ethnicities, but more studies in diverse populations are needed.

## Conclusions

These findings suggest that gut microbiome composition may influence the development of type 2 diabetes. An increased gut microbial diversity, along with specifically more butyrate-producing bacteria, may benefit insulin resistance and risk of type 2 diabetes. These findings may help provide new insight into causes, mechanisms, and prevention of, as well as therapy for, type 2 diabetes.

## Notes

Supplement. **eFigure 1.** Participant Selection **eFigure 2.** Taxa in the Rotterdam Study and the LifeLines-DEEP Study **eTable 1.** Characteristics of Included and Excluded Participants **eTable 2.** Associations of Taxa and Insulin Resistance **eTable 3.** Associations of Taxa and Type 2 Diabetes **eTable 4.** Associations of α and β Diversity With Insulin Resistance and Type 2 Diabetes After Additionally Adjusting for Diet Quality and Blood Pressure **eTable 5.** Statistically Significant Associations Between Taxa and Insulin Resistance After Additionally Adjusting for Diet Quality and Blood Pressure **eTable 6.** Statistically Significant Associations Between Taxa and Type 2 Diabetes After Additionally Adjusting for Diet Quality and Blood Pressure **eMethods.** Supplementary Methods
