# Errors in Key Points

**DOI:** 10.1001/jamanetworkopen.2021.28944

**Published:** 2021-09-03

In the Original Investigation titled “Association of Insulin Resistance and Type 2 Diabetes With Gut Microbial Diversity: A Microbiome-Wide Analysis From Population Studies,”^1^ published July 29, 2021, 2 sentences in the Key Points have been amended. In Findings, the new sentence now reads, “In this cross-sectional study of 2166 participants in 2 large population-based studies, higher microbiome Shannon index and richness were associated with less insulin resistance, and patients with type 2 diabetes (n = 193) had lower richness than participants without diabetes.” The sentence under Meaning now reads, “These findings suggest that gut microbial diversity, along with specifically butyrate-producing bacteria, may play a role in the development of type 2 diabetes, which may help guide future prevention and treatment strategies.” This article has been corrected.^1^
