# Supplementary material for: Association of Insulin Resistance and Type 2 Diabetes With Gut Microbial Diversity: A Microbiome-Wide Analysis From Population Studies
Source: JAMA Netw Open. 2021 Jul 29;4(7):e2118811. doi: 10.1001/jamanetworkopen.2021.18811 (PMC8322996; doi:10.1001/jamanetworkopen.2021.18811)
Supplement: Supplement. — eFigure 1. Participant Selection eFigure 2. Taxa in the Rotterdam Study and the LifeLines-DEEP Study eTable 1. Characteristics of Included and Excluded Participants eTable 2. Associations of Taxa and Insulin Resistance eTable 3. Associations of Taxa and Type 2 Diabetes eTable 4. Associations of α and β Diversity With Insulin Resistance and Type 2 Diabetes After Additionally Adjusting for Diet Quality and Blood Pressure eTable 5. Statistically Significant Associations Between Taxa and Insulin Resistance After Additionally Adjusting for Diet Quality and Blood Pressure eTable 6. Statistically Significant Associations Between Taxa and Type 2 Diabetes After Additionally Adjusting for Diet Quality and Blood Pressure eMethods. Supplementary Methods [file jamanetwopen-e2118811-s001.pdf]

Supplementary Online Content

Chen Z, Radjabzadeh D, Chen L, et al. Association of insulin resistance and type 2 diabetes with gut microbial diversity: a microbiome-wide analysis from population studies. *JAMA Netw Open*. 2021;4(7):e2118811. doi:10.1001/jamanetworkopen.2021.18811

eFigure 1. Participant Selection

eFigure 2. Taxa in the Rotterdam Study and the Lifelines-Deep Study

eTable 1. Characteristics of Included and Excluded Participants

eTable 2. Associations of Taxa and Insulin Resistance

eTable 3. Associations of Taxa and Type 2 Diabetes

eTable 4. Associations of α and β Diversity With Insulin Resistance and Type 2 Diabetes After Additionally Adjusting for Diet Quality and Blood Pressure

eTable 5. Statistically Significant Associations Between Taxa and Insulin Resistance After Additionally Adjusting for Diet Quality and Blood Pressure

eTable 6. Statistically Significant Associations Between Taxa and Type 2 Diabetes After Additionally Adjusting for Diet Quality and Blood Pressure

eMethods. Supplementary Methods

This supplementary material has been provided by the authors to give readers additional information about their work.

© 2021 Chen Z et al. *JAMA Network Open.*

eFigure 1. Participant Selection

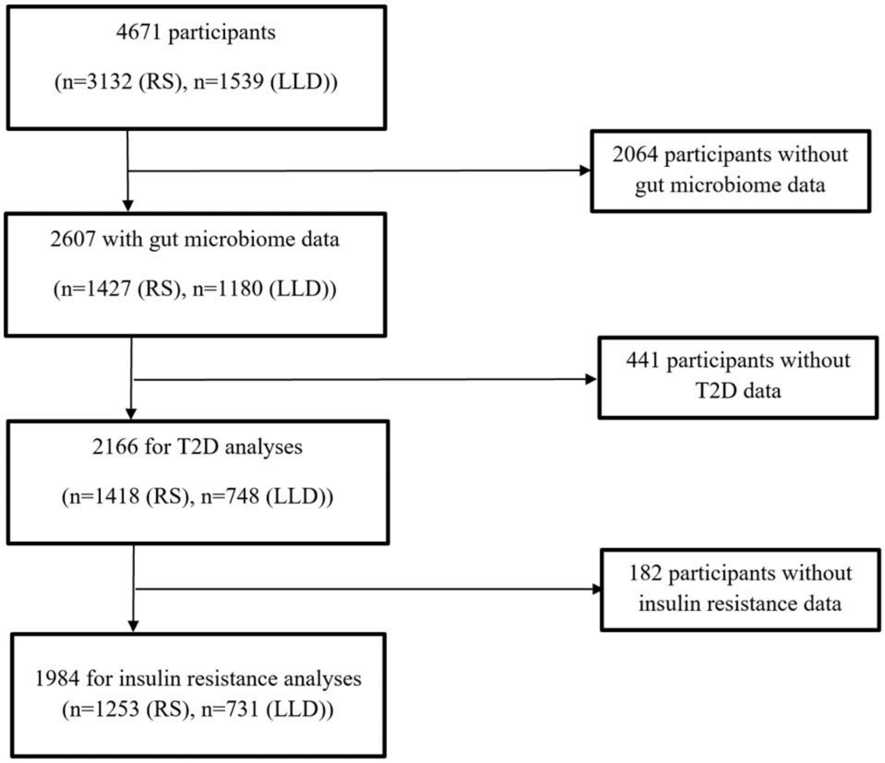

© 2021 Chen Z et al. *JAMA Network Open.*

eFigure 2. Taxa in the Rotterdam Study and the Lifelines-Deep Study

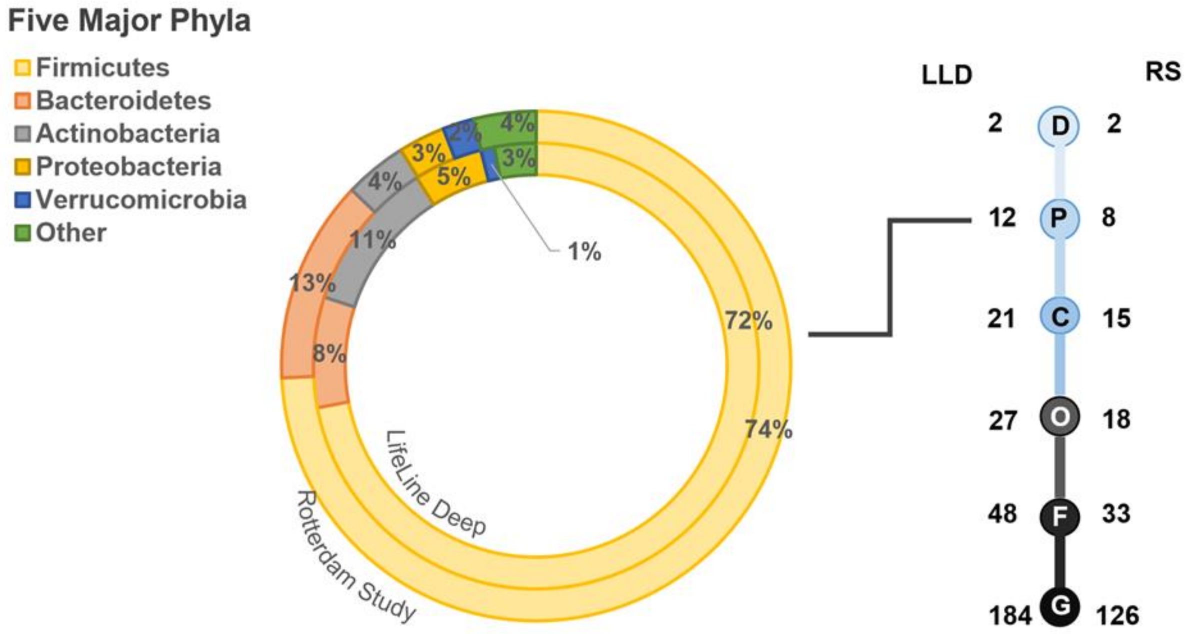

RS, Rotterdam Study; LLD, Lifelines Deep Study

D, domain; P, Phylum; C, Class; O, order; F, Family, G, Genus

© 2021 Chen Z et al. *JAMA Network Open.*

eTable 1. Characteristics of Included and Excluded Participants

**The Rotterdam Study**	**Included participants**	**Excluded participants**
	n=1,41	n= 1,714
**Age (years)**	62.4 (5.9)	61.7 (5.8)
**Sex (%)**		
-Female	815 (57.5%)	972 (56.7%)
-Male	603 (42.5%)	742 (43.3%)
**Smoking (%)**		
-Current	193 (13.6%)	499 (29.1%)
-Non-smoker		
Ever, quit	706 (49.8%)	715 (41.7%)
Never	519 (36.6%)	487 (28.4%)
**BMI (kg/m^2)**	27.5 (4.5)	27.8 (4.7)
**The Lifelines-Deep Study**	n=748	n=791
**Age (years)**	44.7 (13.4)	45.17 (14.8)
**Sex (%)**		
-Female	431(57.6%)	456 (57.7%)
-Male	317 (42.4%)	335 (42.3%)
**Smoking (%)**		
-Current	155 (20.7%)	371(46.94%)
-Non-smoker	593 (79.3%)	420(53.06%)
Ever, quit		
Never		
**BMI (kg/m^2)**	25.2 (4.1)	25.45 (4.2)

Variables expressed as mean (SD), median (25th percentile–75th percentile), or percentage.

© 2021 Chen Z et al. *JAMA Network Open.*

eTable 2. Associations of Taxa and Insulin Resistance

Effect estimates in RS and LLD were calculated using linear regression; pooled estimates were calculated based on a mixed-effect meta-analysis. Model 1: Adjusted for age, sex, Time in mail (RS), and Batch (RS).

Abbreviation, RS, Rotterdam Study, LLD, Lifelines-Deep Study. P<0.0005 indicates statistical significance

Overlapping taxa in RS and LLD	Clade	Phenotype	Beta_RS	Se_RS	Beta_LLD	Se_LLD	Beta_Meta	Lower95%CI_Meta	Upper95%CI_Meta	p_Meta	I^2
Bacteria.id.3	domain	Homa-IR	0.03	0.03	0.13	0.04	0.06	0.03	0.10	0.00	0.55
Actinobacteria.id.400	phylum	Homa-IR	0.03	0.03	-0.01	0.04	0.02	-0.03	0.06	0.48	0.07
Bacteroidetes.id.905	phylum	Homa-IR	-0.07	0.03	-0.04	0.04	-0.05	-0.10	-0.01	0.01	0.00
Cyanobacteria.id.1500	phylum	Homa-IR	-0.12	0.03	-0.03	0.04	-0.09	-0.13	-0.04	0.00	0.78
Firmicutes.id.1672	phylum	Homa-IR	0.01	0.03	0.09	0.04	0.04	0.00	0.09	0.04	0.70
Proteobacteria.id.2375	phylum	Homa-IR	-0.04	0.03	-0.02	0.04	-0.03	-0.08	0.01	0.13	0.00
Tenericutes.id.3919	phylum	Homa-IR	-0.14	0.03	-0.10	0.04	-0.13	-0.17	-0.08	0.00	0.00
Verrucomicrobia.id.3982	phylum	Homa-IR	-0.04	0.03	-0.05	0.04	-0.04	-0.09	0.00	0.05	0.00
Actinobacteria.id.419	class	Homa-IR	0.04	0.03	-0.01	0.04	0.02	-0.02	0.07	0.28	0.06
Alphaproteobacteria.id.2379	class	Homa-IR	-0.10	0.03	-0.04	0.04	-0.07	-0.12	-0.03	0.00	0.39
Bacilli.id.1673	class	Homa-IR	0.12	0.03	0.09	0.03	0.11	0.06	0.15	0.00	0.00
Bacteroidia.id.912	class	Homa-IR	-0.07	0.03	-0.04	0.04	-0.05	-0.10	-0.01	0.01	0.00
Betaproteobacteria.id.2867	class	Homa-IR	-0.01	0.03	0.00	0.04	-0.01	-0.05	0.04	0.78	0.00
Clostridia.id.1859	class	Homa-IR	-0.04	0.03	0.04	0.04	-0.01	-0.05	0.03	0.68	0.71
Coriobacteriia.id.809	class	Homa-IR	-0.02	0.03	-0.02	0.04	-0.02	-0.07	0.02	0.29	0.00
Deltaproteobacteria.id.3087	class	Homa-IR	-0.08	0.03	-0.03	0.04	-0.06	-0.10	-0.01	0.01	0.15
Erysipelotrichia.id.2147	class	Homa-IR	0.05	0.03	0.05	0.04	0.05	0.01	0.10	0.02	0.00
Gammaproteobacteria.id.3303	class	Homa-IR	-0.04	0.03	-0.01	0.04	-0.03	-0.07	0.01	0.17	0.00
Melainabacteria.id.1589	class	Homa-IR	-0.12	0.03	-0.04	0.04	-0.08	-0.13	-0.04	0.00	0.68
Mollicutes.id.3920	class	Homa-IR	-0.14	0.03	-0.10	0.04	-0.13	-0.17	-0.08	0.00	0.00
Negativicutes.id.2164	class	Homa-IR	0.02	0.03	-0.01	0.04	0.00	-0.04	0.05	0.85	0.00
Verrucomicrobiae.id.4029	class	Homa-IR	-0.03	0.03	-0.05	0.04	-0.04	-0.08	0.00	0.07	0.00
Aeromonadales.id.3316	order	Homa-IR	-0.03	0.03	0.04	0.04	0.00	-0.04	0.04	0.95	0.55
Bacteroidales.id.913	order	Homa-IR	-0.07	0.03	-0.04	0.04	-0.05	-0.10	-0.01	0.01	0.00
Bifidobacteriales.id.432	order	Homa-IR	0.03	0.03	-0.01	0.04	0.02	-0.03	0.06	0.43	0.00
Burkholderiales.id.2874	order	Homa-IR	-0.01	0.03	0.00	0.04	-0.01	-0.05	0.04	0.78	0.00
Clostridiales.id.1863	order	Homa-IR	-0.04	0.03	0.04	0.04	-0.01	-0.05	0.03	0.69	0.71

© 2021 Chen Z et al. *JAMA Network Open.*

Coriobacteriales.id.810	order	Homa-IR	-0.02	0.03	-0.02	0.04	-0.02	-0.07	0.02	0.29	0.00
Desulfovibrionales.id.3156	order	Homa-IR	-0.08	0.03	-0.03	0.04	-0.06	-0.10	-0.01	0.01	0.16

© 2021 Chen Z et al. *JAMA Network Open.*

Enterobacteriales.id.3468	order	Homa-IR	-0.03	0.03	0.00	0.04	-0.02	-0.06	0.02	0.36	0.00
Erysipelotrichales.id.2148	order	Homa-IR	0.05	0.03	0.05	0.04	0.05	0.01	0.10	0.02	0.00
Gastranaerophilales.id.1591	order	Homa-IR	-0.11	0.03	-0.04	0.04	-0.08	-0.13	-0.04	0.00	0.67
Lactobacillales.id.1800	order	Homa-IR	0.12	0.03	0.09	0.03	0.11	0.06	0.15	0.00	0.00
MollicutesRF9.id.11579	order	Homa-IR	-0.12	0.03	-0.10	0.04	-0.11	-0.15	-0.07	0.00	0.00
Pasteurellales.id.3688	order	Homa-IR	0.00	0.03	-0.06	0.04	-0.02	-0.07	0.02	0.28	0.55
Rhodospirillales.id.2667	order	Homa-IR	-0.10	0.03	-0.04	0.04	-0.07	-0.12	-0.03	0.00	0.44
Selenomonadales.id.2165	order	Homa-IR	0.02	0.03	-0.01	0.04	0.00	-0.04	0.05	0.85	0.00
Verrucomicrobiales.id.4030	order	Homa-IR	-0.03	0.03	-0.05	0.04	-0.04	-0.08	0.00	0.07	0.00
Acidaminococcaceae.id.2166	family	Homa-IR	-0.05	0.03	-0.03	0.04	-0.04	-0.09	0.00	0.05	0.00
Alcaligenaceae.id.2875	family	Homa-IR	0.00	0.03	0.02	0.04	0.01	-0.03	0.05	0.68	0.00
Bacteroidaceae.id.917	family	Homa-IR	-0.03	0.03	-0.02	0.04	-0.03	-0.07	0.02	0.24	0.00
Bifidobacteriaceae.id.433	family	Homa-IR	0.03	0.03	-0.01	0.04	0.02	-0.03	0.06	0.43	0.00
Christensenellaceae.id.1866	family	Homa-IR	-0.15	0.03	-0.14	0.04	-0.15	-0.19	-0.10	0.00	0.00
Clostridiaceae1.id.1869	family	Homa-IR	-0.03	0.03	-0.06	0.04	-0.04	-0.09	0.00	0.06	0.00
ClostridialesvadinBB60group.id.11286	family	Homa-IR	-0.15	0.03	-0.08	0.03	-0.12	-0.17	-0.08	0.00	0.61
Coriobacteriaceae.id.811	family	Homa-IR	-0.02	0.03	-0.02	0.04	-0.02	-0.07	0.02	0.29	0.00
Desulfovibrionaceae.id.3169	family	Homa-IR	-0.08	0.03	-0.03	0.04	-0.06	-0.10	-0.01	0.01	0.14
Enterobacteriaceae.id.3469	family	Homa-IR	-0.03	0.03	0.00	0.04	-0.02	-0.06	0.02	0.36	0.00
Enterococcaceae.id.1828	family	Homa-IR	0.06	0.03	0.02	0.04	0.05	0.00	0.09	0.03	0.07
Erysipelotrichaceae.id.2149	family	Homa-IR	0.05	0.03	0.05	0.04	0.05	0.01	0.10	0.02	0.00
FamilyXIII.id.1957	family	Homa-IR	-0.10	0.03	-0.02	0.04	-0.07	-0.12	-0.03	0.00	0.70
Lachnospiraceae.id.1987	family	Homa-IR	0.06	0.03	0.08	0.04	0.07	0.02	0.11	0.00	0.00
Lactobacillaceae.id.1836	family	Homa-IR	0.06	0.03	0.04	0.04	0.06	0.01	0.10	0.01	0.00
Pasteurellaceae.id.3689	family	Homa-IR	0.00	0.03	-0.06	0.04	-0.02	-0.07	0.02	0.28	0.55
Peptococcaceae.id.2024	family	Homa-IR	-0.06	0.03	0.01	0.04	-0.03	-0.07	0.01	0.16	0.63
Peptostreptococcaceae.id.2042	family	Homa-IR	-0.03	0.03	0.02	0.04	-0.01	-0.05	0.03	0.67	0.00
Porphyromonadaceae.id.943	family	Homa-IR	-0.09	0.03	-0.08	0.04	-0.09	-0.13	-0.04	0.00	0.00
Prevotellaceae.id.960	family	Homa-IR	-0.02	0.03	0.03	0.04	0.00	-0.05	0.04	0.89	0.18
Rhodospirillaceae.id.2717	family	Homa-IR	-0.10	0.03	-0.04	0.04	-0.07	-0.12	-0.03	0.00	0.45
Rikenellaceae.id.967	family	Homa-IR	-0.13	0.03	-0.11	0.03	-0.12	-0.16	-0.08	0.00	0.00
Ruminococcaceae.id.2050	family	Homa-IR	-0.12	0.03	0.00	0.04	-0.07	-0.12	-0.03	0.00	0.85

© 2021 Chen Z et al. *JAMA Network Open.*

Streptococcaceae.id.1850	family	Homa-IR	0.11	0.03	0.09	0.03	0.10	0.06	0.15	0.00	0.00
Succinivibrionaceae.id.3326	family	Homa-IR	-0.03	0.03	0.04	0.04	0.00	-0.04	0.04	0.93	0.56

© 2021 Chen Z et al. *JAMA Network Open.*

Veillonellaceae.id.2172	family	Homa-IR	0.08	0.03	-0.01	0.04	0.04	0.00	0.09	0.04	0.71
Verrucomicrobiaceae.id.4036	family	Homa-IR	-0.03	0.03	-0.05	0.04	-0.04	-0.08	0.00	0.07	0.00
unknownfamily.id.1000001214	family	Homa-IR	-0.11	0.03	-0.04	0.04	-0.08	-0.13	-0.04	0.00	0.67
unknownfamily.id.1000005471	family	Homa-IR	-0.12	0.03	-0.10	0.04	-0.11	-0.15	-0.07	0.00	0.00
unknownfamily.id.987	family	Homa-IR	-0.01	0.03	-0.05	0.04	-0.03	-0.07	0.02	0.23	0.00
Bacteroidespectinophilusgroup.id.14371	genus	Homa-IR	-0.07	0.03	-0.03	0.04	-0.05	-0.10	-0.01	0.02	0.00
Eubacteriumcoprostanoligenesgroup.id.11375	genus	Homa-IR	-0.03	0.03	0.02	0.04	-0.01	-0.05	0.03	0.66	0.08
Eubacteriumeligensgroup.id.14372	genus	Homa-IR	-0.11	0.03	-0.03	0.04	-0.08	-0.12	-0.04	0.00	0.66
Eubacteriumhalliigroup.id.11338	genus	Homa-IR	0.08	0.03	0.03	0.04	0.06	0.02	0.10	0.01	0.29
Eubacteriumrectalegroup.id.14374	genus	Homa-IR	0.07	0.03	0.07	0.04	0.07	0.03	0.11	0.00	0.00
Eubacteriumruminantiumgroup.id.11340	genus	Homa-IR	-0.06	0.03	-0.05	0.04	-0.06	-0.10	-0.02	0.01	0.00
Eubacteriumventriosumgroup.id.11341	genus	Homa-IR	0.02	0.03	0.07	0.04	0.04	0.00	0.08	0.08	0.27
Eubacteriumxylanophilumgroup.id.14375	genus	Homa-IR	-0.12	0.03	-0.04	0.04	-0.09	-0.13	-0.05	0.00	0.72
Ruminococcusgauvreauiigroup.id.11342	genus	Homa-IR	0.02	0.03	0.10	0.03	0.05	0.00	0.09	0.03	0.68
Ruminococcusgnavusgroup.id.14376	genus	Homa-IR	0.04	0.03	0.11	0.03	0.07	0.03	0.11	0.00	0.49
Ruminococcustorquesgroup.id.14377	genus	Homa-IR	0.06	0.03	0.02	0.04	0.05	0.00	0.09	0.04	0.00
Acidaminococcus.id.2167	genus	Homa-IR	0.07	0.03	0.06	0.03	0.07	0.02	0.11	0.00	0.00
Adlercreutzia.id.812	genus	Homa-IR	0.05	0.03	0.04	0.04	0.04	0.00	0.09	0.05	0.00
Akkermansia.id.4037	genus	Homa-IR	-0.03	0.03	-0.05	0.04	-0.04	-0.08	0.00	0.07	0.00
Alistipes.id.968	genus	Homa-IR	-0.13	0.03	-0.10	0.03	-0.12	-0.16	-0.07	0.00	0.00
Alloprevotella.id.961	genus	Homa-IR	-0.03	0.03	0.03	0.04	0.00	-0.05	0.04	0.83	0.49
Anaerostipes.id.1991	genus	Homa-IR	0.00	0.03	0.03	0.04	0.01	-0.03	0.06	0.54	0.00
Anaerotruncus.id.2054	genus	Homa-IR	-0.13	0.03	-0.05	0.04	-0.10	-0.14	-0.05	0.00	0.60
Bacteroides.id.918	genus	Homa-IR	-0.03	0.03	-0.02	0.04	-0.03	-0.07	0.02	0.24	0.00
Barnesiella.id.944	genus	Homa-IR	-0.08	0.03	-0.03	0.04	-0.06	-0.10	-0.02	0.01	0.00
Bifidobacterium.id.436	genus	Homa-IR	0.03	0.03	0.00	0.04	0.02	-0.02	0.06	0.37	0.00
Blautia.id.1992	genus	Homa-IR	0.06	0.03	0.06	0.04	0.06	0.02	0.11	0.00	0.00
Butyricicoccus.id.2055	genus	Homa-IR	0.03	0.03	-0.01	0.04	0.01	-0.03	0.05	0.61	0.00
Butyricimonas.id.945	genus	Homa-IR	-0.07	0.03	-0.06	0.04	-0.07	-0.11	-0.02	0.00	0.00
Butyrivibrio.id.1993	genus	Homa-IR	-0.09	0.03	-0.03	0.04	-0.07	-0.11	-0.02	0.00	0.37
Catenibacterium.id.2153	genus	Homa-IR	0.05	0.03	0.01	0.04	0.03	-0.01	0.07	0.15	0.00
ChristensenellaceaeR7group.id.11283	genus	Homa-IR	-0.14	0.03	-0.13	0.04	-0.14	-0.18	-0.09	0.00	0.00

© 2021 Chen Z et al. *JAMA Network Open.*

Clostridiumsensustricto1.id.1873	genus	Homa-IR	-0.03	0.03	-0.06	0.04	-0.04	-0.09	0.00	0.05	0.00
Collinsella.id.815	genus	Homa-IR	0.04	0.03	-0.01	0.04	0.02	-0.02	0.07	0.32	0.12

© 2021 Chen Z et al. *JAMA Network Open.*

Coprococcus1.id.11301		genus	Homa-IR	-0.08	0.03	-0.05	0.04	-0.07	-0.11	-0.03	0.00	0.00
Coprococcus2.id.11302		genus	Homa-IR	-0.08	0.03	-0.04	0.04	-0.07	-0.11	-0.02	0.00	0.00
Coprococcus3.id.11303		genus	Homa-IR	0.02	0.03	0.02	0.04	0.02	-0.02	0.06	0.38	0.00
Desulfovibrio.id.3173		genus	Homa-IR	-0.08	0.03	-0.03	0.04	-0.06	-0.10	-0.02	0.01	0.33
Dialister.id.2183		genus	Homa-IR	0.06	0.03	-0.02	0.04	0.03	-0.02	0.07	0.22	0.63
Dorea.id.1997		genus	Homa-IR	0.04	0.03	-0.02	0.04	0.02	-0.03	0.06	0.49	0.45
Eggerthella.id.819		genus	Homa-IR	0.00	0.03	0.09	0.03	0.04	-0.01	0.08	0.09	0.74
Eisenbergiella.id.11304		genus	Homa-IR	-0.04	0.03	0.01	0.04	-0.02	-0.07	0.02	0.28	0.25
Enterococcus.id.1831		genus	Homa-IR	0.06	0.03	0.03	0.04	0.05	0.01	0.09	0.03	0.00
Enterorhabdus.id.820		genus	Homa-IR	-0.08	0.03	-0.06	0.04	-0.07	-0.11	-0.03	0.00	0.00
Erysipelatoclostridium.id.11381		genus	Homa-IR	-0.01	0.03	0.05	0.04	0.01	-0.03	0.05	0.62	0.40
ErysipelotrichaceaeUCG003.id.11384		genus	Homa-IR	0.00	0.03	0.02	0.04	0.01	-0.03	0.06	0.57	0.00
Faecalibacterium.id.2057		genus	Homa-IR	-0.03	0.03	0.04	0.04	0.00	-0.05	0.04	0.88	0.61
FamilyXIIIAD3011group.id.11293		genus	Homa-IR	-0.12	0.03	-0.11	0.03	-0.12	-0.16	-0.07	0.00	0.00
FamilyXIIIUCG001.id.11294		genus	Homa-IR	-0.04	0.03	-0.04	0.04	-0.04	-0.09	0.00	0.05	0.00
Fusicatenibacter.id.11305		genus	Homa-IR	0.05	0.03	0.05	0.04	0.05	0.01	0.09	0.02	0.00
Haemophilus.id.3698		genus	Homa-IR	0.00	0.03	-0.06	0.04	-0.02	-0.06	0.02	0.32	0.43
Holdemanella.id.11393		genus	Homa-IR	0.04	0.03	0.06	0.04	0.05	0.01	0.09	0.03	0.00
Intestinibacter.id.11345		genus	Homa-IR	0.00	0.03	0.03	0.04	0.01	-0.03	0.05	0.61	0.00
Intestinimonas.id.2062		genus	Homa-IR	-0.08	0.03	-0.05	0.04	-0.07	-0.11	-0.03	0.00	0.00
Lachnoclostridium.id.11308		genus	Homa-IR	0.01	0.03	0.09	0.04	0.04	0.00	0.09	0.05	0.67
Lachnospira.id.2004		genus	Homa-IR	-0.02	0.03	0.02	0.04	0.00	-0.05	0.04	0.91	0.00
LachnospiraceaeFCS020group.id.11314		genus	Homa-IR	-0.03	0.03	-0.08	0.03	-0.05	-0.09	-0.01	0.02	0.35
LachnospiraceaeND3007group.id.11317		genus	Homa-IR	-0.05	0.03	-0.01	0.04	-0.04	-0.08	0.01	0.08	0.00
LachnospiraceaeNK4A136group.id.11319		genus	Homa-IR	-0.07	0.03	0.01	0.04	-0.04	-0.08	0.00	0.08	0.69
LachnospiraceaeUCG001.id.11321		genus	Homa-IR	-0.02	0.03	0.01	0.04	-0.01	-0.05	0.03	0.70	0.00
LachnospiraceaeUCG004.id.11324		genus	Homa-IR	-0.04	0.03	-0.04	0.04	-0.04	-0.09	0.00	0.04	0.00
LachnospiraceaeUCG010.id.11330		genus	Homa-IR	-0.02	0.03	-0.06	0.03	-0.04	-0.08	0.01	0.09	0.00
Lactobacillus.id.1837		genus	Homa-IR	0.07	0.03	0.05	0.04	0.06	0.02	0.11	0.00	0.00
Marvinbryantia.id.2005		genus	Homa-IR	-0.12	0.03	-0.08	0.04	-0.11	-0.15	-0.06	0.00	0.00
Megamonas.id.2184		genus	Homa-IR	0.03	0.03	-0.01	0.04	0.02	-0.03	0.06	0.45	0.00
Megasphaera.id.2185		genus	Homa-IR	0.01	0.03	0.06	0.03	0.03	-0.01	0.07	0.15	0.14

© 2021 Chen Z et al. *JAMA Network Open.*

Mitsuokella.id.2186	genus	Homa-IR	0.05	0.03	0.02	0.04	0.04	0.00	0.08	0.06	0.00
Odoribacter.id.952	genus	Homa-IR	-0.09	0.03	-0.09	0.04	-0.09	-0.13	-0.05	0.00	0.00

© 2021 Chen Z et al. *JAMA Network Open.*

Olsenella.id.822	genus	Homa-IR	0.02	0.03	-0.05	0.04	-0.01	-0.05	0.03	0.66	0.56
Oscillospira.id.2064	genus	Homa-IR	-0.09	0.03	-0.05	0.04	-0.07	-0.12	-0.03	0.00	0.00
Parabacteroides.id.954	genus	Homa-IR	-0.07	0.03	-0.07	0.04	-0.07	-0.11	-0.03	0.00	0.00
Paraprevotella.id.962	genus	Homa-IR	-0.04	0.03	-0.05	0.04	-0.04	-0.09	0.00	0.05	0.00
Parasutterella.id.2892	genus	Homa-IR	0.01	0.03	0.06	0.04	0.03	-0.01	0.08	0.13	0.21
Peptococcus.id.2037	genus	Homa-IR	-0.02	0.03	0.02	0.04	0.00	-0.05	0.04	0.85	0.00
Phascolarctobacterium.id.2168	genus	Homa-IR	-0.06	0.03	-0.04	0.04	-0.06	-0.10	-0.01	0.01	0.00
Prevotella2.id.11180	genus	Homa-IR	0.01	0.03	-0.02	0.04	0.00	-0.05	0.04	0.90	0.00
Prevotella7.id.11182	genus	Homa-IR	0.01	0.03	0.03	0.04	0.02	-0.02	0.06	0.39	0.00
Prevotella9.id.11183	genus	Homa-IR	-0.02	0.03	0.02	0.04	0.00	-0.04	0.04	0.94	0.00
PrevotellaceaeNK3B31group.id.11185	genus	Homa-IR	0.00	0.03	0.02	0.04	0.01	-0.04	0.05	0.78	0.00
PrevotellaceaeUCG001.id.11186	genus	Homa-IR	-0.06	0.03	0.03	0.04	-0.03	-0.07	0.02	0.22	0.76
RikenellaceaeRC9gutgroup.id.11191	genus	Homa-IR	-0.01	0.03	-0.05	0.04	-0.03	-0.07	0.02	0.22	0.00
Romboutsia.id.11347	genus	Homa-IR	-0.03	0.03	0.01	0.04	-0.01	-0.06	0.03	0.53	0.06
Roseburia.id.2012	genus	Homa-IR	0.02	0.03	0.04	0.04	0.03	-0.02	0.07	0.23	0.00
Ruminiclostridium5.id.11355	genus	Homa-IR	-0.09	0.03	0.01	0.04	-0.05	-0.09	-0.01	0.02	0.79
Ruminiclostridium6.id.11356	genus	Homa-IR	-0.15	0.03	-0.03	0.04	-0.10	-0.15	-0.06	0.00	0.87
Ruminiclostridium9.id.11357	genus	Homa-IR	-0.09	0.03	-0.14	0.03	-0.11	-0.15	-0.06	0.00	0.29
RuminococcaceaeNK4A214group.id.11358	genus	Homa-IR	-0.16	0.03	-0.15	0.03	-0.15	-0.20	-0.11	0.00	0.00
RuminococcaceaeUCG002.id.11360	genus	Homa-IR	-0.12	0.03	-0.13	0.03	-0.12	-0.17	-0.08	0.00	0.00
RuminococcaceaeUCG003.id.11361	genus	Homa-IR	-0.06	0.03	-0.12	0.03	-0.09	-0.13	-0.04	0.00	0.39
RuminococcaceaeUCG004.id.11362	genus	Homa-IR	0.00	0.03	-0.01	0.04	-0.01	-0.05	0.04	0.78	0.00
RuminococcaceaeUCG005.id.11363	genus	Homa-IR	-0.16	0.03	-0.11	0.04	-0.14	-0.18	-0.10	0.00	0.32
RuminococcaceaeUCG008.id.11365	genus	Homa-IR	-0.12	0.03	-0.07	0.04	-0.10	-0.14	-0.06	0.00	0.33
RuminococcaceaeUCG010.id.11367	genus	Homa-IR	-0.17	0.03	-0.14	0.03	-0.16	-0.20	-0.12	0.00	0.00
RuminococcaceaeUCG013.id.11370	genus	Homa-IR	0.00	0.03	0.08	0.04	0.03	-0.01	0.08	0.14	0.67
RuminococcaceaeUCG014.id.11371	genus	Homa-IR	-0.13	0.03	-0.10	0.04	-0.12	-0.16	-0.08	0.00	0.00
Ruminococcus1.id.11373	genus	Homa-IR	-0.09	0.03	0.05	0.04	-0.03	-0.07	0.01	0.15	0.90
Ruminococcus2.id.11374	genus	Homa-IR	0.02	0.03	0.05	0.04	0.03	-0.01	0.07	0.15	0.00
Sellimonas.id.14369	genus	Homa-IR	0.04	0.03	0.06	0.04	0.05	0.00	0.09	0.03	0.00
Senegalimassilia.id.11160	genus	Homa-IR	-0.01	0.03	-0.07	0.03	-0.03	-0.07	0.01	0.16	0.39
Slackia.id.825	genus	Homa-IR	0.05	0.03	-0.07	0.04	0.00	-0.04	0.05	0.83	0.85

© 2021 Chen Z et al. *JAMA Network Open.*

Streptococcus.id.1853	genus	Homa-IR	0.12	0.03	0.09	0.03	0.11	0.06	0.15	0.00	0.00
Subdoligranulum.id.2070	genus	Homa-IR	-0.01	0.03	0.02	0.04	0.00	-0.04	0.04	0.98	0.00

© 2021 Chen Z et al. *JAMA Network Open.*

Succiniclasticum.id.2169	genus	Homa-IR	-0.01	0.03	-0.01	0.04	-0.01	-0.05	0.04	0.75	0.00
Sutterella.id.2896	genus	Homa-IR	-0.02	0.03	-0.05	0.04	-0.03	-0.08	0.01	0.15	0.00
Terrisporobacter.id.11348	genus	Homa-IR	-0.04	0.03	-0.03	0.04	-0.04	-0.08	0.01	0.11	0.00
Turicibacter.id.2162	genus	Homa-IR	-0.04	0.03	-0.06	0.04	-0.05	-0.09	0.00	0.03	0.00
Tyzzerella3.id.11335	genus	Homa-IR	0.06	0.03	0.02	0.04	0.04	0.00	0.09	0.04	0.00
Veillonella.id.2198	genus	Homa-IR	0.03	0.03	-0.03	0.04	0.01	-0.04	0.05	0.77	0.47
unknowngenus.id.1000000073	genus	Homa-IR	-0.15	0.03	-0.08	0.03	-0.12	-0.17	-0.08	0.00	0.61
unknowngenus.id.1000001215	genus	Homa-IR	-0.11	0.03	-0.04	0.04	-0.08	-0.13	-0.04	0.00	0.67
unknowngenus.id.1000005472	genus	Homa-IR	-0.12	0.03	-0.10	0.04	-0.11	-0.15	-0.07	0.00	0.00
unknowngenus.id.1000005479	genus	Homa-IR	-0.08	0.03	-0.04	0.04	-0.06	-0.11	-0.02	0.00	0.00
unknowngenus.id.1000013899	genus	Homa-IR	-0.01	0.03	-0.05	0.04	-0.03	-0.07	0.02	0.23	0.00
unknowngenus.id.2071	genus	Homa-IR	-0.15	0.03	-0.05	0.04	-0.11	-0.16	-0.07	0.00	0.81
unknowngenus.id.2755	genus	Homa-IR	-0.10	0.03	-0.03	0.04	-0.07	-0.11	-0.03	0.00	0.46
unknowngenus.id.826	genus	Homa-IR	-0.07	0.03	-0.02	0.04	-0.05	-0.09	-0.01	0.02	0.29
unknowngenus.id.964	genus	Homa-IR	0.03	0.03	0.01	0.04	0.02	-0.02	0.07	0.30	0.00

**Taxa in RS only**	**Clade**	**Phenotype**	**Beta_RS**	**Se_RS**
BacteroidalesS247group.id.11173	family	Homa-IR	-0.08	0.03
Veillonellaceae.id.2172	family	Homa-IR	0.08	0.03
Bacteroidespectinophilusgroup.id.14371	genus	Homa-IR	-0.07	0.03
Enterobacter.id.3502	genus	Homa-IR	0.08	0.03
EscherichiaShigella.id.3504	genus	Homa-IR	-0.04	0.03
Eubacteriumcoprostanoligenesgroup.id.11375	genus	Homa-IR	-0.03	0.03
Eubacteriumeligensgroup.id.14372	genus	Homa-IR	-0.11	0.03
Eubacteriumhalliigroup.id.11338	genus	Homa-IR	0.08	0.03
Eubacteriumoxidoreducensgroup.id.11339	genus	Homa-IR	-0.17	0.03
Eubacteriumxylanophilumgroup.id.14375	genus	Homa-IR	-0.12	0.03
Hungatella.id.11306	genus	Homa-IR	-0.04	0.03
Klebsiella.id.3507	genus	Homa-IR	0.05	0.03
LachnospiraceaeNC2004group.id.11316	genus	Homa-IR	-0.12	0.03

© 2021 Chen Z et al. *JAMA Network Open.*

LachnospiraceaeUCG008.id.11328	genus	Homa-IR	-0.06	0.03
unknowngenus.id.1868	genus	Homa-IR	-0.13	0.03

© 2021 Chen Z et al. *JAMA Network Open.*

Taxa in LLD only	Clade	Phenotype	Beta_LLD	Se_LLD
Archaea.id.2	domain	Homa-IR	-0.13	0.04
Euryarchaeota.id.55	phylum	Homa-IR	-0.13	0.04
Lentisphaerae.id.2238	phylum	Homa-IR	-0.05	0.04
Actinomycetales.id.420	order	Homa-IR	0.04	0.04
Mycoplasmatales.id.3946	order	Homa-IR	-0.03	0.04
NB1.n.id.3953	order	Homa-IR	-0.05	0.04
Pseudomonadales.id.3709	order	Homa-IR	-0.02	0.04
Spirochaetales.id.3870	order	Homa-IR	-0.02	0.04
Synergistales.id.3900	order	Homa-IR	-0.05	0.04
Thermoplasmatales.id.220	order	Homa-IR	-0.03	0.04
unknownorder.id.1000000003	order	Homa-IR	0.01	0.04
Verrucomicrobiales.id.4030	order	Homa-IR	-0.05	0.04
Victivallales.id.2254	order	Homa-IR	-0.05	0.04
Xanthomonadales.id.3786	order	Homa-IR	-0.02	0.04
Spirochaetes.id.3856	class	Homa-IR	-0.02	0.04
Synergistia.id.3899	class	Homa-IR	-0.05	0.04
Thermoplasmata.id.210	class	Homa-IR	-0.03	0.04
Actinomycetaceae.id.421	family	Homa-IR	0.04	0.04
BacteroidalesS24.7group.id.11173	family	Homa-IR	-0.04	0.04
Christensenellaceae.id.1866	family	Homa-IR	-0.14	0.04
FamilyXI.id.1936	family	Homa-IR	0.00	0.04
unknownfamily.id.1000000004	family	Homa-IR	0.01	0.04
unknownfamily.id.1000006161	family	Homa-IR	-0.05	0.04
unknownfamily.id.1855	family	Homa-IR	-0.01	0.04
vadinBE97.id.14446	family	Homa-IR	-0.03	0.04
Veillonellaceae.id.2172	family	Homa-IR	-0.01	0.04
Xanthomonadaceae.id.3799	family	Homa-IR	-0.02	0.04
Clostridiuminnocuumgroup.id.14397	genus	Homa-IR	0.04	0.04
Eubacteriumbrachygroup.id.11296	genus	Homa-IR	0.07	0.04
Abiotrophia.id.1803	genus	Homa-IR	0.01	0.04
Actinomyces.id.423	genus	Homa-IR	0.06	0.04

© 2021 Chen Z et al. *JAMA Network Open.*

Actinotignum.id.11137	genus	Homa-IR	-0.04	0.04
Allisonella.id.2174	genus	Homa-IR	0.10	0.03

© 2021 Chen Z et al. *JAMA Network Open.*

Arcanobacterium.id.424	genus	Homa-IR	0.00	0.04
Asaccharobacter.id.813	genus	Homa-IR	0.03	0.04
Bilophila.id.3170	genus	Homa-IR	-0.02	0.04
CandidatusMethanogranum.id.11110	genus	Homa-IR	-0.01	0.04
Catenisphaera.id.14395	genus	Homa-IR	0.00	0.04
Cellulosilyticum.id.1995	genus	Homa-IR	-0.06	0.04
Cetobacterium.id.2209	genus	Homa-IR	0.06	0.04
Cloacibacillus.id.3908	genus	Homa-IR	-0.03	0.04
Coprobacillus.id.2154	genus	Homa-IR	0.03	0.04
CoriobacteriaceaeUCG.002.id.11158	genus	Homa-IR	-0.05	0.04
CoriobacteriaceaeUCG.003.id.11159	genus	Homa-IR	0.05	0.04
Denitrobacterium.id.818	genus	Homa-IR	-0.02	0.04
dgA.11gutgroup.id.978	genus	Homa-IR	0.03	0.04
Dielma.id.11380	genus	Homa-IR	-0.01	0.04
Epulopiscium.id.1998	genus	Homa-IR	0.04	0.04
ErysipelotrichaceaeUCG006.id.11386	genus	Homa-IR	0.01	0.04
ErysipelotrichaceaeUCG009.id.11389	genus	Homa-IR	0.05	0.04
Escherichia.Shigella.id.3504	genus	Homa-IR	0.00	0.04
Eubacterium.id.1932	genus	Homa-IR	0.06	0.04
Faecalicoccus.id.11391	genus	Homa-IR	-0.01	0.04
Faecalitalea.id.11392	genus	Homa-IR	0.05	0.04
Flavonifractor.id.2059	genus	Homa-IR	0.01	0.04
Gardnerella.id.437	genus	Homa-IR	0.01	0.04
Gordonibacter.id.821	genus	Homa-IR	0.01	0.04
Hafnia.Obesumbacterium.id.14636	genus	Homa-IR	0.01	0.04
Howardella.id.2000	genus	Homa-IR	0.00	0.04
LachnospiraceaeNK3A20group.id.11318	genus	Homa-IR	0.01	0.04
Lactococcus.id.1851	genus	Homa-IR	-0.03	0.04
Lautropia.id.2905	genus	Homa-IR	0.01	0.04
Leuconostoc.id.1841	genus	Homa-IR	0.01	0.04
Methanobrevibacter.id.123	genus	Homa-IR	-0.12	0.04
Methanosphaera.id.124	genus	Homa-IR	-0.07	0.04

© 2021 Chen Z et al. *JAMA Network Open.*

Mogibacterium.id.1960	genus	Homa-IR	0.00	0.04
Morganella.id.3512	genus	Homa-IR	0.04	0.04

© 2021 Chen Z et al. *JAMA Network Open.*

Porphyromonas.id.956	genus	Homa-IR	-0.01	0.04
Prevotella1.id.11179	genus	Homa-IR	0.02	0.04
PrevotellaceaeUCG003.id.11187	genus	Homa-IR	0.07	0.03
PrevotellaceaeUCG004.id.11188	genus	Homa-IR	-0.06	0.04
Pseudomonas.id.3723	genus	Homa-IR	-0.02	0.04
Rikenella.id.973	genus	Homa-IR	0.01	0.04
RuminococcaceaeNK4A214group.id.11358	genus	Homa-IR	-0.15	0.03
RuminococcaceaeUCG005.id.11363	genus	Homa-IR	-0.11	0.04
RuminococcaceaeUCG008.id.11365	genus	Homa-IR	-0.07	0.04
RuminococcaceaeUCG010.id.11367	genus	Homa-IR	-0.14	0.03
Sarcina.id.1896	genus	Homa-IR	0.06	0.04
Solobacterium.id.2161	genus	Homa-IR	0.05	0.04
Staphylococcus.id.1780	genus	Homa-IR	0.04	0.04
Stenotrophomonas.id.3818	genus	Homa-IR	-0.02	0.04
Succinivibrio.id.3331	genus	Homa-IR	0.04	0.04
Synergistes.id.3913	genus	Homa-IR	-0.03	0.04
Syntrophococcus.id.2016	genus	Homa-IR	0.00	0.04
Treponema2.id.11577	genus	Homa-IR	-0.02	0.04
Tyzzerella4.id.11336	genus	Homa-IR	0.04	0.04
unknowngenus.id.1000000005	genus	Homa-IR	0.01	0.04
unknowngenus.id.1000027565	genus	Homa-IR	-0.03	0.04
unknowngenus.id.1000043796	genus	Homa-IR	-0.01	0.04
unknowngenus.id.1854	genus	Homa-IR	-0.02	0.04
unknowngenus.id.2163	genus	Homa-IR	-0.05	0.04
unknowngenus.id.3332	genus	Homa-IR	0.05	0.04
unknowngenus.id.441	genus	Homa-IR	-0.08	0.04
Ureaplasma.id.3951	genus	Homa-IR	-0.03	0.04
Varibaculum.id.427	genus	Homa-IR	0.00	0.04
Veillonella.id.2198	genus	Homa-IR	-0.03	0.04
Weissella.id.1843	genus	Homa-IR	0.00	0.04

© 2021 Chen Z et al. *JAMA Network Open.*

Supplementary Table 2 - Associations of taxa and insulin resistance

Effect estimates in RS and LLD were calculated using linear regression; pooled estimates were calculated based on a mixed-effect meta-analysis. Model 2: Adjusted for age, sex, Time in mail (RS), Batch (RS), smoking, education level (RS), physical activity, alcohol intake, and total energy intake. Abbreviation, RS, Rotterdam Study, LLD, Lifelines-Deep Study. P<0.0005 indicates statistical significance

Overlapping taxa in RS and LLD	Clade	Phenotype	Beta_RS	Se_RS	Beta_LLD	Se_LLD	Beta_Meta	Lower95%CI_Meta	Upper95%CI_Meta	p_Meta	I^2
Bacteria.id.3	domai	Homa-	0.0	0.0	0.1	0.03	0.05	0.02	0.09	0.0	0.
Actinobacteria.id.400	phylu	Homa-	0.0	0.0	-	0.03	0.00	-0.04	0.05	0.8	0.
Bacteroidetes.id.905	phylu	Homa-	-	0.0	-	0.03	-0.05	-0.10	-0.01	0.0	0.
Cyanobacteria.id.1500	phylu	Homa-	-	0.0	-	0.03	-0.08	-0.13	-0.04	0.0	0.
Firmicutes.id.1672	phylu	Homa-	0.0	0.0	0.1	0.03	0.05	0.01	0.10	0.0	0.
Proteobacteria.id.2375	phylu	Homa-	-	0.0	-	0.03	-0.03	-0.08	0.02	0.2	0.
Tenericutes.id.3919	phylu	Homa-	-	0.0	-	0.03	-0.12	-0.17	-0.08	0.0	0.
Verrucomicrobia.id.3982	phylu	Homa-	-	0.0	-	0.03	-0.03	-0.08	0.01	0.1	0.
Actinobacteria.id.419	class	Homa-	0.0	0.0	-	0.04	0.01	-0.04	0.05	0.7	0.
Alphaproteobacteria.id.2379	class	Homa-	-	0.0	-	0.03	-0.08	-0.13	-0.04	0.0	0.
Bacilli.id.1673	class	Homa-	0.0	0.0	0.0	0.03	0.08	0.03	0.12	0.0	0.
Bacteroidia.id.912	class	Homa-	-	0.0	-	0.03	-0.05	-0.10	-0.01	0.0	0.
Betaproteobacteria.id.2867	class	Homa-	-	0.0	0.0	0.03	-0.01	-0.05	0.04	0.7	0.
Clostridia.id.1859	class	Homa-	-	0.0	0.0	0.03	0.00	-0.04	0.05	0.9	0.
Coriobacteriia.id.809	class	Homa-	-	0.0	-	0.03	-0.01	-0.06	0.03	0.6	0.
Deltaproteobacteria.id.3087	class	Homa-	-	0.0	-	0.03	-0.04	-0.09	0.00	0.0	0.
Erysipelotrichia.id.2147	class	Homa-	0.0	0.0	0.0	0.03	0.07	0.03	0.12	0.0	0.
Gammaproteobacteria.id.3303	class	Homa-	-	0.0	0.0	0.03	-0.03	-0.07	0.02	0.2	0.
Melainabacteria.id.1589	class	Homa-	-	0.0	-	0.03	-0.08	-0.13	-0.04	0.0	0.
Mollicutes.id.3920	class	Homa-	-	0.0	-	0.03	-0.12	-0.17	-0.08	0.0	0.
Negativicutes.id.2164	class	Homa-	0.0	0.0	-	0.03	-0.01	-0.05	0.04	0.8	0.
Verrucomicrobiae.id.4029	class	Homa-	-	0.0	-	0.03	-0.03	-0.08	0.01	0.1	0.
Aeromonadales.id.3316	orde	Homa-	-	0.0	0.0	0.03	0.01	-0.04	0.05	0.7	0.
Bacteroidales.id.913	orde	Homa-	-	0.0	-	0.03	-0.05	-0.10	-0.01	0.0	0.
Bifidobacteriales.id.432	orde	Homa-	0.0	0.0	-	0.04	0.00	-0.05	0.05	0.9	0.
Burkholderiales.id.2874	orde	Homa-	-	0.0	0.0	0.03	-0.01	-0.05	0.04	0.7	0.
Clostridiales.id.1863	orde	Homa-	-	0.0	0.0	0.03	0.00	-0.04	0.05	0.9	0.
Coriobacteriales.id.810	orde	Homa-	-	0.0	-	0.03	-0.01	-0.06	0.03	0.6	0.
Desulfovibrionales.id.3156	orde	Homa-	-	0.0	-	0.03	-0.04	-0.09	0.00	0.0	0.
Enterobacteriales.id.3468	orde	Homa-	-	0.0	0.0	0.03	-0.01	-0.06	0.03	0.5	0.
Erysipelotrichales.id.2148	orde	Homa-	0.0	0.0	0.0	0.03	0.07	0.03	0.12	0.0	0.
Gastranaerophilales.id.1591	orde	Homa-	-	0.0	-	0.03	-0.08	-0.13	-0.04	0.0	0.
Lactobacillales.id.1800	orde	Homa-	0.0	0.0	0.0	0.03	0.08	0.03	0.12	0.0	0.
MollicutesRF9.id.11579	orde	Homa-	-	0.0	-	0.03	-0.11	-0.15	-0.06	0.0	0.
Pasteurellales.id.3688	orde	Homa-	-	0.0	-	0.03	-0.03	-0.08	0.01	0.1	0.
Rhodospirillales.id.2667	orde	Homa-	-	0.0	-	0.03	-0.08	-0.13	-0.04	0.0	0.
Selenomonadales.id.2165	orde	Homa-	0.0	0.0	-	0.03	-0.01	-0.05	0.04	0.8	0.
Verrucomicrobiales.id.4030	orde	Homa-	-	0.0	-	0.03	-0.03	-0.08	0.01	0.1	0.
Acidaminococcaceae.id.2166	famil	Homa-	-	-	0.0	-	-0.09	0.04	-0.04	-	0.

© 2021 Chen Z et al. *JAMA Network Open.*

Alcaligenaceae.id.2875	famil	Homa-	0.0	0.0	0.0	0.03	0.01	-0.04	0.06	0.7	0.
Bacteroidaceae.id.917	famil	Homa-	-	0.0	-	0.03	-0.05	-0.09	0.00	0.0	0.
Bifidobacteriaceae.id.433	famil [illegible]	Homa- [illegible]	0.0 [illegible]	0.0 [illegible]	- [illegible]	0.04	0.00	-0.05	0.05	0.9 [illegible]	0. [illegible]

© 2021 Chen Z et al. *JAMA Network Open.*

Christensenellaceae.id.1866	family	Homa-IR	-0.17	0.03	-0.14	0.03	-0.16	-0.21	-0.11	0.00	0.00
Clostridiaceae1.id.1869	family	Homa-IR	-0.04	0.03	-0.04	0.04	-0.04	-0.09	0.01	0.09	0.00
ClostridialesvadinBB60group.id.11286	family	Homa-IR	-0.16	0.03	-0.08	0.03	-0.12	-0.17	-0.08	0.00	0.70
Coriobacteriaceae.id.811	family	Homa-IR	-0.01	0.03	-0.01	0.03	-0.01	-0.06	0.03	0.62	0.00
Desulfovibrionaceae.id.3169	family	Homa-IR	-0.07	0.03	-0.02	0.03	-0.04	-0.09	0.00	0.05	0.00
Enterobacteriaceae.id.3469	family	Homa-IR	-0.03	0.03	0.01	0.03	-0.01	-0.06	0.03	0.58	0.00
Enterococcaceae.id.1828	family	Homa-IR	0.08	0.03	0.02	0.03	0.05	0.01	0.10	0.02	0.52
Erysipelotrichaceae.id.2149	family	Homa-IR	0.07	0.03	0.07	0.03	0.07	0.03	0.12	0.00	0.00
FamilyXIII.id.1957	family	Homa-IR	-0.11	0.03	-0.03	0.03	-0.07	-0.12	-0.03	0.00	0.63
Lachnospiraceae.id.1987	family	Homa-IR	0.07	0.03	0.08	0.03	0.08	0.03	0.12	0.00	0.00
Lactobacillaceae.id.1836	family	Homa-IR	0.03	0.03	0.01	0.03	0.02	-0.02	0.07	0.33	0.00
Pasteurellaceae.id.3689	family	Homa-IR	-0.01	0.03	-0.06	0.03	-0.03	-0.08	0.01	0.16	0.18
Peptococcaceae.id.2024	family	Homa-IR	-0.05	0.03	0.03	0.03	-0.01	-0.06	0.03	0.55	0.71
Peptostreptococcaceae.id.2042	family	Homa-IR	-0.03	0.03	0.01	0.04	-0.01	-0.06	0.03	0.55	0.00
Porphyromonadaceae.id.943	family	Homa-IR	-0.10	0.03	-0.08	0.03	-0.09	-0.14	-0.05	0.00	0.00
Prevotellaceae.id.960	family	Homa-IR	0.00	0.03	0.03	0.03	0.01	-0.03	0.06	0.55	0.00
Rhodospirillaceae.id.2717	family	Homa-IR	-0.13	0.03	-0.03	0.03	-0.08	-0.13	-0.04	0.00	0.79
Rikenellaceae.id.967	family	Homa-IR	-0.13	0.03	-0.10	0.03	-0.12	-0.16	-0.07	0.00	0.00
Ruminococcaceae.id.2050	family	Homa-IR	-0.12	0.03	0.02	0.03	-0.06	-0.10	-0.01	0.01	0.89
Streptococcaceae.id.1850	family	Homa-IR	0.10	0.03	0.06	0.03	0.08	0.03	0.13	0.00	0.00
Succinivibrionaceae.id.3326	family	Homa-IR	-0.03	0.03	0.04	0.03	0.01	-0.04	0.05	0.82	0.51
Veillonellaceae.id.2172	family	Homa-IR	0.08	0.03	-0.02	0.03	0.04	-0.01	0.08	0.11	0.79
Verrucomicrobiaceae.id.4036	family	Homa-IR	-0.13	0.03	-0.03	0.03	-0.08	-0.13	-0.04	0.00	0.77
unknownfamily.id.1000001214	family	Homa-IR	-0.12	0.03	-0.10	0.03	-0.11	-0.15	-0.06	0.00	0.00
unknownfamily.id.1000005471	family	Homa-IR	0.01	0.03	-0.04	0.03	-0.01	-0.06	0.03	0.58	0.37
unknownfamily.id.987	family	Homa-IR	-0.01	0.03	-0.06	0.03	-0.03	-0.08	0.01	0.16	0.00
Bacteroidespectinophilusgroup.id.14371	genus	Homa-IR	-0.06	0.03	-0.03	0.03	-0.05	-0.09	0.00	0.03	0.00
Eubacteriumcoprostanoligenesgroup.id.11375	genus	Homa-IR	-0.03	0.03	0.01	0.03	-0.01	-0.06	0.04	0.68	0.00
Eubacteriumeligensgroup.id.14372	genus	Homa-IR	-0.13	0.03	-0.02	0.03	-0.08	-0.13	-0.04	0.00	0.81
Eubacteriumhalliigroup.id.11338	genus	Homa-IR	0.09	0.03	0.04	0.03	0.07	0.02	0.12	0.00	0.16
Eubacteriumrectalegroup.id.14374	genus	Homa-IR	0.08	0.03	0.06	0.03	0.07	0.02	0.12	0.00	0.00
Eubacteriumruminantiumgroup.id.11340	genus	Homa-IR	-0.07	0.03	-0.05	0.03	-0.06	-0.11	-0.02	0.01	0.00
Eubacteriumventriosumgroup.id.11341	genus	Homa-IR	0.03	0.03	0.07	0.03	0.05	0.00	0.10	0.04	0.00
Eubacteriumxylanophilumgroup.id.14375	genus	Homa-IR	-0.13	0.03	-0.03	0.03	-0.08	-0.13	-0.03	0.00	0.74
Ruminococcusgauvreauiigroup.id.11342	genus	Homa-IR	0.02	0.03	0.10	0.03	0.05	0.01	0.10	0.02	0.65
Ruminococcusgnavusgroup.id.14376	genus	Homa-IR	0.05	0.03	0.09	0.03	0.07	0.02	0.11	0.00	0.00
Ruminococcustorquesgroup.id.14377	genus	Homa-IR	0.07	0.03	0.03	0.03	0.05	0.01	0.10	0.03	0.10
Acidaminococcus.id.2167	genus	Homa-IR	0.06	0.03	0.05	0.03	0.06	0.01	0.10	0.02	0.00
Adlercreutzia.id.812	genus	Homa-IR	0.07	0.03	0.04	0.03	0.06	0.01	0.10	0.02	0.00
Akkermansia.id.4037	genus	Homa-IR	-0.01	0.03	-0.06	0.03	-0.03	-0.08	0.01	0.16	0.00
Alistipes.id.968	genus	Homa-IR	-0.13	0.03	-0.09	0.03	-0.11	-0.16	-0.07	0.00	0.00
Alloprevotella.id.961	genus	Homa-IR	-0.03	0.03	0.04	0.03	0.00	-0.04	0.05	0.87	0.43
Anaerostipes.id.1991	genus	Homa-IR	0.02	0.03	0.03	0.03	0.03	-0.02	0.08	0.21	0.00
Anaerotruncus.id.2054	genus	Homa-IR	-0.12	0.03	-0.04	0.03	-0.09	-0.13	-0.04	0.00	0.67

© 2021 Chen Z et al. *JAMA Network Open.*

Bacteroides.id.918	genus	Homa-IR	-0.06	0.03	-0.04	0.03	-0.05	-0.09	0.00	0.05	0.00
Barnesiella.id.944	genus	Homa-IR	-0.08	0.03	-0.04	0.03	-0.06	-0.11	-0.01	0.01	0.06
Bifidobacterium.id.436	genus	Homa-IR	0.01	0.03	0.00	0.04	0.00	-0.04	0.05	0.85	0.00
Blautia.id.1992	genus	Homa-IR	0.02	0.01	0.00	0.03	0.00	-0.03	0.03	0.75	0.00

© 2021 Chen Z et al. *JAMA Network Open.*

Butyricicoccus.id.2055	genus	Homa-IR	0.05	0.03	0.01	0.03	0.03	-0.01	0.08	0.15	0.00
Butyricimonas.id.945	genus	Homa-IR	-0.09	0.03	-0.08	0.03	-0.08	-0.13	-0.04	0.00	0.00
Butyrivibrio.id.1993	genus	Homa-IR	-0.09	0.03	-0.03	0.03	-0.06	-0.11	-0.02	0.01	0.46
Catenibacterium.id.2153	genus	Homa-IR	0.05	0.03	0.02	0.03	0.03	-0.01	0.08	0.16	0.00
ChristensenellaceaeR7group.id.11283	genus	Homa-IR	-0.17	0.03	-0.13	0.03	-0.15	-0.20	-0.11	0.00	0.00
Clostridiumsensustricto1.id.1873	genus	Homa-IR	-0.04	0.03	-0.05	0.04	-0.04	-0.09	0.01	0.08	0.00
Collinsella.id.815	genus	Homa-IR	0.05	0.03	0.00	0.03	0.02	-0.02	0.07	0.34	0.07
Coprococcus1.id.11301	genus	Homa-IR	-0.08	0.03	-0.04	0.03	-0.06	-0.11	-0.02	0.01	0.00
Coprococcus2.id.11302	genus	Homa-IR	-0.08	0.03	-0.03	0.03	-0.06	-0.10	-0.01	0.01	0.00
Coprococcus3.id.11303	genus	Homa-IR	0.04	0.03	0.03	0.03	0.04	-0.01	0.08	0.11	0.00
Desulfovibrio.id.3173	genus	Homa-IR	-0.06	0.03	-0.02	0.03	-0.04	-0.09	0.00	0.06	0.00
Dialister.id.2183	genus	Homa-IR	0.06	0.03	-0.03	0.03	0.02	-0.03	0.06	0.48	0.70
Dorea.id.1997	genus	Homa-IR	0.07	0.03	0.00	0.03	0.04	-0.01	0.08	0.13	0.57
Eggerthella.id.819	genus	Homa-IR	0.00	0.03	0.07	0.03	0.03	-0.01	0.08	0.17	0.63
Eisenbergiella.id.11304	genus	Homa-IR	-0.05	0.03	0.01	0.03	-0.02	-0.07	0.02	0.32	0.40
Enterococcus.id.1831	genus	Homa-IR	0.08	0.03	0.02	0.03	0.05	0.01	0.10	0.02	0.40
Enterorhabdus.id.820	genus	Homa-IR	-0.07	0.03	-0.05	0.03	-0.06	-0.11	-0.01	0.01	0.00
Erysipelatoclostridium.id.11381	genus	Homa-IR	-0.02	0.03	0.05	0.03	0.01	-0.03	0.06	0.55	0.54
ErysipelotrichaceaeUCG003.id.11384	genus	Homa-IR	0.04	0.03	0.04	0.03	0.04	-0.01	0.09	0.10	0.00
Faecalibacterium.id.2057	genus	Homa-IR	-0.02	0.03	0.07	0.03	0.02	-0.03	0.06	0.47	0.72
FamilyXIIIAD3011group.id.11293	genus	Homa-IR	-0.12	0.03	-0.10	0.03	-0.11	-0.16	-0.07	0.00	0.00
FamilyXIIIUCG001.id.11294	genus	Homa-IR	-0.04	0.03	-0.04	0.03	-0.04	-0.08	0.01	0.11	0.00
Fusicatenibacter.id.11305	genus	Homa-IR	0.07	0.03	0.05	0.03	0.06	0.02	0.11	0.01	0.00
Haemophilus.id.3698	genus	Homa-IR	-0.01	0.03	-0.05	0.03	-0.03	-0.08	0.01	0.19	0.00
Holdemanella.id.11393	genus	Homa-IR	0.07	0.03	0.07	0.03	0.07	0.02	0.11	0.00	0.00
Intestinibacter.id.11345	genus	Homa-IR	0.00	0.03	0.03	0.04	0.02	-0.03	0.06	0.51	0.00
Intestinimonas.id.2062	genus	Homa-IR	-0.10	0.03	-0.03	0.03	-0.07	-0.12	-0.03	0.00	0.52
Lachnoclostridium.id.11308	genus	Homa-IR	0.03	0.03	0.07	0.03	0.05	0.00	0.09	0.05	0.00
Lachnospira.id.2004	genus	Homa-IR	-0.01	0.03	0.03	0.03	0.01	-0.04	0.06	0.69	0.00
LachnospiraceaeFCS020group.id.11314	genus	Homa-IR	-0.03	0.03	-0.08	0.03	-0.05	-0.10	-0.01	0.02	0.19
LachnospiraceaeND3007group.id.11317	genus	Homa-IR	-0.06	0.03	0.00	0.03	-0.03	-0.08	0.01	0.18	0.52
LachnospiraceaeNK4A136group.id.11319	genus	Homa-IR	-0.07	0.03	0.02	0.03	-0.03	-0.07	0.02	0.23	0.72
LachnospiraceaeUCG001.id.11321	genus	Homa-IR	0.01	0.03	0.01	0.03	0.01	-0.04	0.06	0.68	0.00
LachnospiraceaeUCG004.id.11324	genus	Homa-IR	-0.03	0.03	-0.04	0.03	-0.04	-0.08	0.01	0.12	0.00
LachnospiraceaeUCG010.id.11330	genus	Homa-IR	-0.03	0.03	-0.05	0.03	-0.04	-0.09	0.01	0.09	0.00
Lactobacillus.id.1837	genus	Homa-IR	0.04	0.03	0.02	0.03	0.03	-0.01	0.08	0.18	0.00
Marvinbryantia.id.2005	genus	Homa-IR	-0.12	0.03	-0.08	0.03	-0.10	-0.15	-0.06	0.00	0.00
Megamonas.id.2184	genus	Homa-IR	0.04	0.03	0.00	0.03	0.02	-0.02	0.07	0.31	0.00
Megasphaera.id.2185	genus	Homa-IR	0.04	0.03	0.06	0.03	0.05	0.00	0.09	0.04	0.00
Mitsuokella.id.2186	genus	Homa-IR	0.08	0.03	0.01	0.03	0.05	0.00	0.09	0.04	0.58
Odoribacter.id.952	genus	Homa-IR	-0.14	0.03	-0.08	0.03	-0.11	-0.16	-0.06	0.00	0.35
Olsenella.id.822	genus	Homa-IR	0.02	0.03	-0.05	0.03	-0.01	-0.06	0.03	0.55	0.45
Oscillospira.id.2064	genus	Homa-IR	-0.07	0.03	-0.05	0.03	-0.06	-0.11	-0.01	0.01	0.00
Parabacteroides.id.954	genus	Homa-IR	-0.07	0.03	-0.07	0.03	-0.07	-0.12	-0.02	0.00	0.00
Paraprevotella.id.962	genus	Homa-IR	-0.03	0.03	-0.04	0.03	-0.03	-0.08	0.01	0.14	0.00

© 2021 Chen Z et al. *JAMA Network Open.*

Parasutterella.id.2892	genus	Homa-IR	0.01	0.03	0.06	0.03	0.03	-0.01	0.08	0.16	0.00

© 2021 Chen Z et al. *JAMA Network Open.*

Peptococcus.id.2037	genus	Homa-IR	-0.01	0.03	0.04	0.03	0.01	-0.04	0.06	0.65	0.18
Phascolarctobacterium.id.2168	genus	Homa-IR	-0.07	0.03	-0.04	0.03	-0.05	-0.10	-0.01	0.03	0.00
Prevotella2.id.11180	genus	Homa-IR	0.01	0.03	-0.02	0.03	-0.01	-0.05	0.04	0.82	0.00
Prevotella7.id.11182	genus	Homa-IR	0.00	0.03	0.03	0.03	0.02	-0.03	0.06	0.52	0.00
Prevotella9.id.11183	genus	Homa-IR	0.02	0.03	0.02	0.03	0.02	-0.02	0.07	0.32	0.00
PrevotellaceaeNK3B31group.id.11185	genus	Homa-IR	0.00	0.03	0.03	0.03	0.02	-0.03	0.06	0.46	0.00
PrevotellaceaeUCG001.id.11186	genus	Homa-IR	-0.02	0.03	0.02	0.03	0.00	-0.04	0.05	0.99	0.00
RikenellaceaeRC9gutgroup.id.11191	genus	Homa-IR	0.00	0.03	-0.04	0.03	-0.02	-0.07	0.03	0.40	0.00
Romboutsia.id.11347	genus	Homa-IR	-0.05	0.03	0.00	0.03	-0.03	-0.07	0.02	0.26	0.00
Roseburia.id.2012	genus	Homa-IR	0.05	0.03	0.05	0.03	0.05	0.00	0.10	0.03	0.00
Ruminiclostridium5.id.11355	genus	Homa-IR	-0.10	0.03	-0.01	0.03	-0.06	-0.10	-0.01	0.02	0.71
Ruminiclostridium6.id.11356	genus	Homa-IR	-0.16	0.03	-0.02	0.03	-0.09	-0.14	-0.05	0.00	0.89
Ruminiclostridium9.id.11357	genus	Homa-IR	-0.09	0.03	-0.14	0.03	-0.11	-0.16	-0.06	0.00	0.04
RuminococcaceaeNK4A214group.id.11358	genus	Homa-IR	-0.18	0.03	-0.15	0.03	-0.16	-0.21	-0.12	0.00	0.00
RuminococcaceaeUCG002.id.11360	genus	Homa-IR	-0.13	0.03	-0.13	0.03	-0.13	-0.17	-0.08	0.00	0.00
RuminococcaceaeUCG003.id.11361	genus	Homa-IR	-0.08	0.03	-0.10	0.03	-0.09	-0.14	-0.04	0.00	0.00
RuminococcaceaeUCG004.id.11362	genus	Homa-IR	0.01	0.03	-0.01	0.03	0.00	-0.04	0.05	0.93	0.00
RuminococcaceaeUCG005.id.11363	genus	Homa-IR	-0.20	0.03	-0.11	0.03	-0.16	-0.20	-0.11	0.00	0.74
RuminococcaceaeUCG008.id.11365	genus	Homa-IR	-0.13	0.03	-0.06	0.03	-0.10	-0.14	-0.05	0.00	0.64
RuminococcaceaeUCG010.id.11367	genus	Homa-IR	-0.19	0.03	-0.13	0.03	-0.17	-0.21	-0.12	0.00	0.47
RuminococcaceaeUCG013.id.11370	genus	Homa-IR	-0.01	0.03	0.08	0.03	0.04	-0.01	0.08	0.13	0.70
RuminococcaceaeUCG014.id.11371	genus	Homa-IR	-0.14	0.03	-0.09	0.03	-0.12	-0.16	-0.07	0.00	0.28
Ruminococcus1.id.11373	genus	Homa-IR	-0.11	0.03	0.06	0.03	-0.03	-0.08	0.02	0.19	0.92
Ruminococcus2.id.11374	genus	Homa-IR	-0.02	0.03	0.05	0.03	0.01	-0.03	0.06	0.52	0.55
Sellimonas.id.14369	genus	Homa-IR	0.03	0.03	0.05	0.03	0.04	-0.01	0.09	0.09	0.00
Senegalimassilia.id.11160	genus	Homa-IR	-0.01	0.03	-0.05	0.03	-0.03	-0.08	0.01	0.17	0.00
Slackia.id.825	genus	Homa-IR	0.05	0.03	-0.06	0.03	0.00	-0.05	0.04	0.95	0.81
Streptococcus.id.1853	genus	Homa-IR	0.10	0.03	0.06	0.03	0.08	0.04	0.13	0.00	0.00
Subdoligranulum.id.2070	genus	Homa-IR	0.01	0.03	0.02	0.03	0.01	-0.03	0.06	0.58	0.00
Succiniclasticum.id.2169	genus	Homa-IR	-0.03	0.03	0.00	0.03	-0.02	-0.06	0.03	0.52	0.00
Sutterella.id.2896	genus	Homa-IR	-0.02	0.03	-0.05	0.03	-0.04	-0.08	0.01	0.14	0.00
Terrisporobacter.id.11348	genus	Homa-IR	-0.04	0.03	-0.02	0.04	-0.03	-0.07	0.02	0.22	0.00
Turicibacter.id.2162	genus	Homa-IR	-0.05	0.03	-0.05	0.03	-0.05	-0.09	0.00	0.04	0.00
Tyzzerella3.id.11335	genus	Homa-IR	0.08	0.03	0.02	0.03	0.05	0.01	0.10	0.02	0.42
Veillonella.id.2198	genus	Homa-IR	0.01	0.03	-0.04	0.03	-0.01	-0.06	0.03	0.63	0.00
unknowngenus.id.1000000073	genus	Homa-IR	-0.16	0.03	-0.08	0.03	-0.12	-0.17	-0.08	0.00	0.70
unknowngenus.id.1000001215	genus	Homa-IR	-0.13	0.03	-0.03	0.03	-0.08	-0.13	-0.04	0.00	0.77
unknowngenus.id.1000005472	genus	Homa-IR	-0.12	0.03	-0.10	0.03	-0.11	-0.15	-0.06	0.00	0.00
unknowngenus.id.1000005479	genus	Homa-IR	-0.05	0.03	-0.05	0.03	-0.05	-0.10	0.00	0.03	0.00
unknowngenus.id.1000013899	genus	Homa-IR	0.01	0.03	-0.04	0.03	-0.01	-0.06	0.03	0.58	0.37
unknowngenus.id.2071	genus	Homa-IR	-0.17	0.03	-0.06	0.03	-0.12	-0.16	-0.07	0.00	0.82
unknowngenus.id.2755	genus	Homa-IR	-0.13	0.03	-0.03	0.03	-0.08	-0.13	-0.04	0.00	0.79
unknowngenus.id.826	genus	Homa-IR	-0.05	0.03	-0.01	0.04	-0.03	-0.07	0.02	0.25	0.00
unknowngenus.id.964	genus	Homa-IR	0.04	0.03	0.02	0.03	0.03	-0.02	0.08	0.20	0.00

© 2021 Chen Z et al. *JAMA Network Open.*

Taxa in RS only	Clade	Phenotype	Beta_RS	Se_RS
BacteroidalesS247group.id.11173	family	Homa-IR	-0.05	0.03
Veillonellaceae.id.2172	family	Homa-IR	0.08	0.03
Bacteroidespectinophilusgroup.id.14371	genus	Homa-IR	-0.06	0.03
Enterobacter.id.3502	genus	Homa-IR	0.08	0.04
EscherichiaShigella.id.3504	genus	Homa-IR	-0.05	0.03
Eubacteriumcoprostanoligenesgroup.id.11375	genus	Homa-IR	-0.03	0.03
Eubacteriumeligensgroup.id.14372	genus	Homa-IR	-0.13	0.03
Eubacteriumhalliigroup.id.11338	genus	Homa-IR	0.09	0.03
Eubacteriumoxidoreducensgroup.id.11339	genus	Homa-IR	-0.18	0.03
Eubacteriumxylanophilumgroup.id.14375	genus	Homa-IR	-0.13	0.03
Hungatella.id.11306	genus	Homa-IR	-0.02	0.03
Klebsiella.id.3507	genus	Homa-IR	0.05	0.03
LachnospiraceaeNC2004group.id.11316	genus	Homa-IR	-0.13	0.03
LachnospiraceaeUCG008.id.11328	genus	Homa-IR	-0.05	0.03
unknowngenus.id.1868	genus	Homa-IR	-0.14	0.03

Taxa in LLD only	Clade	Phenotype	Beta_RS	Se_RS
Archaea.id.2	domain	Homa-IR	-0.14	0.03
Euryarchaeota.id.55	phylum	Homa-IR	-0.13	0.03
Lentisphaerae.id.2238	phylum	Homa-IR	-0.05	0.03
Actinomycetales.id.420	order	Homa-IR	0.05	0.03
Mycoplasmatales.id.3946	order	Homa-IR	-0.03	0.03
NB1.n.id.3953	order	Homa-IR	-0.04	0.03
Pseudomonadales.id.3709	order	Homa-IR	-0.02	0.03
Spirochaetales.id.3870	order	Homa-IR	-0.02	0.03
Synergistales.id.3900	order	Homa-IR	-0.06	0.03
Thermoplasmatales.id.220	order	Homa-IR	-0.03	0.03
unknownorder.id.1000000003	order	Homa-IR	0.01	0.03
Verrucomicrobiales.id.4030	order	Homa-IR	-0.06	0.03
Victivallales.id.2254	order	Homa-IR	-0.05	0.03
Xanthomonadales.id.3786	order	Homa-IR	-0.02	0.03
Spirochaetes.id.3856	class	Homa-IR	-0.02	0.03
Synergistia.id.3899	class	Homa-IR	-0.06	0.03
Thermoplasmata.id.210	class	Homa-IR	-0.03	0.03
Actinomycetaceae.id.421	family	Homa-IR	0.05	0.03
BacteroidalesS24.7group.id.11173	family	Homa-IR	-0.05	0.03
Christensenellaceae.id.1866	family	Homa-IR	-0.14	0.03
FamilyXI.id.1936	family	Homa-IR	0.00	0.03
unknownfamily.id.1000000004	family	Homa-IR	0.01	0.03
unknownfamily.id.1000006161	family	Homa-IR	-0.04	0.03
unknownfamily.id.1855	family	Homa-IR	-0.01	0.03
vadinBE97.id.14446	family	Homa-IR	-0.04	0.03
Veillonellaceae.id.2172	family	Homa-IR	-0.02	0.03

© 2021 Chen Z et al. *JAMA Network Open.*

Xanthomonadaceae.id.3799	family	Homa-IR	-0.02	0.03
Clostridiuminnocuumgroup.id.14397	genus	Homa-IR	0.01	0.03
Eubacteriumbrachygroup.id.11296	genus	Homa-IR	0.06	0.03

© 2021 Chen Z et al. *JAMA Network Open.*

Abiotrophia.id.1803	genus	Homa-IR	0.01	0.03
Actinomyces.id.423	genus	Homa-IR	0.06	0.03
Actinotignum.id.11137	genus	Homa-IR	-0.04	0.03
Allisonella.id.2174	genus	Homa-IR	0.09	0.03
Arcanobacterium.id.424	genus	Homa-IR	-0.01	0.03
Asaccharobacter.id.813	genus	Homa-IR	0.04	0.03
Bilophila.id.3170	genus	Homa-IR	-0.02	0.03
CandidatusMethanogranum.id.11110	genus	Homa-IR	-0.01	0.03
Catenisphaera.id.14395	genus	Homa-IR	0.00	0.03
Cellulosilyticum.id.1995	genus	Homa-IR	-0.06	0.03
Cetobacterium.id.2209	genus	Homa-IR	0.07	0.03
Cloacibacillus.id.3908	genus	Homa-IR	-0.05	0.03
Coprobacillus.id.2154	genus	Homa-IR	0.02	0.03
CoriobacteriaceaeUCG.002.id.11158	genus	Homa-IR	-0.04	0.03
CoriobacteriaceaeUCG.003.id.11159	genus	Homa-IR	0.03	0.03
Denitrobacterium.id.818	genus	Homa-IR	-0.02	0.03
dgA.11gutgroup.id.978	genus	Homa-IR	0.02	0.03
Dielma.id.11380	genus	Homa-IR	-0.01	0.03
Epulopiscium.id.1998	genus	Homa-IR	0.03	0.03
ErysipelotrichaceaeUCG006.id.11386	genus	Homa-IR	0.01	0.03
ErysipelotrichaceaeUCG009.id.11389	genus	Homa-IR	0.04	0.03
Escherichia.Shigella.id.3504	genus	Homa-IR	0.00	0.03
Eubacterium.id.1932	genus	Homa-IR	0.05	0.03
Faecalicoccus.id.11391	genus	Homa-IR	0.01	0.03
Faecalitalea.id.11392	genus	Homa-IR	0.05	0.03
Flavonifractor.id.2059	genus	Homa-IR	0.00	0.03
Gardnerella.id.437	genus	Homa-IR	0.01	0.03
Gordonibacter.id.821	genus	Homa-IR	0.00	0.03
Hafnia.Obesumbacterium.id.14636	genus	Homa-IR	0.01	0.03
Howardella.id.2000	genus	Homa-IR	0.02	0.03
LachnospiraceaeNK3A20group.id.11318	genus	Homa-IR	0.00	0.03
Lactococcus.id.1851	genus	Homa-IR	-0.02	0.03
Lautropia.id.2905	genus	Homa-IR	0.01	0.03
Leuconostoc.id.1841	genus	Homa-IR	0.00	0.03
Methanobrevibacter.id.123	genus	Homa-IR	-0.13	0.03
Methanosphaera.id.124	genus	Homa-IR	-0.07	0.03
Mogibacterium.id.1960	genus	Homa-IR	-0.01	0.03
Morganella.id.3512	genus	Homa-IR	0.05	0.03
Porphyromonas.id.956	genus	Homa-IR	-0.02	0.03
Prevotella1.id.11179	genus	Homa-IR	0.02	0.03
PrevotellaceaeUCG003.id.11187	genus	Homa-IR	0.07	0.03
PrevotellaceaeUCG004.id.11188	genus	Homa-IR	-0.05	0.03
Pseudomonas.id.3723	genus	Homa-IR	-0.02	0.03
Rikenella.id.973	genus	Homa-IR	0.01	0.03

© 2021 Chen Z et al. *JAMA Network Open.*

RuminococcaceaeNK4A214group.id.11358	genus	Homa-IR	-0.15	0.03
RuminococcaceaeUCG005.id.11363	genus	Homa-IR	-0.11	0.03
RuminococcaceaeUCG008.id.11365	genus	Homa-IR	-0.06	0.03

© 2021 Chen Z et al. *JAMA Network Open.*

RuminococcaceaeUCG010.id.11367	genus	Homa-IR	-0.13	0.03
Sarcina.id.1896	genus	Homa-IR	0.05	0.03
Solobacterium.id.2161	genus	Homa-IR	0.06	0.03
Staphylococcus.id.1780	genus	Homa-IR	0.04	0.03
Stenotrophomonas.id.3818	genus	Homa-IR	-0.02	0.03
Succinivibrio.id.3331	genus	Homa-IR	0.04	0.03
Synergistes.id.3913	genus	Homa-IR	-0.03	0.03
Syntrophococcus.id.2016	genus	Homa-IR	0.00	0.03
Treponema2.id.11577	genus	Homa-IR	-0.02	0.03
Tyzzerella4.id.11336	genus	Homa-IR	0.05	0.03
unknowngenus.id.1000000005	genus	Homa-IR	0.01	0.03
unknowngenus.id.1000027565	genus	Homa-IR	-0.04	0.03
unknowngenus.id.1000043796	genus	Homa-IR	-0.01	0.03
unknowngenus.id.1854	genus	Homa-IR	-0.03	0.03
unknowngenus.id.2163	genus	Homa-IR	-0.05	0.03
unknowngenus.id.3332	genus	Homa-IR	0.05	0.03
unknowngenus.id.441	genus	Homa-IR	-0.09	0.03
Ureaplasma.id.3951	genus	Homa-IR	-0.03	0.03
Varibaculum.id.427	genus	Homa-IR	0.01	0.03
Veillonella.id.2198	genus	Homa-IR	-0.04	0.03
Weissella.id.1843	genus	Homa-IR	0.01	0.03

© 2021 Chen Z et al. *JAMA Network Open.*

Supplementary Table 2 - Associations of taxa and insulin resistance

Effect estimates in RS and LLD were calculated using linear regression; pooled estimates were calculated based on a mixed-effect meta-analysis.

Model 3: Adjusted for age, sex, Time in mail (RS), Batch (RS), smoking, education level (RS), physical activity, alcohol intake, total energy intake, and BMI.

Abbreviation, RS, Rotterdam Study, LLD, Lifelines-Deep Study. P<0.0005 indicates statistical significance

Overlapping taxa in RS and LLD	Clade	Phenotype	Beta_RS	Se_RS	Beta_LLD	Se_LLD	Beta_Meta	Lower95%CI_Meta	Upper95%CI_Meta	p_Meta	I^2
Bacteria.id.3	domain	Homa-IR	0.00	0.03	0.07	0.03	0.04	0.00	0.08	0.03	0.554
Actinobacteria.id.400	phylum	Homa-IR	0.02	0.03	0.00	0.03	0.01	-0.03	0.05	0.54	0.069
Bacteroidetes.id.905	phylum	Homa-IR	-0.04	0.03	-0.02	0.03	-0.03	-0.07	0.01	0.11	0
Cyanobacteria.id.1500	phylum	Homa-IR	-0.09	0.03	0.02	0.03	-0.04	-0.08	0.00	0.07	0.778
Firmicutes.id.1672	phylum	Homa-IR	0.00	0.03	0.04	0.03	0.02	-0.02	0.06	0.28	0.702
Proteobacteria.id.2375	phylum	Homa-IR	0.00	0.03	-0.01	0.03	0.00	-0.04	0.04	0.88	0
Tenericutes.id.3919	phylum	Homa-IR	-0.08	0.03	-0.06	0.03	-0.07	-0.11	-0.03	0.00	0
Verrucomicrobia.id.3982	phylum	Homa-IR	0.02	0.03	-0.01	0.03	0.01	-0.03	0.05	0.79	0
Actinobacteria.id.419	class	Homa-IR	0.02	0.03	0.01	0.03	0.02	-0.03	0.06	0.46	0.06
Alphaproteobacteria.id.2379	class	Homa-IR	-0.08	0.03	0.01	0.03	-0.04	-0.08	0.00	0.05	0.394
Bacilli.id.1673	class	Homa-IR	0.03	0.03	0.03	0.03	0.03	-0.01	0.07	0.12	0
Bacteroidia.id.912	class	Homa-IR	-0.04	0.03	-0.02	0.03	-0.03	-0.07	0.01	0.12	0
Betaproteobacteria.id.2867	class	Homa-IR	-0.01	0.03	0.01	0.03	0.00	-0.04	0.04	0.96	0
Clostridia.id.1859	class	Homa-IR	-0.03	0.03	0.02	0.03	-0.01	-0.05	0.04	0.79	0.712
Coriobacteriia.id.809	class	Homa-IR	0.00	0.03	-0.01	0.03	-0.01	-0.05	0.03	0.73	0
Deltaproteobacteria.id.3087	class	Homa-IR	-0.03	0.03	-0.01	0.03	-0.02	-0.06	0.02	0.34	0.151
Erysipelotrichia.id.2147	class	Homa-IR	0.05	0.03	0.04	0.03	0.05	0.00	0.09	0.03	0
Gammaproteobacteria.id.3303	class	Homa-IR	0.00	0.03	-0.01	0.03	0.00	-0.04	0.04	0.83	0
Melainabacteria.id.1589	class	Homa-IR	-0.08	0.03	0.01	0.03	-0.04	-0.08	0.01	0.09	0.677
Mollicutes.id.3920	class	Homa-IR	-0.08	0.03	-0.06	0.03	-0.07	-0.11	-0.03	0.00	0
Negativicutes.id.2164	class	Homa-IR	0.01	0.03	-0.02	0.03	0.00	-0.04	0.04	0.84	0
Verrucomicrobiae.id.4029	class	Homa-IR	0.02	0.03	-0.01	0.03	0.01	-0.03	0.05	0.78	0
Aeromonadales.id.3316	order	Homa-IR	-0.03	0.03	0.01	0.03	-0.01	-0.05	0.03	0.66	0.55
Bacteroidales.id.913	order	Homa-IR	-0.04	0.03	-0.02	0.03	-0.03	-0.07	0.01	0.12	0
Bifidobacteriales.id.432	order	Homa-IR	0.02	0.03	0.01	0.03	0.01	-0.03	0.05	0.59	0
Burkholderiales.id.2874	order	Homa-IR	-0.01	0.03	0.01	0.03	0.00	-0.04	0.04	0.95	0
Clostridiales.id.1863	order	Homa-IR	-0.03	0.03	0.02	0.03	-0.01	-0.05	0.04	0.79	0.714

© 2021 Chen Z et al. *JAMA Network Open.*

Coriobacteriales.id.810	order	Homa-IR	0.00	0.03	-0.01	0.03	-0.01	-0.05	0.03	0.73	0
Desulfovibrionales.id.3156	order	Homa-IR	-0.03	0.03	-0.01	0.03	-0.02	-0.06	0.02	0.35	0.163

© 2021 Chen Z et al. *JAMA Network Open.*

Enterobacteriales.id.3468	order	Homa-IR	0.02	0.03	0.01	0.03	0.01	-0.03	0.05	0.52	0
Erysipelotrichales.id.2148	order	Homa-IR	0.05	0.03	0.04	0.03	0.05	0.00	0.09	0.03	0
Gastranaerophilales.id.1591	order	Homa-IR	-0.08	0.03	0.01	0.03	-0.03	-0.08	0.01	0.09	0.673
Lactobacillales.id.1800	order	Homa-IR	0.03	0.03	0.03	0.03	0.03	-0.01	0.07	0.13	0
MollicutesRF9.id.11579	order	Homa-IR	-0.06	0.03	-0.06	0.03	-0.06	-0.10	-0.02	0.00	0
Pasteurellales.id.3688	order	Homa-IR	-0.02	0.03	-0.05	0.03	-0.03	-0.07	0.01	0.09	0.547
Rhodospirillales.id.2667	order	Homa-IR	-0.08	0.03	0.01	0.03	-0.04	-0.08	0.00	0.05	0.444
Selenomonadales.id.2165	order	Homa-IR	0.01	0.03	-0.02	0.03	0.00	-0.04	0.04	0.84	0
Verrucomicrobiales.id.4030	order	Homa-IR	0.02	0.03	-0.01	0.03	0.01	-0.03	0.05	0.78	0
Acidaminococcaceae.id.2166	family	Homa-IR	-0.05	0.03	-0.02	0.03	-0.04	-0.08	0.00	0.07	0
Alcaligenaceae.id.2875	family	Homa-IR	0.00	0.03	0.02	0.03	0.01	-0.03	0.05	0.66	0
Bacteroidaceae.id.917	family	Homa-IR	-0.04	0.03	-0.01	0.03	-0.03	-0.07	0.02	0.22	0
Bifidobacteriaceae.id.433	family	Homa-IR	0.02	0.03	0.01	0.03	0.01	-0.03	0.05	0.59	0
Christensenellaceae.id.1866	family	Homa-IR	-0.10	0.03	-0.07	0.03	-0.08	-0.12	-0.04	0.00	0
Clostridiaceae1.id.1869	family	Homa-IR	-0.03	0.03	-0.01	0.03	-0.02	-0.06	0.02	0.35	0
ClostridialesvadinBB60group.id.11286	family	Homa-IR	-0.11	0.03	-0.03	0.03	-0.07	-0.11	-0.03	0.00	0.613
Coriobacteriaceae.id.811	family	Homa-IR	0.00	0.03	-0.01	0.03	-0.01	-0.05	0.03	0.73	0
Desulfovibrionaceae.id.3169	family	Homa-IR	-0.03	0.03	-0.01	0.03	-0.02	-0.06	0.02	0.34	0.135
Enterobacteriaceae.id.3469	family	Homa-IR	0.02	0.03	0.01	0.03	0.01	-0.03	0.05	0.52	0
Enterococcaceae.id.1828	family	Homa-IR	0.05	0.03	-0.03	0.03	0.01	-0.02	0.05	0.45	0.068
Erysipelotrichaceae.id.2149	family	Homa-IR	0.05	0.03	0.04	0.03	0.05	0.00	0.09	0.03	0
FamilyXIII.id.1957	family	Homa-IR	-0.03	0.03	-0.02	0.03	-0.03	-0.07	0.01	0.17	0.699
Lachnospiraceae.id.1987	family	Homa-IR	0.04	0.03	0.03	0.03	0.04	0.00	0.08	0.07	0
Lactobacillaceae.id.1836	family	Homa-IR	-0.02	0.03	-0.04	0.03	-0.03	-0.07	0.01	0.11	0
Pasteurellaceae.id.3689	family	Homa-IR	-0.02	0.03	-0.05	0.03	-0.03	-0.07	0.01	0.09	0.547
Peptococcaceae.id.2024	family	Homa-IR	-0.03	0.03	-0.01	0.03	-0.02	-0.06	0.02	0.33	0.629
Peptostreptococcaceae.id.2042	family	Homa-IR	-0.04	0.03	0.00	0.03	-0.02	-0.06	0.02	0.30	0
Porphyromonadaceae.id.943	family	Homa-IR	-0.06	0.03	-0.05	0.03	-0.05	-0.09	-0.01	0.01	0
Prevotellaceae.id.960	family	Homa-IR	-0.02	0.03	0.02	0.03	0.00	-0.04	0.04	0.95	0.181
Rhodospirillaceae.id.2717	family	Homa-IR	-0.08	0.03	0.01	0.03	-0.04	-0.08	0.00	0.06	0.446
Rikenellaceae.id.967	family	Homa-IR	-0.07	0.03	-0.04	0.03	-0.05	-0.10	-0.01	0.01	0
Ruminococcaceae.id.2050	family	Homa-IR	-0.09	0.03	0.00	0.03	-0.05	-0.09	-0.01	0.02	0.851

© 2021 Chen Z et al. *JAMA Network Open.*

Streptococcaceae.id.1850	family	Homa-IR	0.04	0.03	0.06	0.03	0.05	0.01	0.09	0.02	0
Succinivibrionaceae.id.3326	family	Homa-IR	-0.03	0.03	0.01	0.03	-0.01	-0.05	0.03	0.64	0.558

© 2021 Chen Z et al. *JAMA Network Open.*

Veillonellaceae.id.2172	family	Homa-IR	0.06	0.03	-0.02	0.03	0.02	-0.02	0.07	0.22	0.71
Verrucomicrobiaceae.id.4036	family	Homa-IR	0.02	0.03	-0.01	0.03	0.01	-0.03	0.05	0.78	0
unknownfamily.id.1000001214	family	Homa-IR	-0.08	0.03	0.01	0.03	-0.03	-0.08	0.01	0.09	0.673
unknownfamily.id.1000005471	family	Homa-IR	-0.06	0.03	-0.06	0.03	-0.06	-0.10	-0.02	0.00	0
unknownfamily.id.987	family	Homa-IR	0.01	0.03	-0.06	0.03	-0.02	-0.06	0.02	0.31	0
Bacteroidespectinophilusgroup.id.14371	genus	Homa-IR	-0.02	0.03	-0.01	0.03	-0.02	-0.06	0.02	0.36	0
Eubacteriumcoprostanoligenesgroup.id.11375	genus	Homa-IR	-0.02	0.03	-0.02	0.03	-0.02	-0.06	0.02	0.38	0.082
Eubacteriumeligensgroup.id.14372	genus	Homa-IR	-0.09	0.03	-0.05	0.03	-0.07	-0.11	-0.03	0.00	0.658
Eubacteriumhalliigroup.id.11338	genus	Homa-IR	0.07	0.03	0.02	0.03	0.05	0.00	0.09	0.03	0.294
Eubacteriumrectalegroup.id.14374	genus	Homa-IR	0.01	0.03	0.03	0.03	0.02	-0.02	0.06	0.33	0
Eubacteriumruminantiumgroup.id.11340	genus	Homa-IR	-0.04	0.03	-0.03	0.03	-0.04	-0.07	0.00	0.08	0
Eubacteriumventriosumgroup.id.11341	genus	Homa-IR	0.03	0.03	0.03	0.03	0.03	-0.01	0.07	0.14	0.269
Eubacteriumxylanophilumgroup.id.14375	genus	Homa-IR	-0.07	0.03	-0.03	0.03	-0.05	-0.09	-0.01	0.01	0.716
Ruminococcusgauvreauiigroup.id.11342	genus	Homa-IR	0.00	0.03	0.01	0.03	0.00	-0.04	0.04	0.97	0.683
Ruminococcusgnavusgroup.id.14376	genus	Homa-IR	0.02	0.03	0.07	0.03	0.05	0.01	0.09	0.02	0.492
Ruminococcustorquesgroup.id.14377	genus	Homa-IR	0.06	0.03	0.00	0.03	0.03	-0.01	0.07	0.09	0
Acidaminococcus.id.2167	genus	Homa-IR	0.02	0.03	0.03	0.03	0.02	-0.02	0.06	0.25	0
Adlercreutzia.id.812	genus	Homa-IR	0.05	0.03	0.02	0.03	0.04	0.00	0.08	0.08	0
Akkermansia.id.4037	genus	Homa-IR	0.02	0.03	-0.01	0.03	0.01	-0.03	0.05	0.78	0
Alistipes.id.968	genus	Homa-IR	-0.08	0.03	-0.03	0.03	-0.05	-0.09	-0.01	0.01	0
Alloprevotella.id.961	genus	Homa-IR	-0.05	0.03	0.04	0.03	0.00	-0.04	0.04	0.90	0.488
Anaerostipes.id.1991	genus	Homa-IR	0.03	0.03	0.00	0.03	0.01	-0.03	0.05	0.50	0
Anaerotruncus.id.2054	genus	Homa-IR	-0.08	0.03	-0.03	0.03	-0.06	-0.10	-0.02	0.01	0.601
Bacteroides.id.918	genus	Homa-IR	-0.04	0.03	-0.01	0.03	-0.03	-0.07	0.02	0.22	0
Barnesiella.id.944	genus	Homa-IR	-0.05	0.03	-0.02	0.03	-0.04	-0.08	0.00	0.07	0
Bifidobacterium.id.436	genus	Homa-IR	0.02	0.03	0.01	0.03	0.01	-0.03	0.05	0.51	0
Blautia.id.1992	genus	Homa-IR	0.05	0.03	0.03	0.03	0.04	0.00	0.08	0.04	0
Butyricicoccus.id.2055	genus	Homa-IR	-0.02	0.03	0.00	0.03	-0.01	-0.05	0.03	0.62	0
Butyricimonas.id.945	genus	Homa-IR	-0.05	0.03	-0.02	0.03	-0.04	-0.08	0.00	0.06	0
Butyrivibrio.id.1993	genus	Homa-IR	-0.05	0.03	-0.01	0.03	-0.03	-0.07	0.01	0.13	0.375
Catenibacterium.id.2153	genus	Homa-IR	0.01	0.03	-0.01	0.03	0.00	-0.04	0.04	0.88	0
ChristensenellaceaeR7group.id.11283	genus	Homa-IR	-0.10	0.03	-0.06	0.03	-0.08	-0.12	-0.04	0.00	0

© 2021 Chen Z et al. *JAMA Network Open.*

Clostridiumsensustricto1.id.1873	genus	Homa-IR	-0.03	0.03	-0.01	0.03	-0.02	-0.06	0.02	0.33	0
Collinsella.id.815	genus	Homa-IR	0.01	0.03	-0.01	0.03	0.00	-0.04	0.04	0.99	0.123

© 2021 Chen Z et al. *JAMA Network Open.*

Coprococcus1.id.11301		genus	Homa-IR	-0.06	0.03	-0.03	0.03	-0.04	-0.08	0.00	0.04	0
Coprococcus2.id.11302		genus	Homa-IR	-0.05	0.03	-0.01	0.03	-0.03	-0.07	0.01	0.14	0
Coprococcus3.id.11303		genus	Homa-IR	0.01	0.03	0.01	0.03	0.01	-0.03	0.05	0.68	0
Desulfovibrio.id.3173		genus	Homa-IR	-0.02	0.03	-0.01	0.03	-0.02	-0.06	0.02	0.43	0.33
Dialister.id.2183		genus	Homa-IR	0.05	0.03	-0.02	0.03	0.02	-0.02	0.06	0.45	0.635
Dorea.id.1997		genus	Homa-IR	0.02	0.03	-0.02	0.03	0.00	-0.04	0.04	0.98	0.45
Eggerthella.id.819		genus	Homa-IR	0.00	0.03	0.07	0.03	0.04	-0.01	0.08	0.09	0.739
Eisenbergiella.id.11304		genus	Homa-IR	-0.02	0.03	0.03	0.03	0.00	-0.04	0.04	0.94	0.249
Enterococcus.id.1831		genus	Homa-IR	0.05	0.03	-0.02	0.03	0.02	-0.02	0.06	0.35	0
Enterorhabdus.id.820		genus	Homa-IR	-0.04	0.03	-0.02	0.03	-0.03	-0.07	0.01	0.11	0
Erysipelatoclostridium.id.11381		genus	Homa-IR	-0.01	0.03	0.06	0.03	0.02	-0.02	0.06	0.27	0.396
ErysipelotrichaceaeUCG003.id.11384		genus	Homa-IR	0.03	0.03	0.01	0.03	0.02	-0.02	0.06	0.36	0
Faecalibacterium.id.2057		genus	Homa-IR	-0.03	0.03	0.02	0.03	0.00	-0.04	0.04	0.86	0.613
FamilyXIIIAD3011group.id.11293		genus	Homa-IR	-0.05	0.03	-0.06	0.03	-0.06	-0.10	-0.02	0.00	0
FamilyXIIIUCG001.id.11294		genus	Homa-IR	0.00	0.03	-0.03	0.03	-0.01	-0.05	0.03	0.52	0
Fusicatenibacter.id.11305		genus	Homa-IR	0.03	0.03	0.02	0.03	0.03	-0.02	0.07	0.22	0
Haemophilus.id.3698		genus	Homa-IR	-0.02	0.03	-0.05	0.03	-0.03	-0.07	0.01	0.09	0.432
Holdemanella.id.11393		genus	Homa-IR	0.01	0.03	0.06	0.03	0.04	0.00	0.08	0.07	0
Intestinibacter.id.11345		genus	Homa-IR	-0.01	0.03	0.01	0.03	0.00	-0.04	0.04	0.98	0
Intestinimonas.id.2062		genus	Homa-IR	-0.07	0.03	-0.01	0.03	-0.04	-0.08	0.00	0.03	0
Lachnoclostridium.id.11308		genus	Homa-IR	0.00	0.03	0.04	0.03	0.02	-0.02	0.06	0.41	0.668
Lachnospira.id.2004		genus	Homa-IR	-0.03	0.03	0.03	0.03	0.00	-0.04	0.04	0.98	0
LachnospiraceaeFCS020group.id.11314		genus	Homa-IR	-0.03	0.03	-0.08	0.03	-0.05	-0.09	-0.01	0.01	0.346
LachnospiraceaeND3007group.id.11317		genus	Homa-IR	-0.04	0.03	0.00	0.03	-0.02	-0.06	0.02	0.26	0
LachnospiraceaeNK4A136group.id.11319		genus	Homa-IR	-0.07	0.03	-0.03	0.03	-0.05	-0.09	-0.01	0.02	0.686
LachnospiraceaeUCG001.id.11321		genus	Homa-IR	-0.02	0.03	-0.01	0.03	-0.02	-0.06	0.02	0.46	0
LachnospiraceaeUCG004.id.11324		genus	Homa-IR	-0.03	0.03	-0.04	0.03	-0.03	-0.07	0.01	0.10	0
LachnospiraceaeUCG010.id.11330		genus	Homa-IR	-0.02	0.03	-0.05	0.03	-0.03	-0.07	0.01	0.10	0
Lactobacillus.id.1837		genus	Homa-IR	-0.02	0.03	-0.03	0.03	-0.03	-0.07	0.01	0.19	0
Marvinbryantia.id.2005		genus	Homa-IR	-0.08	0.03	-0.07	0.03	-0.08	-0.12	-0.04	0.00	0
Megamonas.id.2184		genus	Homa-IR	0.02	0.03	-0.01	0.03	0.01	-0.03	0.05	0.62	0
Megasphaera.id.2185		genus	Homa-IR	0.01	0.03	0.01	0.03	0.01	-0.03	0.05	0.62	0.135

© 2021 Chen Z et al. *JAMA Network Open.*

Mitsuokella.id.2186	genus	Homa-IR	0.07	0.03	0.01	0.03	0.04	0.00	0.08	0.04	0
Odoribacter.id.952	genus	Homa-IR	-0.10	0.03	-0.03	0.03	-0.06	-0.10	-0.02	0.00	0

© 2021 Chen Z et al. *JAMA Network Open.*

Olsenella.id.822	genus	Homa-IR	0.00	0.03	-0.05	0.03	-0.02	-0.06	0.02	0.28	0.557
Oscillospira.id.2064	genus	Homa-IR	-0.05	0.03	-0.01	0.03	-0.03	-0.07	0.01	0.19	0
Parabacteroides.id.954	genus	Homa-IR	-0.03	0.03	-0.05	0.03	-0.04	-0.08	0.00	0.03	0
Paraprevotella.id.962	genus	Homa-IR	-0.03	0.03	-0.02	0.03	-0.02	-0.06	0.02	0.28	0
Parasutterella.id.2892	genus	Homa-IR	0.00	0.03	0.04	0.03	0.02	-0.02	0.06	0.30	0.214
Peptococcus.id.2037	genus	Homa-IR	-0.01	0.03	0.00	0.03	-0.01	-0.05	0.03	0.66	0
Phascolarctobacterium.id.2168	genus	Homa-IR	-0.06	0.03	-0.03	0.03	-0.04	-0.08	0.00	0.03	0
Prevotella2.id.11180	genus	Homa-IR	0.00	0.03	-0.02	0.03	-0.01	-0.05	0.03	0.75	0
Prevotella7.id.11182	genus	Homa-IR	-0.02	0.03	-0.01	0.03	-0.02	-0.06	0.02	0.43	0
Prevotella9.id.11183	genus	Homa-IR	-0.01	0.03	0.01	0.03	0.00	-0.04	0.04	1.00	0
PrevotellaceaeNK3B31group.id.11185	genus	Homa-IR	0.01	0.03	0.06	0.03	0.03	-0.01	0.07	0.10	0
PrevotellaceaeUCG001.id.11186	genus	Homa-IR	-0.01	0.03	0.02	0.03	0.00	-0.04	0.04	0.88	0.757
RikenellaceaeRC9gutgroup.id.11191	genus	Homa-IR	0.00	0.03	-0.04	0.03	-0.02	-0.06	0.02	0.37	0
Romboutsia.id.11347	genus	Homa-IR	-0.05	0.03	0.00	0.03	-0.03	-0.07	0.01	0.19	0.064
Roseburia.id.2012	genus	Homa-IR	0.01	0.03	0.02	0.03	0.02	-0.02	0.06	0.43	0
Ruminiclostridium5.id.11355	genus	Homa-IR	-0.07	0.03	-0.02	0.03	-0.04	-0.09	0.00	0.03	0.791
Ruminiclostridium6.id.11356	genus	Homa-IR	-0.09	0.03	0.01	0.03	-0.04	-0.08	0.00	0.04	0.869
Ruminiclostridium9.id.11357	genus	Homa-IR	-0.04	0.03	-0.08	0.03	-0.06	-0.10	-0.02	0.00	0.294
RuminococcaceaeNK4A214group.id.11358	genus	Homa-IR	-0.10	0.03	-0.08	0.03	-0.09	-0.13	-0.05	0.00	0
RuminococcaceaeUCG002.id.11360	genus	Homa-IR	-0.07	0.03	-0.08	0.03	-0.07	-0.11	-0.03	0.00	0
RuminococcaceaeUCG003.id.11361	genus	Homa-IR	-0.05	0.03	-0.09	0.03	-0.07	-0.11	-0.03	0.00	0.392
RuminococcaceaeUCG004.id.11362	genus	Homa-IR	0.02	0.03	0.02	0.03	0.02	-0.02	0.06	0.33	0
RuminococcaceaeUCG005.id.11363	genus	Homa-IR	-0.12	0.03	-0.07	0.03	-0.10	-0.14	-0.05	0.00	0.324
RuminococcaceaeUCG008.id.11365	genus	Homa-IR	-0.09	0.03	-0.04	0.03	-0.07	-0.10	-0.03	0.00	0.328
RuminococcaceaeUCG010.id.11367	genus	Homa-IR	-0.11	0.03	-0.06	0.03	-0.09	-0.13	-0.05	0.00	0
RuminococcaceaeUCG013.id.11370	genus	Homa-IR	-0.02	0.03	0.04	0.03	0.01	-0.03	0.05	0.57	0.666
RuminococcaceaeUCG014.id.11371	genus	Homa-IR	-0.07	0.03	-0.07	0.03	-0.07	-0.11	-0.03	0.00	0
Ruminococcus1.id.11373	genus	Homa-IR	-0.07	0.03	0.03	0.03	-0.02	-0.06	0.02	0.31	0.895
Ruminococcus2.id.11374	genus	Homa-IR	-0.04	0.03	0.03	0.03	0.00	-0.04	0.04	0.86	0
Sellimonas.id.14369	genus	Homa-IR	0.04	0.03	0.04	0.03	0.04	0.00	0.08	0.06	0
Senegalimassilia.id.11160	genus	Homa-IR	-0.01	0.03	-0.05	0.03	-0.03	-0.07	0.01	0.12	0.388
Slackia.id.825	genus	Homa-IR	0.03	0.03	-0.04	0.03	-0.01	-0.05	0.03	0.80	0.855

© 2021 Chen Z et al. *JAMA Network Open.*

Streptococcus.id.1853	genus	Homa-IR	0.04	0.03	0.06	0.03	0.05	0.01	0.09	0.02	0
Subdoligranulum.id.2070	genus	Homa-IR	-0.01	0.03	0.00	0.03	-0.01	-0.05	0.03	0.73	0

© 2021 Chen Z et al. *JAMA Network Open.*

Succiniclasticum.id.2169	genus	Homa-IR	0.00	0.03	0.00	0.03	0.00	-0.04	0.04	1.00	0
Sutterella.id.2896	genus	Homa-IR	-0.01	0.03	-0.02	0.03	-0.02	-0.06	0.02	0.40	0
Terrisporobacter.id.11348	genus	Homa-IR	-0.05	0.03	-0.02	0.03	-0.04	-0.08	0.00	0.08	0
Turicibacter.id.2162	genus	Homa-IR	-0.03	0.03	-0.01	0.03	-0.02	-0.06	0.02	0.27	0
Tyzzerella3.id.11335	genus	Homa-IR	0.03	0.03	0.00	0.03	0.02	-0.02	0.06	0.38	0
Veillonella.id.2198	genus	Homa-IR	-0.02	0.03	-0.03	0.03	-0.03	-0.07	0.01	0.21	0.475
unknowngenus.id.1000000073	genus	Homa-IR	-0.11	0.03	-0.03	0.03	-0.07	-0.11	-0.03	0.00	0.613
unknowngenus.id.1000001215	genus	Homa-IR	-0.08	0.03	0.01	0.03	-0.03	-0.08	0.01	0.09	0.673
unknowngenus.id.1000005472	genus	Homa-IR	-0.06	0.03	-0.06	0.03	-0.06	-0.10	-0.02	0.00	0
unknowngenus.id.1000005479	genus	Homa-IR	-0.04	0.03	-0.05	0.03	-0.04	-0.08	0.00	0.03	0
unknowngenus.id.1000013899	genus	Homa-IR	0.01	0.03	-0.06	0.03	-0.02	-0.06	0.02	0.31	0
unknowngenus.id.2071	genus	Homa-IR	-0.09	0.03	-0.03	0.03	-0.06	-0.10	-0.02	0.01	0.81
unknowngenus.id.2755	genus	Homa-IR	-0.08	0.03	0.01	0.03	-0.04	-0.08	0.00	0.06	0.459
unknowngenus.id.826	genus	Homa-IR	-0.03	0.03	0.01	0.03	-0.01	-0.05	0.03	0.54	0.291
unknowngenus.id.964	genus	Homa-IR	0.05	0.03	0.01	0.03	0.03	-0.01	0.07	0.11	0

**Taxa in RS only**	**Clade**	**Phenotype**	**Beta_RS**	**Se_RS**
BacteroidalesS247group.id.11173	family	Homa-IR	-0.05	0.03
Veillonellaceae.id.2172	family	Homa-IR	0.06	0.03
Bacteroidespectinophilusgroup.id.14371	genus	Homa-IR	-0.02	0.03
Enterobacter.id.3502	genus	Homa-IR	0.04	0.03
EscherichiaShigella.id.3504	genus	Homa-IR	0.01	0.03
Eubacteriumcoprostanoligenesgroup.id.11375	genus	Homa-IR	-0.07	0.03
Eubacteriumeligensgroup.id.14372	genus	Homa-IR	-0.09	0.03
Eubacteriumhalliigroup.id.11338	genus	Homa-IR	0.07	0.03
Eubacteriumoxidoreducensgroup.id.11339	genus	Homa-IR	-0.10	0.03
Eubacteriumxylanophilumgroup.id.14375	genus	Homa-IR	-0.07	0.03
Hungatella.id.11306	genus	Homa-IR	-0.01	0.03
Klebsiella.id.3507	genus	Homa-IR	0.05	0.03
LachnospiraceaeNC2004group.id.11316	genus	Homa-IR	-0.08	0.03

© 2021 Chen Z et al. *JAMA Network Open.*

LachnospiraceaeUCG008.id.11328	genus	Homa-IR	-0.03	0.03
unknowngenus.id.1868	genus	Homa-IR	-0.06	0.03

© 2021 Chen Z et al. *JAMA Network Open.*

Taxa in LLD only	Clade	Phenotype	Beta_LLD	Se_LLD
Archaea.id.2	domain	Homa-IR	-0.07	0.03
Euryarchaeota.id.55	phylum	Homa-IR	-0.07	0.03
Lentisphaerae.id.2238	phylum	Homa-IR	-0.02	0.03
Actinomycetales.id.420	order	Homa-IR	0.05	0.03
Mycoplasmatales.id.3946	order	Homa-IR	-0.03	0.03
NB1.n.id.3953	order	Homa-IR	0.00	0.03
Pseudomonadales.id.3709	order	Homa-IR	-0.03	0.03
Spirochaetales.id.3870	order	Homa-IR	0.00	0.03
Synergistales.id.3900	order	Homa-IR	-0.02	0.03
Thermoplasmatales.id.220	order	Homa-IR	-0.02	0.03
unknownorder.id.1000000003	order	Homa-IR	0.01	0.03
Verrucomicrobiales.id.4030	order	Homa-IR	-0.01	0.03
Victivallales.id.2254	order	Homa-IR	-0.02	0.03
Xanthomonadales.id.3786	order	Homa-IR	-0.03	0.03
Spirochaetes.id.3856	class	Homa-IR	0.00	0.03
Synergistia.id.3899	class	Homa-IR	-0.02	0.03
Thermoplasmata.id.210	class	Homa-IR	-0.02	0.03
Actinomycetaceae.id.421	family	Homa-IR	0.05	0.03
BacteroidalesS24.7group.id.11173	family	Homa-IR	-0.05	0.03
Christensenellaceae.id.1866	family	Homa-IR	-0.07	0.03
FamilyXI.id.1936	family	Homa-IR	0.01	0.03
unknownfamily.id.1000000004	family	Homa-IR	0.01	0.03
unknownfamily.id.1000006161	family	Homa-IR	0.00	0.03
unknownfamily.id.1855	family	Homa-IR	-0.03	0.03
vadinBE97.id.14446	family	Homa-IR	-0.02	0.03
Veillonellaceae.id.2172	family	Homa-IR	-0.02	0.03
Xanthomonadaceae.id.3799	family	Homa-IR	-0.03	0.03
Clostridiuminnocuumgroup.id.14397	genus	Homa-IR	0.05	0.03
Eubacteriumbrachygroup.id.11296	genus	Homa-IR	0.05	0.03
Abiotrophia.id.1803	genus	Homa-IR	0.00	0.03
Actinomyces.id.423	genus	Homa-IR	0.05	0.03

© 2021 Chen Z et al. *JAMA Network Open.*

Actinotignum.id.11137	genus	Homa-IR	-0.02	0.03
Allisonella.id.2174	genus	Homa-IR	0.04	0.03

© 2021 Chen Z et al. *JAMA Network Open.*

Arcanobacterium.id.424	genus	Homa-IR	0.00	0.03
Asaccharobacter.id.813	genus	Homa-IR	0.02	0.03
Bilophila.id.3170	genus	Homa-IR	0.00	0.03
CandidatusMethanogranum.id.11110	genus	Homa-IR	-0.02	0.03
Catenisphaera.id.14395	genus	Homa-IR	-0.03	0.03
Cellulosilyticum.id.1995	genus	Homa-IR	-0.05	0.03
Cetobacterium.id.2209	genus	Homa-IR	0.02	0.03
Cloacibacillus.id.3908	genus	Homa-IR	-0.01	0.03
Coprobacillus.id.2154	genus	Homa-IR	-0.01	0.03
CoriobacteriaceaeUCG.002.id.11158	genus	Homa-IR	-0.03	0.03
CoriobacteriaceaeUCG.003.id.11159	genus	Homa-IR	0.05	0.03
Denitrobacterium.id.818	genus	Homa-IR	-0.03	0.03
dgA.11gutgroup.id.978	genus	Homa-IR	0.02	0.03
Dielma.id.11380	genus	Homa-IR	0.00	0.03
Epulopiscium.id.1998	genus	Homa-IR	0.03	0.03
ErysipelotrichaceaeUCG006.id.11386	genus	Homa-IR	0.02	0.03
ErysipelotrichaceaeUCG009.id.11389	genus	Homa-IR	0.04	0.03
Escherichia.Shigella.id.3504	genus	Homa-IR	0.00	0.03
Eubacterium.id.1932	genus	Homa-IR	0.04	0.03
Faecalicoccus.id.11391	genus	Homa-IR	0.00	0.03
Faecalitalea.id.11392	genus	Homa-IR	0.05	0.03
Flavonifractor.id.2059	genus	Homa-IR	0.00	0.03
Gardnerella.id.437	genus	Homa-IR	0.00	0.03
Gordonibacter.id.821	genus	Homa-IR	0.03	0.03
Hafnia.Obesumbacterium.id.14636	genus	Homa-IR	0.00	0.03
Howardella.id.2000	genus	Homa-IR	-0.01	0.03
LachnospiraceaeNK3A20group.id.11318	genus	Homa-IR	0.03	0.03
Lactococcus.id.1851	genus	Homa-IR	0.00	0.03
Lautropia.id.2905	genus	Homa-IR	0.01	0.03
Leuconostoc.id.1841	genus	Homa-IR	-0.02	0.03
Methanobrevibacter.id.123	genus	Homa-IR	-0.07	0.03
Methanosphaera.id.124	genus	Homa-IR	-0.04	0.03

© 2021 Chen Z et al. *JAMA Network Open.*

Mogibacterium.id.1960	genus	Homa-IR	-0.02	0.03
Morganella.id.3512	genus	Homa-IR	-0.03	0.03

© 2021 Chen Z et al. *JAMA Network Open.*

Porphyromonas.id.956	genus	Homa-IR	0.01	0.03
Prevotella1.id.11179	genus	Homa-IR	0.01	0.03
PrevotellaceaeUCG003.id.11187	genus	Homa-IR	0.07	0.03
PrevotellaceaeUCG004.id.11188	genus	Homa-IR	-0.05	0.03
Pseudomonas.id.3723	genus	Homa-IR	-0.03	0.03
Rikenella.id.973	genus	Homa-IR	-0.01	0.03
RuminococcaceaeNK4A214group.id.11358	genus	Homa-IR	-0.08	0.03
RuminococcaceaeUCG005.id.11363	genus	Homa-IR	-0.07	0.03
RuminococcaceaeUCG008.id.11365	genus	Homa-IR	-0.04	0.03
RuminococcaceaeUCG010.id.11367	genus	Homa-IR	-0.06	0.03
Sarcina.id.1896	genus	Homa-IR	0.06	0.03
Solobacterium.id.2161	genus	Homa-IR	0.02	0.03
Staphylococcus.id.1780	genus	Homa-IR	0.05	0.03
Stenotrophomonas.id.3818	genus	Homa-IR	-0.03	0.03
Succinivibrio.id.3331	genus	Homa-IR	0.01	0.03
Synergistes.id.3913	genus	Homa-IR	-0.01	0.03
Syntrophococcus.id.2016	genus	Homa-IR	-0.01	0.03
Treponema2.id.11577	genus	Homa-IR	0.00	0.03
Tyzzerella4.id.11336	genus	Homa-IR	0.05	0.03
unknowngenus.id.1000000005	genus	Homa-IR	0.01	0.03
unknowngenus.id.1000027565	genus	Homa-IR	-0.02	0.03
unknowngenus.id.1000043796	genus	Homa-IR	-0.03	0.03
unknowngenus.id.1854	genus	Homa-IR	-0.04	0.03
unknowngenus.id.2163	genus	Homa-IR	-0.04	0.03
unknowngenus.id.3332	genus	Homa-IR	0.02	0.03
unknowngenus.id.441	genus	Homa-IR	-0.06	0.03
Ureaplasma.id.3951	genus	Homa-IR	-0.03	0.03
Varibaculum.id.427	genus	Homa-IR	0.04	0.03
Veillonella.id.2198	genus	Homa-IR	-0.03	0.03
Weissella.id.1843	genus	Homa-IR	0.01	0.03

© 2021 Chen Z et al. *JAMA Network Open.*

Supplementary Table 2 - Associations of taxa and insulin resistance

Effect estimates in RS and LLD were calculated using linear regression; pooled estimates were calculated based on a mixed-effect meta-analysis.

Model 4: Adjusted for age, sex, Time in mail (RS), Batch (RS), smoking, education level (RS), physical activity, alcohol intake, total energy intake, BMI, PPI, and lipid-lowering medication.

Abbreviation, RS, Rotterdam Study, LLD, Lifelines-Deep Study. P<0.0005 indicates statistical significance

Overlapping taxa in RS and LLD	Clade	Phenotype	Beta_RS	Se_RS	Beta_LLD	Se_LLD	Beta_Meta	Lower95%CI_Meta	Upper95%CI_Meta	p_Meta	I^2
Bacteria.id.3	domain	Homa-IR	0.00	0.03	0.03	0.02	0.02	-0.03	0.07	0.46	0.00
Actinobacteria.id.400	phylum	Homa-IR	0.02	0.03	0.00	0.03	0.01	-0.03	0.05	0.49	0.00
Bacteroidetes.id.905	phylum	Homa-IR	-0.03	0.03	-0.02	0.03	-0.03	-0.07	0.02	0.22	0.00
Cyanobacteria.id.1500	phylum	Homa-IR	-0.08	0.03	0.02	0.03	-0.03	-0.07	0.01	0.13	0.82
Firmicutes.id.1672	phylum	Homa-IR	0.00	0.03	0.04	0.03	0.02	-0.02	0.06	0.31	0.00
Proteobacteria.id.2375	phylum	Homa-IR	0.00	0.03	-0.02	0.03	-0.01	-0.05	0.03	0.56	0.00
Tenericutes.id.3919	phylum	Homa-IR	-0.07	0.03	-0.06	0.03	-0.06	-0.10	-0.02	0.00	0.00
Verrucomicrobia.id.3982	phylum	Homa-IR	0.02	0.03	-0.02	0.03	0.00	-0.04	0.04	0.92	0.02
Actinobacteria.id.419	class	Homa-IR	0.02	0.03	0.01	0.03	0.01	-0.03	0.05	0.50	0.00
Alphaproteobacteria.id.2379	class	Homa-IR	-0.08	0.03	0.00	0.03	-0.04	-0.08	0.00	0.05	0.75
Bacilli.id.1673	class	Homa-IR	0.02	0.03	0.03	0.03	0.02	-0.02	0.06	0.32	0.00
Bacteroidia.id.912	class	Homa-IR	-0.03	0.03	-0.02	0.03	-0.03	-0.07	0.02	0.22	0.00
Betaproteobacteria.id.2867	class	Homa-IR	-0.01	0.03	0.01	0.03	0.00	-0.04	0.04	0.87	0.00
Clostridia.id.1859	class	Homa-IR	-0.02	0.03	0.02	0.03	0.00	-0.04	0.04	0.91	0.00
Coriobacteriia.id.809	class	Homa-IR	0.00	0.03	-0.01	0.03	0.00	-0.04	0.04	0.91	0.00
Deltaproteobacteria.id.3087	class	Homa-IR	-0.02	0.03	-0.01	0.03	-0.01	-0.05	0.03	0.53	0.00
Erysipelotrichia.id.2147	class	Homa-IR	0.04	0.03	0.04	0.03	0.04	0.00	0.08	0.05	0.00
Gammaproteobacteria.id.3303	class	Homa-IR	-0.01	0.03	-0.02	0.03	-0.02	-0.06	0.02	0.44	0.00
Melainabacteria.id.1589	class	Homa-IR	-0.07	0.03	0.02	0.03	-0.03	-0.07	0.01	0.16	0.76
Mollicutes.id.3920	class	Homa-IR	-0.07	0.03	-0.06	0.03	-0.06	-0.10	-0.02	0.00	0.00
Negativicutes.id.2164	class	Homa-IR	0.01	0.03	-0.02	0.03	0.00	-0.04	0.04	0.90	0.00
Verrucomicrobiae.id.4029	class	Homa-IR	0.02	0.03	-0.02	0.03	0.00	-0.04	0.04	0.91	0.03
Aeromonadales.id.3316	order	Homa-IR	-0.02	0.03	0.00	0.03	-0.01	-0.05	0.03	0.76	0.00
Bacteroidales.id.913	order	Homa-IR	-0.03	0.03	-0.02	0.03	-0.03	-0.07	0.02	0.22	0.00
Bifidobacteriales.id.432	order	Homa-IR	0.01	0.03	0.01	0.03	0.01	-0.03	0.05	0.60	0.00
Burkholderiales.id.2874	order	Homa-IR	0.00	0.03	0.01	0.03	0.01	-0.03	0.05	0.78	0.00
Clostridiales.id.1863	order	Homa-IR	-0.02	0.03	0.02	0.03	0.00	-0.04	0.04	0.92	0.00

© 2021 Chen Z et al. *JAMA Network Open.*

Coriobacteriales.id.810	order	Homa-IR	0.00	0.03	-0.01	0.03	0.00	-0.04	0.04	0.91	0.00
Desulfovibrionales.id.3156	order	Homa-IR	-0.02	0.03	-0.01	0.03	-0.01	-0.05	0.03	0.54	0.00

© 2021 Chen Z et al. *JAMA Network Open.*

Enterobacteriales.id.3468	order	Homa-IR	0.01	0.03	0.01	0.03	0.01	-0.03	0.05	0.77	0.00
Erysipelotrichales.id.2148	order	Homa-IR	0.04	0.03	0.04	0.03	0.04	0.00	0.08	0.05	0.00
Gastranaerophilales.id.1591	order	Homa-IR	-0.07	0.03	0.02	0.03	-0.03	-0.07	0.01	0.17	0.76
Lactobacillales.id.1800	order	Homa-IR	0.02	0.03	0.03	0.03	0.02	-0.02	0.06	0.31	0.00
MollicutesRF9.id.11579	order	Homa-IR	-0.06	0.03	-0.06	0.03	-0.06	-0.10	-0.02	0.00	0.00
Pasteurellales.id.3688	order	Homa-IR	-0.03	0.03	-0.05	0.03	-0.04	-0.08	0.00	0.06	0.00
Rhodospirillales.id.2667	order	Homa-IR	-0.08	0.03	0.00	0.03	-0.04	-0.08	0.00	0.05	0.76
Selenomonadales.id.2165	order	Homa-IR	0.01	0.03	-0.02	0.03	0.00	-0.04	0.04	0.90	0.00
Verrucomicrobiales.id.4030	order	Homa-IR	0.02	0.03	-0.02	0.03	0.00	-0.04	0.04	0.91	0.03
Acidaminococcaceae.id.2166	family	Homa-IR	-0.04	0.03	-0.02	0.03	-0.03	-0.07	0.01	0.12	0.00
Alcaligenaceae.id.2875	family	Homa-IR	0.01	0.03	0.02	0.03	0.01	-0.03	0.05	0.54	0.00
Bacteroidaceae.id.917	family	Homa-IR	-0.02	0.03	-0.02	0.03	-0.02	-0.06	0.02	0.33	0.00
Bifidobacteriaceae.id.433	family	Homa-IR	0.01	0.03	0.01	0.03	0.01	-0.03	0.05	0.60	0.00
Christensenellaceae.id.1866	family	Homa-IR	-0.09	0.03	-0.06	0.03	-0.08	-0.12	-0.03	0.00	0.00
Clostridiaceae1.id.1869	family	Homa-IR	-0.03	0.03	0.00	0.03	-0.02	-0.06	0.02	0.37	0.00
ClostridialesvadinBB60group.id.11286	family	Homa-IR	-0.10	0.03	-0.03	0.03	-0.06	-0.10	-0.02	0.00	0.63
Coriobacteriaceae.id.811	family	Homa-IR	0.00	0.03	-0.01	0.03	0.00	-0.04	0.04	0.91	0.00
Desulfovibrionaceae.id.3169	family	Homa-IR	-0.02	0.03	-0.01	0.03	-0.01	-0.05	0.03	0.53	0.00
Enterobacteriaceae.id.3469	family	Homa-IR	0.01	0.03	0.01	0.03	0.01	-0.03	0.05	0.77	0.00
Enterococcaceae.id.1828	family	Homa-IR	0.03	0.03	-0.04	0.03	0.00	-0.03	0.04	0.84	0.68
Erysipelotrichaceae.id.2149	family	Homa-IR	0.04	0.03	0.04	0.03	0.04	0.00	0.08	0.05	0.00
FamilyXIII.id.1957	family	Homa-IR	-0.03	0.03	-0.02	0.03	-0.03	-0.07	0.01	0.20	0.00
Lachnospiraceae.id.1987	family	Homa-IR	0.04	0.03	0.03	0.03	0.04	0.00	0.08	0.08	0.00
Lactobacillaceae.id.1836	family	Homa-IR	-0.02	0.03	-0.04	0.03	-0.03	-0.07	0.01	0.13	0.00
Pasteurellaceae.id.3689	family	Homa-IR	-0.03	0.03	-0.05	0.03	-0.04	-0.08	0.00	0.06	0.00
Peptococcaceae.id.2024	family	Homa-IR	-0.03	0.03	0.00	0.03	-0.02	-0.06	0.02	0.36	0.00
Peptostreptococcaceae.id.2042	family	Homa-IR	-0.04	0.03	0.01	0.03	-0.02	-0.06	0.02	0.33	0.27
Porphyromonadaceae.id.943	family	Homa-IR	-0.04	0.03	-0.05	0.03	-0.05	-0.09	-0.01	0.02	0.00
Prevotellaceae.id.960	family	Homa-IR	-0.01	0.03	0.02	0.03	0.00	-0.04	0.04	0.93	0.00
Rhodospirillaceae.id.2717	family	Homa-IR	-0.08	0.03	0.00	0.03	-0.04	-0.08	0.00	0.06	0.75
Rikenellaceae.id.967	family	Homa-IR	-0.06	0.03	-0.04	0.03	-0.05	-0.09	-0.01	0.02	0.00
Ruminococcaceae.id.2050	family	Homa-IR	-0.07	0.03	0.00	0.03	-0.04	-0.08	0.00	0.05	0.70

© 2021 Chen Z et al. *JAMA Network Open.*

Streptococcaceae.id.1850	family	Homa-IR	0.03	0.03	0.05	0.03	0.04	0.00	0.08	0.07	0.00
Succinivibrionaceae.id.3326	family	Homa-IR	-0.02	0.03	0.00	0.03	-0.01	-0.05	0.03	0.74	0.00

© 2021 Chen Z et al. *JAMA Network Open.*

Veillonellaceae.id.2172	family	Homa-IR	0.05	0.03	-0.02	0.03	0.02	-0.02	0.06	0.32	0.63
Verrucomicrobiaceae.id.4036	family	Homa-IR	0.02	0.03	-0.02	0.03	0.00	-0.04	0.04	0.91	0.03
unknownfamily.id.1000001214	family	Homa-IR	-0.07	0.03	0.02	0.03	-0.03	-0.07	0.01	0.17	0.76
unknownfamily.id.1000005471	family	Homa-IR	-0.06	0.03	-0.06	0.03	-0.06	-0.10	-0.02	0.00	0.00
unknownfamily.id.987	family	Homa-IR	0.01	0.03	-0.05	0.03	-0.02	-0.06	0.02	0.35	0.61
Bacteroidespectinophilusgroup.id.14371	genus	Homa-IR	-0.02	0.03	-0.01	0.03	-0.02	-0.06	0.02	0.41	0.00
Eubacteriumcoprostanoligenesgroup.id.11375	genus	Homa-IR	0.00	0.03	-0.02	0.03	-0.01	-0.05	0.03	0.65	0.00
Eubacteriumeligensgroup.id.14372	genus	Homa-IR	-0.09	0.03	-0.05	0.03	-0.07	-0.11	-0.03	0.00	0.00
Eubacteriumhalliigroup.id.11338	genus	Homa-IR	0.06	0.03	0.02	0.03	0.04	0.00	0.08	0.04	0.18
Eubacteriumrectalegroup.id.14374	genus	Homa-IR	0.03	0.03	0.03	0.03	0.03	-0.01	0.07	0.19	0.00
Eubacteriumruminantiumgroup.id.11340	genus	Homa-IR	-0.03	0.03	-0.03	0.03	-0.03	-0.07	0.01	0.11	0.00
Eubacteriumventriosumgroup.id.11341	genus	Homa-IR	0.04	0.03	0.03	0.03	0.04	0.00	0.08	0.07	0.00
Eubacteriumxylanophilumgroup.id.14375	genus	Homa-IR	-0.05	0.03	-0.03	0.03	-0.04	-0.08	0.00	0.05	0.00
Ruminococcusgauvreauiigroup.id.11342	genus	Homa-IR	0.00	0.03	0.01	0.03	0.00	-0.04	0.04	0.86	0.00
Ruminococcusgnavusgroup.id.14376	genus	Homa-IR	0.01	0.03	0.07	0.03	0.04	0.00	0.08	0.05	0.56
Ruminococcustorquesgroup.id.14377	genus	Homa-IR	0.06	0.03	0.00	0.03	0.04	0.00	0.07	0.08	0.59
Acidaminococcus.id.2167	genus	Homa-IR	0.02	0.03	0.03	0.03	0.02	-0.02	0.06	0.26	0.00
Adlercreutzia.id.812	genus	Homa-IR	0.05	0.03	0.02	0.03	0.04	0.00	0.08	0.06	0.00
Akkermansia.id.4037	genus	Homa-IR	0.02	0.03	-0.02	0.03	0.00	-0.04	0.04	0.92	0.03
Alistipes.id.968	genus	Homa-IR	-0.06	0.03	-0.03	0.03	-0.05	-0.09	-0.01	0.02	0.00
Alloprevotella.id.961	genus	Homa-IR	-0.04	0.03	0.04	0.03	0.00	-0.04	0.04	0.87	0.73
Anaerostipes.id.1991	genus	Homa-IR	0.02	0.03	0.00	0.03	0.01	-0.03	0.05	0.59	0.00
Anaerotruncus.id.2054	genus	Homa-IR	-0.07	0.03	-0.03	0.03	-0.05	-0.09	-0.01	0.01	0.00
Bacteroides.id.918	genus	Homa-IR	-0.02	0.03	-0.02	0.03	-0.02	-0.06	0.02	0.33	0.00
Barnesiella.id.944	genus	Homa-IR	-0.04	0.03	-0.02	0.03	-0.03	-0.07	0.01	0.11	0.00
Bifidobacterium.id.436	genus	Homa-IR	0.01	0.03	0.02	0.03	0.01	-0.03	0.05	0.52	0.00
Blautia.id.1992	genus	Homa-IR	0.04	0.03	0.03	0.03	0.04	-0.01	0.08	0.09	0.00
Butyricicoccus.id.2055	genus	Homa-IR	-0.02	0.03	0.00	0.03	-0.01	-0.05	0.03	0.52	0.00
Butyricimonas.id.945	genus	Homa-IR	-0.05	0.03	-0.02	0.03	-0.03	-0.07	0.01	0.10	0.00
Butyrivibrio.id.1993	genus	Homa-IR	-0.04	0.03	-0.01	0.03	-0.03	-0.07	0.01	0.20	0.00
Catenibacterium.id.2153	genus	Homa-IR	0.01	0.03	-0.01	0.03	0.00	-0.04	0.04	0.94	0.00
ChristensenellaceaeR7group.id.11283	genus	Homa-IR	-0.09	0.03	-0.06	0.03	-0.07	-0.12	-0.03	0.00	0.00

© 2021 Chen Z et al. *JAMA Network Open.*

Clostridiumsensustricto1.id.1873	genus	Homa-IR	-0.03	0.03	0.00	0.03	-0.02	-0.06	0.02	0.35	0.00
Collinsella.id.815	genus	Homa-IR	0.01	0.03	-0.01	0.03	0.00	-0.04	0.04	0.95	0.00

© 2021 Chen Z et al. *JAMA Network Open.*

Coprococcus1.id.11301		genus	Homa-IR	-0.05	0.03	-0.03	0.03	-0.04	-0.08	0.00	0.05	0.00
Coprococcus2.id.11302		genus	Homa-IR	-0.04	0.03	0.00	0.03	-0.02	-0.06	0.02	0.25	0.09
Coprococcus3.id.11303		genus	Homa-IR	0.01	0.03	0.01	0.03	0.01	-0.03	0.05	0.65	0.00
Desulfovibrio.id.3173		genus	Homa-IR	-0.01	0.03	-0.01	0.03	-0.01	-0.05	0.03	0.58	0.00
Dialister.id.2183		genus	Homa-IR	0.04	0.03	-0.02	0.03	0.01	-0.03	0.05	0.59	0.44
Dorea.id.1997		genus	Homa-IR	0.03	0.03	-0.02	0.03	0.01	-0.03	0.05	0.73	0.35
Eggerthella.id.819		genus	Homa-IR	0.00	0.03	0.07	0.03	0.03	-0.01	0.08	0.09	0.68
Eisenbergiella.id.11304		genus	Homa-IR	-0.03	0.03	0.03	0.03	0.00	-0.04	0.04	0.95	0.44
Enterococcus.id.1831		genus	Homa-IR	0.03	0.03	-0.02	0.03	0.01	-0.03	0.05	0.62	0.45
Enterorhabdus.id.820		genus	Homa-IR	-0.04	0.03	-0.02	0.03	-0.03	-0.07	0.01	0.15	0.00
Erysipelatoclostridium.id.11381		genus	Homa-IR	-0.02	0.03	0.06	0.03	0.02	-0.02	0.06	0.38	0.70
ErysipelotrichaceaeUCG003.id.11384		genus	Homa-IR	0.04	0.03	0.01	0.03	0.03	-0.01	0.07	0.21	0.00
Faecalibacterium.id.2057		genus	Homa-IR	-0.01	0.03	0.03	0.03	0.01	-0.03	0.05	0.74	0.00
FamilyXIIIAD3011group.id.11293		genus	Homa-IR	-0.05	0.03	-0.06	0.03	-0.06	-0.10	-0.02	0.01	0.00
FamilyXIIIUCG001.id.11294		genus	Homa-IR	0.01	0.03	-0.02	0.03	-0.01	-0.05	0.03	0.78	0.00
Fusicatenibacter.id.11305		genus	Homa-IR	0.04	0.03	0.02	0.03	0.03	-0.01	0.07	0.19	0.00
Haemophilus.id.3698		genus	Homa-IR	-0.03	0.03	-0.05	0.03	-0.04	-0.08	0.00	0.05	0.00
Holdemanella.id.11393		genus	Homa-IR	0.01	0.03	0.06	0.03	0.03	-0.01	0.07	0.09	0.38
Intestinibacter.id.11345		genus	Homa-IR	-0.02	0.03	0.02	0.03	0.00	-0.04	0.04	0.88	0.05
Intestinimonas.id.2062		genus	Homa-IR	-0.06	0.03	-0.02	0.03	-0.04	-0.08	0.00	0.04	0.31
Lachnoclostridium.id.11308		genus	Homa-IR	0.00	0.03	0.03	0.03	0.01	-0.03	0.05	0.55	0.00
Lachnospira.id.2004		genus	Homa-IR	-0.02	0.03	0.03	0.03	0.01	-0.03	0.05	0.78	0.13
LachnospiraceaeFCS020group.id.11314		genus	Homa-IR	-0.02	0.03	-0.07	0.03	-0.05	-0.09	-0.01	0.03	0.45
LachnospiraceaeND3007group.id.11317		genus	Homa-IR	-0.03	0.03	0.00	0.03	-0.02	-0.06	0.02	0.42	0.00
LachnospiraceaeNK4A136group.id.11319		genus	Homa-IR	-0.05	0.03	-0.03	0.03	-0.04	-0.08	0.00	0.04	0.00
LachnospiraceaeUCG001.id.11321		genus	Homa-IR	-0.01	0.03	-0.01	0.03	-0.01	-0.05	0.03	0.64	0.00
LachnospiraceaeUCG004.id.11324		genus	Homa-IR	-0.02	0.03	-0.03	0.03	-0.03	-0.07	0.01	0.20	0.00
LachnospiraceaeUCG010.id.11330		genus	Homa-IR	-0.01	0.03	-0.05	0.03	-0.03	-0.07	0.01	0.14	0.00
Lactobacillus.id.1837		genus	Homa-IR	-0.02	0.03	-0.03	0.03	-0.02	-0.06	0.02	0.23	0.00
Marvinbryantia.id.2005		genus	Homa-IR	-0.07	0.03	-0.08	0.03	-0.07	-0.11	-0.03	0.00	0.00
Megamonas.id.2184		genus	Homa-IR	0.03	0.03	0.00	0.03	0.01	-0.03	0.05	0.54	0.00
Megasphaera.id.2185		genus	Homa-IR	0.01	0.03	0.01	0.03	0.01	-0.03	0.05	0.56	0.00

© 2021 Chen Z et al. *JAMA Network Open.*

Mitsuokella.id.2186	genus	Homa-IR	0.07	0.03	0.01	0.03	0.04	0.00	0.08	0.03	0.46
Odoribacter.id.952	genus	Homa-IR	-0.08	0.03	-0.03	0.03	-0.06	-0.10	-0.02	0.01	0.44

© 2021 Chen Z et al. *JAMA Network Open.*

Olsenella.id.822		genus	Homa-IR	0.00	0.03	-0.05	0.03	-0.02	-0.06	0.02	0.31	0.27
Oscillospira.id.2064		genus	Homa-IR	-0.04	0.03	-0.01	0.03	-0.02	-0.06	0.02	0.28	0.00
Parabacteroides.id.954		genus	Homa-IR	-0.02	0.03	-0.06	0.03	-0.04	-0.08	0.00	0.05	0.00
Paraprevotella.id.962		genus	Homa-IR	-0.02	0.03	-0.01	0.03	-0.02	-0.06	0.02	0.33	0.00
Parasutterella.id.2892		genus	Homa-IR	0.00	0.03	0.04	0.03	0.02	-0.02	0.06	0.27	0.00
Peptococcus.id.2037		genus	Homa-IR	-0.01	0.03	0.00	0.03	-0.01	-0.05	0.03	0.70	0.00
Phascolarctobacterium.id.2168		genus	Homa-IR	-0.05	0.03	-0.02	0.03	-0.04	-0.08	0.00	0.05	0.00
Prevotella2.id.11180		genus	Homa-IR	0.01	0.03	-0.01	0.03	0.00	-0.04	0.04	0.85	0.00
Prevotella7.id.11182		genus	Homa-IR	-0.01	0.03	-0.01	0.03	-0.01	-0.05	0.03	0.54	0.00
Prevotella9.id.11183		genus	Homa-IR	-0.01	0.03	0.01	0.03	0.00	-0.04	0.04	1.00	0.00
PrevotellaceaeNK3B31group.id.11185		genus	Homa-IR	0.01	0.03	0.06	0.03	0.03	-0.01	0.07	0.12	0.38
PrevotellaceaeUCG001.id.11186		genus	Homa-IR	0.00	0.03	0.02	0.03	0.01	-0.03	0.05	0.75	0.00
RikenellaceaeRC9gutgroup.id.11191		genus	Homa-IR	0.00	0.03	-0.03	0.03	-0.02	-0.06	0.02	0.42	0.00
Romboutsia.id.11347		genus	Homa-IR	-0.04	0.03	0.00	0.03	-0.02	-0.06	0.02	0.27	0.13
Roseburia.id.2012		genus	Homa-IR	0.02	0.03	0.03	0.03	0.02	-0.02	0.06	0.27	0.00
Ruminiclostridium5.id.11355		genus	Homa-IR	-0.07	0.03	-0.02	0.03	-0.05	-0.09	-0.01	0.03	0.02
Ruminiclostridium6.id.11356		genus	Homa-IR	-0.08	0.03	0.01	0.03	-0.04	-0.08	0.00	0.08	0.79
Ruminiclostridium9.id.11357		genus	Homa-IR	-0.04	0.03	-0.09	0.03	-0.06	-0.10	-0.02	0.00	0.31
RuminococcaceaeNK4A214group.id.11358		genus	Homa-IR	-0.09	0.03	-0.08	0.03	-0.09	-0.13	-0.05	0.00	0.00
RuminococcaceaeUCG002.id.11360		genus	Homa-IR	-0.06	0.03	-0.07	0.03	-0.06	-0.10	-0.02	0.00	0.00
RuminococcaceaeUCG003.id.11361		genus	Homa-IR	-0.03	0.03	-0.08	0.03	-0.06	-0.10	-0.02	0.00	0.27
RuminococcaceaeUCG004.id.11362		genus	Homa-IR	0.02	0.03	0.02	0.03	0.02	-0.02	0.06	0.36	0.00
RuminococcaceaeUCG005.id.11363		genus	Homa-IR	-0.11	0.03	-0.07	0.03	-0.09	-0.13	-0.05	0.00	0.08
RuminococcaceaeUCG008.id.11365		genus	Homa-IR	-0.10	0.03	-0.04	0.03	-0.07	-0.11	-0.03	0.00	0.56
RuminococcaceaeUCG010.id.11367		genus	Homa-IR	-0.10	0.03	-0.06	0.03	-0.08	-0.12	-0.04	0.00	0.09
RuminococcaceaeUCG013.id.11370		genus	Homa-IR	-0.01	0.03	0.04	0.03	0.01	-0.03	0.05	0.58	0.25
RuminococcaceaeUCG014.id.11371		genus	Homa-IR	-0.06	0.03	-0.06	0.03	-0.06	-0.10	-0.02	0.00	0.00
Ruminococcus1.id.11373		genus	Homa-IR	-0.05	0.03	0.03	0.03	-0.01	-0.05	0.03	0.59	0.72
Ruminococcus2.id.11374		genus	Homa-IR	-0.02	0.03	0.04	0.03	0.01	-0.03	0.05	0.72	0.44
Sellimonas.id.14369		genus	Homa-IR	0.03	0.03	0.03	0.03	0.03	-0.01	0.07	0.13	0.00
Senegalimassilia.id.11160		genus	Homa-IR	-0.01	0.03	-0.05	0.03	-0.03	-0.07	0.01	0.14	0.10
Slackia.id.825		genus	Homa-IR	0.03	0.03	-0.05	0.03	-0.01	-0.05	0.03	0.71	0.69

© 2021 Chen Z et al. *JAMA Network Open.*

Streptococcus.id.1853	genus	Homa-IR	0.03	0.03	0.05	0.03	0.04	0.00	0.08	0.06	0.00
Subdoligranulum.id.2070	genus	Homa-IR	0.00	0.03	0.00	0.03	0.00	-0.04	0.04	0.86	0.00

© 2021 Chen Z et al. *JAMA Network Open.*

Succiniclasticum.id.2169		genus	Homa-IR	0.01	0.03	0.00	0.03	0.00	-0.04	0.04	0.88	0.00
Sutterella.id.2896		genus	Homa-IR	0.00	0.03	-0.02	0.03	-0.01	-0.05	0.03	0.56	0.00
Terrisporobacter.id.11348		genus	Homa-IR	-0.05	0.03	-0.02	0.03	-0.04	-0.08	0.00	0.06	0.00
Turicibacter.id.2162		genus	Homa-IR	-0.04	0.03	-0.01	0.03	-0.02	-0.06	0.02	0.22	0.00
Tyzzerella3.id.11335		genus	Homa-IR	0.04	0.03	0.00	0.03	0.02	-0.02	0.06	0.26	0.00
Veillonella.id.2198		genus	Homa-IR	-0.04	0.03	-0.03	0.03	-0.04	-0.08	0.01	0.09	0.00
unknowngenus.id.1000000073		genus	Homa-IR	-0.10	0.03	-0.03	0.03	-0.06	-0.10	-0.02	0.00	0.63
unknowngenus.id.1000001215		genus	Homa-IR	-0.07	0.03	0.02	0.03	-0.03	-0.07	0.01	0.17	0.76
unknowngenus.id.1000005472		genus	Homa-IR	-0.06	0.03	-0.06	0.03	-0.06	-0.10	-0.02	0.00	0.00
unknowngenus.id.1000005479		genus	Homa-IR	-0.03	0.03	-0.05	0.03	-0.04	-0.08	0.00	0.06	0.00
unknowngenus.id.1000013899		genus	Homa-IR	0.01	0.03	-0.05	0.03	-0.02	-0.06	0.02	0.35	0.61
unknowngenus.id.2071		genus	Homa-IR	-0.08	0.03	-0.03	0.03	-0.06	-0.10	-0.02	0.01	0.31
unknowngenus.id.2755		genus	Homa-IR	-0.08	0.03	0.01	0.03	-0.04	-0.08	0.00	0.07	0.75
unknowngenus.id.826		genus	Homa-IR	-0.02	0.03	0.01	0.03	-0.01	-0.05	0.03	0.67	0.00
unknowngenus.id.964		genus	Homa-IR	0.06	0.03	0.01	0.03	0.04	0.00	0.08	0.06	0.26

**Taxa in RS only**		**Clade**	**Phenotype**	**Beta_RS**	**Se_RS**
BacteroidalesS247group.id.11173		family	Homa-IR	-0.03	0.03
Veillonellaceae.id.2172		family	Homa-IR	0.05	0.03
Bacteroidespectinophilusgroup.id.14371		genus	Homa-IR	-0.02	0.03
Enterobacter.id.3502		genus	Homa-IR	0.02	0.03
EscherichiaShigella.id.3504		genus	Homa-IR	0.00	0.03
Eubacteriumcoprostanoligenesgroup.id.11375		genus	Homa-IR	0.00	0.03
Eubacteriumeligensgroup.id.14372		genus	Homa-IR	-0.09	0.03
Eubacteriumhalliigroup.id.11338		genus	Homa-IR	0.06	0.03
Eubacteriumoxidoreducensgroup.id.11339		genus	Homa-IR	-0.09	0.03
Eubacteriumxylanophilumgroup.id.14375		genus	Homa-IR	-0.05	0.03
Hungatella.id.11306		genus	Homa-IR	-0.02	0.03
Klebsiella.id.3507		genus	Homa-IR	0.04	0.03
LachnospiraceaeNC2004group.id.11316		genus	Homa-IR	-0.07	0.03

© 2021 Chen Z et al. *JAMA Network Open.*

LachnospiraceaeUCG008.id.11328	genus	Homa-IR	-0.02	0.03
unknowngenus.id.1868	genus	Homa-IR	-0.05	0.03

© 2021 Chen Z et al. *JAMA Network Open.*

Taxa in LLD only	Clade	Phenotype	Beta_LLD	Se_LLD
Archaea.id.2	domain	Homa-IR	-0.07	0.03
Euryarchaeota.id.55	phylum	Homa-IR	-0.07	0.03
Lentisphaerae.id.2238	phylum	Homa-IR	-0.02	0.03
Actinomycetales.id.420	order	Homa-IR	0.05	0.03
Mycoplasmatales.id.3946	order	Homa-IR	-0.03	0.03
NB1.n.id.3953	order	Homa-IR	0.00	0.03
Pseudomonadales.id.3709	order	Homa-IR	-0.04	0.03
Spirochaetales.id.3870	order	Homa-IR	0.00	0.03
Synergistales.id.3900	order	Homa-IR	-0.01	0.03
Thermoplasmatales.id.220	order	Homa-IR	-0.02	0.03
unknownorder.id.1000000003	order	Homa-IR	0.02	0.03
Verrucomicrobiales.id.4030	order	Homa-IR	-0.02	0.03
Victivallales.id.2254	order	Homa-IR	-0.02	0.03
Xanthomonadales.id.3786	order	Homa-IR	-0.04	0.03
Spirochaetes.id.3856	class	Homa-IR	0.00	0.03
Synergistia.id.3899	class	Homa-IR	-0.01	0.03
Thermoplasmata.id.210	class	Homa-IR	-0.02	0.03
Actinomycetaceae.id.421	family	Homa-IR	0.05	0.03
BacteroidalesS24.7group.id.11173	family	Homa-IR	-0.05	0.03
Christensenellaceae.id.1866	family	Homa-IR	-0.06	0.03
FamilyXI.id.1936	family	Homa-IR	0.01	0.03
unknownfamily.id.1000000004	family	Homa-IR	0.02	0.03
unknownfamily.id.1000006161	family	Homa-IR	0.00	0.03
unknownfamily.id.1855	family	Homa-IR	-0.03	0.03
vadinBE97.id.14446	family	Homa-IR	-0.02	0.03
Veillonellaceae.id.2172	family	Homa-IR	-0.02	0.03
Xanthomonadaceae.id.3799	family	Homa-IR	-0.04	0.03
Clostridiuminnocuumgroup.id.14397	genus	Homa-IR	0.04	0.03
Eubacteriumbrachygroup.id.11296	genus	Homa-IR	0.05	0.03
Abiotrophia.id.1803	genus	Homa-IR	0.00	0.03
Actinomyces.id.423	genus	Homa-IR	0.05	0.03

© 2021 Chen Z et al. *JAMA Network Open.*

Actinotignum.id.11137	genus	Homa-IR	-0.02	0.03
Allisonella.id.2174	genus	Homa-IR	0.04	0.03

© 2021 Chen Z et al. *JAMA Network Open.*

Arcanobacterium.id.424		genus	Homa-IR	0.00	0.03
Asaccharobacter.id.813		genus	Homa-IR	0.02	0.03
Bilophila.id.3170		genus	Homa-IR	0.00	0.03
CandidatusMethanogranum.id.11110		genus	Homa-IR	-0.02	0.03
Catenisphaera.id.14395		genus	Homa-IR	-0.03	0.03
Cellulosilyticum.id.1995		genus	Homa-IR	-0.05	0.03
Cetobacterium.id.2209		genus	Homa-IR	0.02	0.03
Cloacibacillus.id.3908		genus	Homa-IR	-0.01	0.03
Coprobacillus.id.2154		genus	Homa-IR	0.00	0.03
CoriobacteriaceaeUCG.002.id.11158		genus	Homa-IR	-0.03	0.03
CoriobacteriaceaeUCG.003.id.11159		genus	Homa-IR	0.05	0.03
Denitrobacterium.id.818		genus	Homa-IR	-0.02	0.03
dgA.11gutgroup.id.978		genus	Homa-IR	0.02	0.03
Dielma.id.11380		genus	Homa-IR	0.00	0.03
Epulopiscium.id.1998		genus	Homa-IR	0.03	0.03
ErysipelotrichaceaeUCG006.id.11386		genus	Homa-IR	0.02	0.03
ErysipelotrichaceaeUCG009.id.11389		genus	Homa-IR	0.04	0.03
Escherichia.Shigella.id.3504		genus	Homa-IR	0.00	0.03
Eubacterium.id.1932		genus	Homa-IR	0.04	0.03
Faecalicoccus.id.11391		genus	Homa-IR	0.00	0.03
Faecalitalea.id.11392		genus	Homa-IR	0.05	0.03
Flavonifractor.id.2059		genus	Homa-IR	0.00	0.03
Gardnerella.id.437		genus	Homa-IR	0.00	0.03
Gordonibacter.id.821		genus	Homa-IR	0.03	0.03
Hafnia.Obesumbacterium.id.14636		genus	Homa-IR	-0.01	0.03
Howardella.id.2000		genus	Homa-IR	-0.02	0.03
LachnospiraceaeNK3A20group.id.11318		genus	Homa-IR	0.03	0.03
Lactococcus.id.1851		genus	Homa-IR	0.00	0.03
Lautropia.id.2905		genus	Homa-IR	0.01	0.03
Leuconostoc.id.1841		genus	Homa-IR	-0.02	0.03
Methanobrevibacter.id.123		genus	Homa-IR	-0.07	0.03
Methanosphaera.id.124		genus	Homa-IR	-0.04	0.03

© 2021 Chen Z et al. *JAMA Network Open.*

Mogibacterium.id.1960	genus	Homa-IR	-0.02	0.03
Morganella.id.3512	genus	Homa-IR	-0.03	0.03

© 2021 Chen Z et al. *JAMA Network Open.*

Porphyromonas.id.956	genus	Homa-IR	0.01	0.03
Prevotella1.id.11179	genus	Homa-IR	0.01	0.03
PrevotellaceaeUCG003.id.11187	genus	Homa-IR	0.07	0.03
PrevotellaceaeUCG004.id.11188	genus	Homa-IR	-0.05	0.03
Pseudomonas.id.3723	genus	Homa-IR	-0.04	0.03
Rikenella.id.973	genus	Homa-IR	-0.01	0.03
RuminococcaceaeNK4A214group.id.11358	genus	Homa-IR	-0.08	0.03
RuminococcaceaeUCG005.id.11363	genus	Homa-IR	-0.07	0.03
RuminococcaceaeUCG008.id.11365	genus	Homa-IR	-0.04	0.03
RuminococcaceaeUCG010.id.11367	genus	Homa-IR	-0.06	0.03
Sarcina.id.1896	genus	Homa-IR	0.05	0.03
Solobacterium.id.2161	genus	Homa-IR	0.02	0.03
Staphylococcus.id.1780	genus	Homa-IR	0.04	0.03
Stenotrophomonas.id.3818	genus	Homa-IR	-0.04	0.03
Succinivibrio.id.3331	genus	Homa-IR	0.00	0.03
Synergistes.id.3913	genus	Homa-IR	-0.01	0.03
Syntrophococcus.id.2016	genus	Homa-IR	-0.01	0.03
Treponema2.id.11577	genus	Homa-IR	0.00	0.03
Tyzzerella4.id.11336	genus	Homa-IR	0.05	0.03
unknowngenus.id.1000000005	genus	Homa-IR	0.02	0.03
unknowngenus.id.1000027565	genus	Homa-IR	-0.02	0.03
unknowngenus.id.1000043796	genus	Homa-IR	-0.03	0.03
unknowngenus.id.1854	genus	Homa-IR	-0.04	0.03
unknowngenus.id.2163	genus	Homa-IR	-0.05	0.03
unknowngenus.id.3332	genus	Homa-IR	0.02	0.03
unknowngenus.id.441	genus	Homa-IR	-0.06	0.03
Ureaplasma.id.3951	genus	Homa-IR	-0.03	0.03
Varibaculum.id.427	genus	Homa-IR	0.04	0.03
Veillonella.id.2198	genus	Homa-IR	-0.03	0.03
Weissella.id.1843	genus	Homa-IR	0.01	0.03

© 2021 Chen Z et al. *JAMA Network Open.*

eTable 3. Associations of Taxa and Type 2 Diabetes

Effect estimates in RS and LLD were calculated using logistic regression; pooled estimates were calculated based on a mixed-effect meta-analysis.

Model 1: Adjusted for age, sex, Time in mail (RS), and Batch (RS).

Abbreviation, RS, Rotterdam Study, LLD, Lifelines-Deep Study. P<0.0005 indicates statistical significance

**Overlapping taxa in RS and LLD**	**Clade**	**Phenotype**	**Beta_RS**	**Se_RS**	**Beta_LLD**	**Se_LLD**	**OR_Meta**	**Lower95%CI_Meta**	**Upper95%CI_Meta**	**P_Meta**	**I^2**
Bacteria.id.3	domain	T2D	0.12	0.09	0.29	0.23	1.15	0.97	1.37	0.10	0.00
Actinobacteria.id.400	phylum	T2D	-0.03	0.09	-0.08	0.31	0.97	0.81	1.16	0.73	0.00
Bacteroidetes.id.905	phylum	T2D	0.36	0.09	0.40	0.16	1.45	1.24	1.70	0.00	0.00
Cyanobacteria.id.1500	phylum	T2D	-0.02	0.09	-0.53	0.36	0.95	0.80	1.13	0.57	0.47
Firmicutes.id.1672	phylum	T2D	-0.06	0.09	0.04	0.26	0.95	0.80	1.13	0.58	0.00
Proteobacteria.id.2375	phylum	T2D	-0.13	0.08	-0.17	0.22	0.87	0.75	1.02	0.09	0.00
Tenericutes.id.3919	phylum	T2D	0.05	0.09	-0.02	0.24	1.04	0.88	1.23	0.65	0.00
Verrucomicrobia.id.3982	phylum	T2D	0.07	0.09	-0.03	0.29	1.06	0.90	1.26	0.49	0.00
Actinobacteria.id.419	class	T2D	0.05	0.09	-0.21	0.28	1.03	0.87	1.21	0.77	0.00
Alphaproteobacteria.id.2379	class	T2D	0.11	0.09	0.08	0.14	1.10	0.95	1.28	0.20	0.00
Bacilli.id.1673	class	T2D	-0.01	0.09	0.08	0.22	1.01	0.85	1.18	0.95	0.00
Bacteroidia.id.912	class	T2D	-0.16	0.09	-0.31	0.36	0.85	0.71	1.01	0.07	0.00
Betaproteobacteria.id.2867	class	T2D	-0.04	0.09	0.08	0.26	0.98	0.83	1.15	0.77	0.00
Clostridia.id.1859	class	T2D	0.00	0.09	0.03	0.23	1.01	0.85	1.19	0.95	0.00
Coriobacteriia.id.809	class	T2D	-0.07	0.08	-0.06	0.22	1.02	0.97	1.08	0.37	0.00
Deltaproteobacteria.id.3087	class	T2D	-0.21	0.09	-1.12	0.67	0.79	0.67	0.95	0.01	0.45
Erysipelotrichia.id.2147	class	T2D	-0.06	0.09	0.04	0.26	0.95	0.80	1.13	0.58	0.00
Gammaproteobacteria.id.3303	class	T2D	0.03	0.09	-0.34	0.34	1.01	0.85	1.20	0.94	0.07
Melainabacteria.id.1589	class	T2D	0.07	0.09	0.28	0.23	1.11	0.93	1.31	0.24	0.00
Mollicutes.id.3920	class	T2D	-0.30	0.08	-0.38	0.34	0.74	0.63	0.87	0.00	0.00
Negativicutes.id.2164	class	T2D	-0.70	0.10	-0.21	0.27	0.52	0.44	0.63	0.00	0.68
Verrucomicrobiae.id.4029	class	T2D	-0.12	0.10	0.00	0.20	0.91	0.76	1.08	0.27	0.00
Aeromonadales.id.3316	order	T2D	0.05	0.09	-0.02	0.24	1.04	0.88	1.23	0.65	0.00
Bacteroidales.id.913	order	T2D	0.07	0.09	-0.03	0.29	1.06	0.90	1.26	0.49	0.00
Bifidobacteriales.id.432	order	T2D	0.11	0.09	0.11	0.13	1.12	0.96	1.29	0.14	0.00
Burkholderiales.id.2874	order	T2D	0.12	0.08	-0.25	0.94	1.13	0.97	1.32	0.12	0.00
Clostridiales.id.1863	order	T2D	0.05	0.09	-0.21	0.28	1.03	0.87	1.21	0.77	0.00
Coriobacteriales.id.810	order	T2D	-0.22	0.08	-0.42	0.34	0.79	0.68	0.93	0.00	0.00

© 2021 Chen Z et al. *JAMA Network Open.*

Desulfovibrionales.id.3156	order	T2D	0.21	0.10	-0.04	0.25	1.19	1.00	1.43	0.05	0.00
Enterobacteriales.id.3468	order	T2D	0.32	0.08	0.10	0.14	1.31	1.14	1.50	0.00	0.45
Erysipelotrichales.id.2148	order	T2D	-0.02	0.09	0.16	0.15	1.02	0.88	1.19	0.77	0.06
Gastranaerophilales.id.1591	order	T2D	-0.14	0.09	-0.60	0.55	0.86	0.72	1.02	0.09	0.00
Lactobacillales.id.1800	order	T2D	-0.65	0.09	-0.38	0.30	0.53	0.45	0.63	0.00	0.00
MollicutesRF9.id.11579	order	T2D	-0.11	0.09	-0.47	0.39	0.88	0.74	1.05	0.14	0.00
Pasteurellales.id.3688	order	T2D	0.04	0.09	-0.47	0.53	1.03	0.86	1.22	0.77	0.00
Rhodospirillales.id.2667	order	T2D	-0.02	0.09	-0.05	0.28	0.98	0.82	1.16	0.78	0.00
Selenomonadales.id.2165	order	T2D	-0.14	0.09	-0.02	0.24	0.89	0.75	1.04	0.15	0.00
Verrucomicrobiales.id.4030	order	T2D	-0.21	0.08	0.18	0.26	0.84	0.72	0.97	0.02	0.51
Acidaminococcaceae.id.2166	family	T2D	0.32	0.09	0.42	0.16	1.41	1.21	1.64	0.00	0.00
Alcaligenaceae.id.2875	family	T2D	0.03	0.08	-386.85	40595.94	1.03	0.88	1.21	0.71	0.00
Bacteroidaceae.id.917	family	T2D	0.14	0.09	0.17	0.25	1.15	0.98	1.36	0.09	0.00
Bifidobacteriaceae.id.433	family	T2D	0.00	0.09	0.03	0.23	1.01	0.85	1.19	0.95	0.00
Christensenellaceae.id.1866	family	T2D	-0.20	0.14	-0.10	0.36	0.83	0.64	1.06	0.13	0.00
Clostridiaceae1.id.1869	family	T2D	-0.22	0.08	-0.15	0.28	0.81	0.70	0.94	0.01	0.00
ClostridialesvadinBB60group.id.11286	family	T2D	-0.34	0.09	0.24	0.18	0.80	0.69	0.94	0.01	0.88
Coriobacteriaceae.id.811	family	T2D	0.14	0.10	-0.14	0.28	1.11	0.92	1.33	0.27	0.00
Desulfovibrionaceae.id.3169	family	T2D	0.22	0.10	0.18	0.14	1.23	1.05	1.45	0.01	0.00
Enterobacteriaceae.id.3469	family	T2D	-0.24	0.10	0.16	0.21	0.85	0.71	1.01	0.07	0.65
Enterococcaceae.id.1828	family	T2D	-0.10	0.09	0.11	0.26	0.92	0.78	1.09	0.34	0.00
Erysipelotrichaceae.id.2149	family	T2D	-0.35	0.09	-0.22	0.30	0.71	0.60	0.85	0.00	0.00
FamilyXIII.id.1957	family	T2D	-0.19	0.09	-1.11	0.55	0.81	0.68	0.96	0.01	0.63
Lachnospiraceae.id.1987	family	T2D	-0.03	0.09	0.01	0.29	0.97	0.82	1.15	0.75	0.00
Lactobacillaceae.id.1836	family	T2D	0.30	0.10	-0.38	0.40	1.30	1.08	1.57	0.01	0.63
Pasteurellaceae.id.3689	family	T2D	0.14	0.08	-2.49	3.85	1.15	0.99	1.34	0.07	0.00
Peptococcaceae.id.2024	family	T2D	0.03	0.09	-0.11	0.26	1.01	0.86	1.19	0.90	0.00
Peptostreptococcaceae.id.2042	family	T2D	0.00	0.09	0.03	0.23	1.01	0.85	1.19	0.95	0.00
Porphyromonadaceae.id.943	family	T2D	-0.10	0.09	-0.04	0.25	0.91	0.77	1.07	0.25	0.00
Prevotellaceae.id.960	family	T2D	0.05	0.09	-3.15	4.55	1.05	0.88	1.24	0.60	0.00
Rhodospirillaceae.id.2717	family	T2D	-0.17	0.09	-0.01	0.24	0.86	0.73	1.01	0.07	0.00
Rikenellaceae.id.967	family	T2D	-0.23	0.09	-0.09	0.27	0.81	0.69	0.95	0.01	0.00
Ruminococcaceae.id.2050	family	T2D	0.03	0.09	-0.34	0.34	1.01	0.85	1.20	0.94	0.07

© 2021 Chen Z et al. *JAMA Network Open.*

Streptococcaceae.id.1850	family	T2D	-0.14	0.09	-0.47	0.41	0.86	0.72	1.03	0.09	0.00
Succinivibrionaceae.id.3326	family	T2D	0.07	0.09	0.26	0.23	1.10	0.93	1.30	0.26	0.00
Veillonellaceae.id.2172	family	T2D	0.22	0.10	-0.14	0.28	1.20	1.00	1.44	0.04	0.33
Verrucomicrobiaceae.id.4036	family	T2D	0.12	0.09	-0.81	0.45	1.09	0.91	1.30	0.37	0.76
unknownfamily.id.1000001214	family	T2D	0.01	0.09	-0.25	0.44	1.00	0.84	1.19	0.98	0.00
unknownfamily.id.1000005471	family	T2D	-0.20	0.11	-0.37	0.56	0.82	0.67	1.00	0.05	0.00
unknownfamily.id.987	family	T2D	0.06	0.08	-1.46	1.47	1.05	0.90	1.24	0.52	0.05
Bacteroidespectinophilusgroup.id.14371	genus	T2D	-0.28	0.08	-0.36	0.34	0.75	0.64	0.88	0.00	0.00
Eubacteriumcoprostanoligenesgroup.id.11375	genus	T2D	-0.72	0.10	-0.21	0.27	0.52	0.43	0.62	0.00	0.67
Eubacteriumeligensgroup.id.14372	genus	T2D	0.07	0.09	0.13	0.21	1.08	0.92	1.27	0.36	0.00
Eubacteriumhalliigroup.id.11338	genus	T2D	0.14	0.10	0.10	0.24	1.14	0.95	1.36	0.15	0.00
Eubacteriumrectalegroup.id.14374	genus	T2D	-0.15	0.09	-0.26	0.33	0.86	0.72	1.02	0.08	0.00
Eubacteriumruminantiumgroup.id.11340	genus	T2D	0.08	0.10	-0.57	0.33	1.03	0.86	1.23	0.77	0.72
Eubacteriumventriosumgroup.id.11341	genus	T2D	0.02	0.09	-0.13	0.33	1.01	0.85	1.20	0.91	0.00
Eubacteriumxylanophilumgroup.id.14375	genus	T2D	0.09	0.09	0.19	0.24	1.11	0.94	1.31	0.21	0.00
Ruminococcusgauvreauiigroup.id.11342	genus	T2D	-0.07	0.09	-0.40	0.30	0.91	0.77	1.07	0.27	0.13
Ruminococcusgnavusgroup.id.14376	genus	T2D	0.14	0.08	-0.70	0.89	1.14	0.97	1.35	0.11	0.00
Ruminococcustorquesgroup.id.14377	genus	T2D	0.16	0.08	0.15	0.14	1.17	1.02	1.35	0.03	0.00
Acidaminococcus.id.2167	genus	T2D	0.12	0.08	-3.65	8.88	1.12	0.96	1.31	0.14	0.00
Adlercreutzia.id.812	genus	T2D	-0.04	0.09	-0.48	0.37	0.94	0.79	1.11	0.46	0.24
Akkermansia.id.4037	genus	T2D	-0.02	0.09	0.19	0.15	1.04	0.89	1.21	0.63	0.31
Alistipes.id.968	genus	T2D	-0.13	0.09	-0.11	0.31	0.88	0.74	1.04	0.14	0.00
Alloprevotella.id.961	genus	T2D	-0.14	0.08	-0.09	0.26	0.87	0.74	1.02	0.08	0.00
Anaerostipes.id.1991	genus	T2D	-0.15	0.09	-0.95	0.46	0.84	0.71	1.00	0.04	0.66
Anaerotruncus.id.2054	genus	T2D	-0.21	0.09	-0.14	0.27	0.81	0.69	0.96	0.01	0.00
Bacteroides.id.918	genus	T2D	-0.11	0.09	0.28	0.17	0.97	0.83	1.12	0.65	0.76
Barnesiella.id.944	genus	T2D	-0.02	0.09	0.16	0.15	1.03	0.89	1.20	0.71	0.00
Bifidobacterium.id.436	genus	T2D	0.13	0.09	-0.07	0.25	1.12	0.95	1.31	0.17	0.00
Blautia.id.1992	genus	T2D	-0.57	0.10	-0.12	0.24	0.60	0.51	0.72	0.00	0.67
Butyricicoccus.id.2055	genus	T2D	-0.13	0.09	0.10	0.21	0.91	0.78	1.07	0.25	0.02
Butyricimonas.id.945	genus	T2D	0.16	0.09	0.28	0.18	1.20	1.02	1.41	0.03	0.00
Butyrivibrio.id.1993	genus	T2D	-0.24	0.09	-0.34	0.37	0.78	0.66	0.93	0.00	0.00
Catenibacterium.id.2153	genus	T2D	-0.07	0.09	0.27	0.16	1.01	0.86	1.17	0.93	0.69

© 2021 Chen Z et al. *JAMA Network Open.*

ChristensenellaceaeR7group.id.11283	genus	T2D	-0.27	0.08	0.17	0.22	0.81	0.69	0.95	0.01	0.71
Clostridiumsensustricto1.id.1873	genus	T2D	-0.25	0.09	0.03	0.24	0.81	0.69	0.95	0.01	0.17
Collinsella.id.815	genus	T2D	0.08	0.09	0.01	0.20	1.07	0.91	1.25	0.42	0.00
Coprococcus1.id.11301	genus	T2D	-0.09	0.09	-0.59	0.49	0.90	0.76	1.08	0.26	0.00
Coprococcus2.id.11302	genus	T2D	0.04	0.09	-0.31	0.41	1.03	0.86	1.22	0.77	0.00
Coprococcus3.id.11303	genus	T2D	0.31	0.08	0.07	0.17	1.30	1.13	1.50	0.00	0.41
Desulfovibrio.id.3173	genus	T2D	-0.13	0.09	-0.67	0.43	0.86	0.73	1.02	0.08	0.34
Dialister.id.2183	genus	T2D	0.01	0.09	-704.30	70799.55	1.01	0.84	1.20	0.93	0.00
Dorea.id.1997	genus	T2D	-0.05	0.09	-0.34	0.69	0.95	0.79	1.14	0.56	0.00
Eggerthella.id.819	genus	T2D	0.04	0.08	-0.20	0.54	1.04	0.88	1.22	0.65	0.00
Eisenbergiella.id.11304	genus	T2D	0.05	0.09	-0.08	0.28	1.04	0.87	1.23	0.67	0.00
Enterococcus.id.1831	genus	T2D	0.15	0.08	-9.09	6.52	1.16	0.99	1.36	0.08	0.50
Enterorhabdus.id.820	genus	T2D	-0.21	0.10	-0.02	0.28	0.82	0.69	0.98	0.03	0.00
Erysipelatoclostridium.id.11381	genus	T2D	-0.09	0.09	-0.37	0.40	0.90	0.76	1.08	0.25	0.00
ErysipelotrichaceaeUCG003.id.11384	genus	T2D	0.04	0.09	-0.15	0.34	1.03	0.87	1.22	0.72	0.00
Faecalibacterium.id.2057	genus	T2D	-0.07	0.09	0.18	0.19	0.98	0.83	1.16	0.83	0.33
FamilyXIIIAD3011group.id.11293	genus	T2D	-0.02	0.09	-0.65	0.58	0.97	0.81	1.15	0.71	0.14
FamilyXIIIUCG001.id.11294	genus	T2D	-0.20	0.09	-0.90	0.60	0.81	0.67	0.96	0.02	0.24
Fusicatenibacter.id.11305	genus	T2D	0.06	0.08	-366.30	30802.74	1.06	0.90	1.24	0.49	0.00
Haemophilus.id.3698	genus	T2D	-0.02	0.09	0.01	0.18	0.98	0.84	1.15	0.82	0.00
Holdemanella.id.11393	genus	T2D	0.03	0.09	-0.33	0.51	1.02	0.86	1.21	0.79	0.00
Intestinibacter.id.11345	genus	T2D	-0.10	0.10	-0.33	0.76	0.90	0.74	1.10	0.31	0.00
Intestinimonas.id.2062	genus	T2D	-0.05	0.10	-0.97	2.57	0.95	0.79	1.15	0.62	0.00
Lachnoclostridium.id.11308	genus	T2D	-0.05	0.09	0.07	0.20	0.98	0.83	1.15	0.77	0.00
Lachnospira.id.2004	genus	T2D	-0.66	0.09	-1.19	0.61	0.51	0.43	0.61	0.00	0.00
LachnospiraceaeFCS020group.id.11314	genus	T2D	-0.01	0.09	-0.38	0.34	0.96	0.81	1.15	0.68	0.10
LachnospiraceaeND3007group.id.11317	genus	T2D	-0.23	0.09	0.30	0.15	0.91	0.79	1.06	0.22	0.89
LachnospiraceaeNK4A136group.id.11319	genus	T2D	-0.30	0.09	-0.04	0.25	0.77	0.65	0.91	0.00	0.00
LachnospiraceaeUCG001.id.11321	genus	T2D	-0.16	0.09	-0.05	0.26	0.86	0.73	1.01	0.07	0.00
LachnospiraceaeUCG004.id.11324	genus	T2D	-0.30	0.09	-0.44	0.39	0.73	0.62	0.87	0.00	0.00
LachnospiraceaeUCG010.id.11330	genus	T2D	-0.21	0.08	-0.25	0.31	0.81	0.69	0.94	0.01	0.00
Lactobacillus.id.1837	genus	T2D	-0.17	0.09	-0.18	0.30	0.84	0.71	1.00	0.06	0.00
Marvinbryantia.id.2005	genus	T2D	0.06	0.09	0.02	0.25	1.06	0.90	1.25	0.49	0.00

© 2021 Chen Z et al. *JAMA Network Open.*

Megamonas.id.2184	genus	T2D	-0.31	0.09	-0.20	0.31	0.74	0.63	0.87	0.00	0.00
Megasphaera.id.2185	genus	T2D	-0.18	0.10	0.00	0.22	0.86	0.72	1.03	0.10	0.00
Mitsuokella.id.2186	genus	T2D	-0.33	0.10	-0.11	0.27	0.74	0.62	0.88	0.00	0.00
Odoribacter.id.952	genus	T2D	-0.03	0.09	-0.31	0.31	0.95	0.80	1.12	0.53	0.00
Olsenella.id.822	genus	T2D	-0.17	0.09	0.02	0.24	0.86	0.74	1.01	0.07	0.00
Oscillospira.id.2064	genus	T2D	-0.21	0.09	0.09	0.22	0.85	0.72	0.99	0.04	0.39
Parabacteroides.id.954	genus	T2D	-0.09	0.09	0.15	0.22	0.95	0.81	1.11	0.51	0.03
Paraprevotella.id.962	genus	T2D	-0.05	0.09	0.10	0.17	0.99	0.84	1.16	0.86	0.00
Parasutterella.id.2892	genus	T2D	-0.08	0.09	0.13	0.19	0.96	0.82	1.13	0.62	0.08
Peptococcus.id.2037	genus	T2D	-0.07	0.09	-0.40	0.38	0.91	0.77	1.09	0.30	0.00
Phascolarctobacterium.id.2168	genus	T2D	0.33	0.09	0.42	0.16	1.42	1.22	1.65	0.00	0.00
Prevotella2.id.11180	genus	T2D	-0.01	0.09	0.31	0.21	1.05	0.89	1.23	0.59	0.50
Prevotella7.id.11182	genus	T2D	-0.10	0.11	-290.23	22660.60	0.90	0.73	1.11	0.33	0.00
Prevotella9.id.11183	genus	T2D	-0.06	0.09	-0.70	0.65	0.93	0.77	1.11	0.41	0.00
PrevotellaceaeNK3B31group.id.11185	genus	T2D	-0.33	0.10	0.24	0.11	0.94	0.81	1.09	0.40	0.93
PrevotellaceaeUCG001.id.11186	genus	T2D	-0.19	0.09	0.07	0.19	0.87	0.74	1.03	0.11	0.29
RikenellaceaeRC9gutgroup.id.11191	genus	T2D	0.10	0.08	-132.55	10703.92	1.11	0.94	1.31	0.23	0.00
Romboutsia.id.11347	genus	T2D	0.12	0.08	0.17	0.13	1.15	1.00	1.32	0.05	0.00
Roseburia.id.2012	genus	T2D	0.03	0.08	-386.83	40583.02	1.03	0.88	1.21	0.71	0.00
Ruminiclostridium5.id.11355	genus	T2D	-0.02	0.09	-0.53	0.36	0.95	0.80	1.13	0.57	0.47
Ruminiclostridium6.id.11356	genus	T2D	0.07	0.09	0.28	0.23	1.11	0.93	1.31	0.24	0.00
Ruminiclostridium9.id.11357	genus	T2D	-0.06	0.09	0.04	0.26	0.95	0.80	1.13	0.58	0.00
RuminococcaceaeNK4A214group.id.11358	genus	T2D	-0.13	0.08	-0.17	0.22	0.87	0.75	1.02	0.09	0.00
RuminococcaceaeUCG002.id.11360	genus	T2D	0.05	0.09	-0.02	0.24	1.04	0.88	1.23	0.65	0.00
RuminococcaceaeUCG003.id.11361	genus	T2D	0.07	0.09	-0.02	0.29	1.06	0.90	1.26	0.48	0.00
RuminococcaceaeUCG004.id.11362	genus	T2D	0.11	0.09	0.11	0.13	1.12	0.96	1.29	0.14	0.00
RuminococcaceaeUCG005.id.11363	genus	T2D	0.05	0.09	-0.21	0.28	1.03	0.87	1.21	0.77	0.00
RuminococcaceaeUCG008.id.11365	genus	T2D	-0.01	0.09	0.08	0.22	1.01	0.85	1.19	0.94	0.00
RuminococcaceaeUCG010.id.11367	genus	T2D	0.36	0.09	0.40	0.16	1.45	1.24	1.70	0.00	0.00
RuminococcaceaeUCG013.id.11370	genus	T2D	-0.13	0.09	-0.32	0.37	0.87	0.73	1.04	0.13	0.00
RuminococcaceaeUCG014.id.11371	genus	T2D	-0.02	0.09	0.16	0.15	1.02	0.88	1.19	0.77	0.06
Ruminococcus1.id.11373	genus	T2D	-0.02	0.09	-0.05	0.28	0.98	0.83	1.17	0.82	0.00
Ruminococcus2.id.11374	genus	T2D	-0.04	0.09	0.08	0.26	0.98	0.83	1.15	0.77	0.00

© 2021 Chen Z et al. *JAMA Network Open.*

Sellimonas.id.14369	genus	T2D	0.00	0.09	0.03	0.23	1.01	0.85	1.19	0.95	0.00
Senegalimassilia.id.11160	genus	T2D	0.09	0.09	0.23	0.24	1.11	0.94	1.32	0.20	0.00
Slackia.id.825	genus	T2D	-0.02	0.09	-0.51	0.35	0.95	0.80	1.13	0.56	0.45
Streptococcus.id.1853	genus	T2D	0.01	0.09	0.03	0.26	1.01	0.86	1.19	0.89	0.00
Subdoligranulum.id.2070	genus	T2D	-0.01	0.09	-0.03	0.23	0.98	0.83	1.16	0.84	0.00
Succiniclasticum.id.2169	genus	T2D	0.07	0.09	0.08	0.14	1.07	0.92	1.24	0.36	0.00
Sutterella.id.2896	genus	T2D	-0.16	0.09	-0.31	0.36	0.85	0.71	1.01	0.07	0.00
Terrisporobacter.id.11348	genus	T2D	0.00	0.09	0.03	0.23	1.00	0.85	1.18	0.99	0.00
Turicibacter.id.2162	genus	T2D	-0.01	0.09	0.08	0.22	1.01	0.85	1.19	0.94	0.00
Tyzzerella3.id.11335	genus	T2D	-0.13	0.09	-0.32	0.37	0.87	0.73	1.04	0.13	0.00
Veillonella.id.2198	genus	T2D	-0.07	0.09	-0.81	1.00	0.92	0.77	1.11	0.39	0.00
unknowngenus.id.1000000073	genus	T2D	-0.12	0.10	0.00	0.20	0.91	0.76	1.08	0.27	0.00
unknowngenus.id.1000001215	genus	T2D	-0.01	0.09	0.08	0.22	1.01	0.85	1.19	0.94	0.00
unknowngenus.id.1000005472	genus	T2D	-0.13	0.09	-0.32	0.37	0.87	0.73	1.04	0.13	0.00
unknowngenus.id.1000005479	genus	T2D	-0.06	0.09	-0.75	0.73	0.93	0.78	1.11	0.42	0.00
unknowngenus.id.1000013899	genus	T2D	-0.07	0.09	-0.81	1.00	0.92	0.77	1.11	0.39	0.00
unknowngenus.id.2071	genus	T2D	-0.24	0.10	0.12	0.19	0.85	0.72	1.01	0.06	0.66
unknowngenus.id.2755	genus	T2D	-0.02	0.09	-0.07	0.30	0.97	0.82	1.16	0.75	0.00
unknowngenus.id.826	genus	T2D	-0.03	0.09	-0.27	0.31	0.96	0.81	1.13	0.60	0.00
unknowngenus.id.964	genus	T2D	-0.03	0.09	-233.51	24550.24	0.97	0.80	1.16	0.72	0.00

Taxa in RS only	Clade	Phenotype	Beta_RS	Se_RS
family.BacteroidalesS247group.id.11173	family	T2D	-0.06	0.09
family.Veillonellaceae.id.2172	family	T2D	0.14	0.09
genus..Bacteroidespectinophilusgroup.id.1437	family	T2D	-0.20	0.14
genus.Enterobacter.id.3502	genus	T2D	0.00	0.10
genus.EscherichiaShigella.id.3504	genus	T2D	0.03	0.09
genus..Eubacteriumcoprostanoligenesgroup.id	genus	T2D	-0.22	0.08
genus..Eubacteriumeligensgroup.id.14372	genus	T2D	-0.34	0.09
genus..Eubacteriumhalliigroup.id.11338	genus	T2D	0.14	0.10
genus..Eubacteriumoxidoreducensgroup.id.113	genus	T2D	-0.19	0.09

© 2021 Chen Z et al. *JAMA Network Open.*

genus..Eubacteriumxylanophilumgroup.id.1437	genus	T2D	-0.35	0.09
genus.Hungatella.id.11306	genus	T2D	0.07	0.09
genus.Klebsiella.id.3507	genus	T2D	0.19	0.07
genus.LachnospiraceaeNC2004group.id.11316	genus	T2D	-0.23	0.09
genus.LachnospiraceaeUCG008.id.11328	genus	T2D	-0.08	0.09
genus.unknowngenus.id.1868	genus	T2D	-0.25	0.09
**Taxa in LLD only**	**Clade**	**Phenotype**	**Beta_LLD**	**Se_LLD**
domain.Archaea.id.2	Clade	T2D	0.06	0.22
phylum.Euryarchaeota.id.55	domain	T2D	0.06	0.22
phylum.Lentisphaerae.id.2238	phylum	T2D	-0.04	0.25
order.Actinomycetales.id.420	phylum	T2D	0.12	0.15
order.Mycoplasmatales.id.3946	order	T2D	-0.42	53.21
order.NB1.n.id.3953	order	T2D	0.06	0.18
order.Pseudomonadales.id.3709	order	T2D	-0.22	3.03
order.Spirochaetales.id.3870	order	T2D	-17.98	1882.17
order.Synergistales.id.3900	order	T2D	-1.18	1.67
order.Thermoplasmatales.id.220	order	T2D	0.15	0.09
order.unknownorder.id.1000000003	order	T2D	-0.33	0.70
order.Verrucomicrobiales.id.4030	order	T2D	0.03	0.23
order.Victivallales.id.2254	order	T2D	-0.04	0.25
order.Xanthomonadales.id.3786	order	T2D	-23.17	2531.04
class.Spirochaetes.id.3856	order	T2D	-17.98	1882.17
class.Synergistia.id.3899	class	T2D	-1.18	1.67
class.Thermoplasmata.id.210	class	T2D	0.15	0.09
family.Actinomycetaceae.id.421	class	T2D	0.12	0.15
family.BacteroidalesS24.7group.id.11173	family	T2D	-0.75	0.73
family.Christensenellaceae.id.1866	family	T2D	-0.38	0.34
family.FamilyXI.id.1936	family	T2D	-0.60	0.88
family.unknownfamily.id.1000000004	family	T2D	-0.33	0.70
family.unknownfamily.id.1000006161	family	T2D	0.06	0.18
family.unknownfamily.id.1855	family	T2D	0.16	0.08
family.vadinBE97.id.14446	family	T2D	-0.88	1.74
family.Veillonellaceae.id.2172	family	T2D	0.17	0.25

© 2021 Chen Z et al. *JAMA Network Open.*

family.Xanthomonadaceae.id.3799	family	T2D	-23.17	2531.04
genus..Clostridiuminnocuumgroup.id.14397	family	T2D	0.20	0.12
genus..Eubacteriumbrachygroup.id.11296	family	T2D	0.32	0.13
genus.Abiotrophia.id.1803	genus	T2D	-13.05	1002.76
genus.Actinomyces.id.423	genus	T2D	0.07	0.16
genus.Actinotignum.id.11137	genus	T2D	-14.86	1183.52
genus.Allisonella.id.2174	genus	T2D	-0.19	0.35
genus.Arcanobacterium.id.424	genus	T2D	-16.17	1632.63
genus.Asaccharobacter.id.813	genus	T2D	0.10	0.23
genus.Bilophila.id.3170	genus	T2D	0.18	0.22
genus.CandidatusMethanogranum.id.11110	genus	T2D	-4.17	431.66
genus.Catenisphaera.id.14395	genus	T2D	-797.38	71752.59
genus.Cellulosilyticum.id.1995	genus	T2D	-82.67	6750.72
genus.Cetobacterium.id.2209	genus	T2D	-0.44	53.21
genus.Cloacibacillus.id.3908	genus	T2D	-0.58	0.91
genus.Coprobacillus.id.2154	genus	T2D	-0.78	1.59
genus.CoriobacteriaceaeUCG.002.id.11158	genus	T2D	-0.40	0.58
genus.CoriobacteriaceaeUCG.003.id.11159	genus	T2D	-287.68	30911.31
genus.Denitrobacterium.id.818	genus	T2D	0.25	0.09
genus.dgA.11gutgroup.id.978	genus	T2D	-0.47	53.21
genus.Dielma.id.11380	genus	T2D	-0.30	1.72
genus.Epulopiscium.id.1998	genus	T2D	-1.76	2.84
genus.ErysipelotrichaceaeUCG006.id.11386	genus	T2D	-6.67	599.88
genus.ErysipelotrichaceaeUCG009.id.11389	genus	T2D	-0.47	53.21
genus.Escherichia.Shigella.id.3504	genus	T2D	0.09	0.13
genus.Eubacterium.id.1932	genus	T2D	0.26	0.24
genus.Faecalicoccus.id.11391	genus	T2D	-70.76	5178.86
genus.Faecalitalea.id.11392	genus	T2D	0.14	0.09
genus.Flavonifractor.id.2059	genus	T2D	-0.16	0.49
genus.Gardnerella.id.437	genus	T2D	-15.95	1515.01
genus.Gordonibacter.id.821	genus	T2D	-0.27	0.42
genus.Hafnia.Obesumbacterium.id.14636	genus	T2D	0.33	0.47
genus.Howardella.id.2000	genus	T2D	-0.49	0.39

© 2021 Chen Z et al. *JAMA Network Open.*

genus.LachnospiraceaeNK3A20group.id.11318	genus	T2D	-2.78	336.53
genus.Lactococcus.id.1851	genus	T2D	-0.39	0.71
genus.Lautropia.id.2905	genus	T2D	-46.78	5123.76
genus.Leuconostoc.id.1841	genus	T2D	-0.77	0.73
genus.Methanobrevibacter.id.123	genus	T2D	0.11	0.22
genus.Methanosphaera.id.124	genus	T2D	-0.35	0.46
genus.Mogibacterium.id.1960	genus	T2D	-1.50	1.55
genus.Morganella.id.3512	genus	T2D	-0.37	53.21
genus.Porphyromonas.id.956	genus	T2D	-1.36	1.78
genus.Prevotella1.id.11179	genus	T2D	-1.02	95.11
genus.PrevotellaceaeUCG003.id.11187	genus	T2D	-59.54	6966.22
genus.PrevotellaceaeUCG004.id.11188	genus	T2D	-4.99	486.22
genus.Pseudomonas.id.3723	genus	T2D	-1019.69	75983.98
genus.Rikenella.id.973	genus	T2D	-15.54	1318.59
genus.RuminococcaceaeNK4A214group.id.113	genus	T2D	-0.44	0.39
genus.RuminococcaceaeUCG005.id.11363	genus	T2D	-0.20	0.31
genus.RuminococcaceaeUCG008.id.11365	genus	T2D	0.00	0.22
genus.RuminococcaceaeUCG010.id.11367	genus	T2D	-0.11	0.27
genus.Sarcina.id.1896	genus	T2D	-61.45	5551.30
genus.Solobacterium.id.2161	genus	T2D	-3.46	5.10
genus.Staphylococcus.id.1780	genus	T2D	-0.03	0.29
genus.Stenotrophomonas.id.3818	genus	T2D	-0.46	53.21
genus.Succinivibrio.id.3331	genus	T2D	-242.11	22249.13
genus.Synergistes.id.3913	genus	T2D	-24.53	2041.02
genus.Syntrophococcus.id.2016	genus	T2D	-0.85	92.21
genus.Treponema2.id.11577	genus	T2D	-15.37	1902.38
genus.Tyzzerella4.id.11336	genus	T2D	-4.55	8.93
genus.unknowngenus.id.1000000005	genus	T2D	-0.33	0.70
genus.unknowngenus.id.1000027565	genus	T2D	-0.88	1.74
genus.unknowngenus.id.1000043796	genus	T2D	0.16	0.08
genus.unknowngenus.id.1854	genus	T2D	-41.58	2981.31
genus.unknowngenus.id.2163	genus	T2D	0.15	0.10
genus.unknowngenus.id.3332	genus	T2D	-32.60	2665.47

© 2021 Chen Z et al. *JAMA Network Open.*

genus.unknowngenus.id.441	genus	T2D	-0.11	0.37
genus.Ureaplasma.id.3951	genus	T2D	-0.42	53.21
genus.Varibaculum.id.427	genus	T2D	0.23	0.14
genus.Veillonella.id.2198	genus	T2D	0.17	0.13
genus.Weissella.id.1843	genus	T2D	0.06	0.14

© 2021 Chen Z et al. *JAMA Network Open.*

Supplementary Table 3 - Associations of taxa and type 2 diabetes

Effect estimates in RS and LLD were calculated using logistic regression; pooled estimates were calculated based on a mixed-effect meta-analysis.

Model 2: Adjusted for age, sex, Time in mail (RS), Batch (RS), smoking, education level (RS), physical activity, alcohol intake, and total energy intakeI.

Abbreviation, RS, Rotterdam Study, LLD, Lifelines-Deep Study. P<0.0005 indicates statistical significance

Overlapping taxa in RS and LLD	Clade	Phenotype	Beta_RS	Se_RS	Beta_LLD	Se_LLD	OR_Meta	Lower95%CI_Meta	Upper95%CI_Meta	P_Meta	I^2
Bacteria.id.3	domain	T2D	0.11	0.12	0.26	0.23	1.15	0.94	1.41	0.18	0.00
Actinobacteria.id.400	phylum	T2D	-0.05	0.11	-0.06	0.31	0.95	0.77	1.17	0.61	0.00
Bacteroidetes.id.905	phylum	T2D	0.36	0.11	0.36	0.17	1.43	1.20	1.72	0.00	0.00
Cyanobacteria.id.1500	phylum	T2D	-0.08	0.11	-0.57	0.35	0.88	0.72	1.09	0.25	0.44
Firmicutes.id.1672	phylum	T2D	-0.02	0.12	-0.07	0.26	0.98	0.79	1.20	0.82	0.00
Proteobacteria.id.2375	phylum	T2D	-0.19	0.10	-0.15	0.22	0.84	0.70	1.00	0.05	0.00
Tenericutes.id.3919	phylum	T2D	-0.07	0.11	0.14	0.24	0.97	0.79	1.19	0.77	0.00
Verrucomicrobia.id.3982	phylum	T2D	0.04	0.11	-0.04	0.32	1.03	0.84	1.27	0.76	0.00
Actinobacteria.id.419	class	T2D	0.00	0.11	-0.18	0.28	0.98	0.80	1.20	0.83	0.00
Alphaproteobacteria.id.2379	class	T2D	0.08	0.11	0.11	0.15	1.10	0.92	1.31	0.31	0.00
Bacilli.id.1673	class	T2D	-0.11	0.12	-0.01	0.25	0.91	0.74	1.13	0.40	0.00
Bacteroidia.id.912	class	T2D	-0.23	0.12	-0.27	0.38	0.79	0.64	0.99	0.04	0.00
Betaproteobacteria.id.2867	class	T2D	-0.13	0.11	0.09	0.28	0.91	0.74	1.11	0.33	0.00
Clostridia.id.1859	class	T2D	0.00	0.11	0.01	0.23	1.00	0.82	1.22	0.98	0.00
Coriobacteriia.id.809	class	T2D	-0.06	0.10	-0.07	0.22	1.02	0.97	1.08	0.39	0.00
Deltaproteobacteria.id.3087	class	T2D	-0.13	0.11	-1.17	0.69	0.85	0.69	1.06	0.15	0.54
Erysipelotrichia.id.2147	class	T2D	-0.01	0.12	-0.09	0.27	0.97	0.79	1.20	0.81	0.00
Gammaproteobacteria.id.3303	class	T2D	-0.01	0.12	-0.42	0.34	0.95	0.76	1.17	0.61	0.24
Melainabacteria.id.1589	class	T2D	0.07	0.12	0.25	0.23	1.11	0.90	1.36	0.32	0.00
Mollicutes.id.3920	class	T2D	-0.36	0.10	-0.33	0.34	0.70	0.58	0.85	0.00	0.00
Negativicutes.id.2164	class	T2D	-0.83	0.13	-0.14	0.25	0.50	0.40	0.62	0.00	0.83
Verrucomicrobiae.id.4029	class	T2D	-0.19	0.13	0.03	0.23	0.87	0.70	1.08	0.22	0.00
Aeromonadales.id.3316	order	T2D	-0.07	0.11	0.14	0.24	0.97	0.79	1.19	0.77	0.00
Bacteroidales.id.913	order	T2D	0.04	0.11	-0.04	0.32	1.03	0.84	1.27	0.75	0.00
Bifidobacteriales.id.432	order	T2D	0.09	0.11	0.12	0.14	1.11	0.93	1.32	0.24	0.00
Burkholderiales.id.2874	order	T2D	0.17	0.09	-0.48	1.13	1.18	0.99	1.41	0.06	0.00
Clostridiales.id.1863	order	T2D	0.00	0.11	-0.18	0.28	0.98	0.80	1.20	0.83	0.00

© 2021 Chen Z et al. *JAMA Network Open.*

Coriobacteriales.id.810	order	T2D	-0.25	0.10	-0.40	0.34	0.77	0.64	0.93	0.01	0.00
Desulfovibrionales.id.3156	order	T2D	0.16	0.12	-0.07	0.24	1.12	0.91	1.39	0.27	0.00

© 2021 Chen Z et al. *JAMA Network Open.*

Enterobacteriales.id.3468	order	T2D	0.27	0.10	0.01	0.14	1.20	1.02	1.40	0.03	0.59
Erysipelotrichales.id.2148	order	T2D	-0.09	0.12	0.18	0.16	1.01	0.84	1.22	0.89	0.45
Gastranaerophilales.id.1591	order	T2D	-0.12	0.11	-0.59	0.55	0.87	0.70	1.08	0.21	0.00
Lactobacillales.id.1800	order	T2D	-0.70	0.11	-0.33	0.29	0.52	0.43	0.64	0.00	0.27
MollicutesRF9.id.11579	order	T2D	-0.16	0.11	-0.56	0.39	0.83	0.67	1.02	0.08	0.00
Pasteurellales.id.3688	order	T2D	0.05	0.11	-0.34	0.47	1.03	0.83	1.27	0.81	0.00
Rhodospirillales.id.2667	order	T2D	-0.05	0.11	-0.03	0.28	0.95	0.78	1.17	0.65	0.00
Selenomonadales.id.2165	order	T2D	-0.16	0.11	-0.02	0.25	0.87	0.71	1.06	0.17	0.00
Verrucomicrobiales.id.4030	order	T2D	-0.23	0.10	0.22	0.27	0.84	0.70	1.01	0.06	0.58
Acidaminococcaceae.id.2166	family	T2D	0.34	0.11	0.41	0.16	1.43	1.20	1.71	0.00	0.00
Alcaligenaceae.id.2875	family	T2D	0.04	0.10	-399.26	40432.95	1.04	0.84	1.27	0.74	0.00
Bacteroidaceae.id.917	family	T2D	-0.03	0.11	0.18	0.26	1.00	0.82	1.23	0.97	0.00
Bifidobacteriaceae.id.433	family	T2D	0.00	0.11	0.01	0.23	1.00	0.82	1.22	0.98	0.00
Christensenellaceae.id.1866	family	T2D	-0.81	0.57	-0.15	0.44	0.67	0.34	1.33	0.25	0.00
Clostridiaceae1.id.1869	family	T2D	-0.30	0.10	-0.20	0.29	0.75	0.63	0.90	0.00	0.00
ClostridialesvadinBB60group.id.11286	family	T2D	-0.33	0.11	0.23	0.18	0.84	0.70	1.01	0.07	0.86
Coriobacteriaceae.id.811	family	T2D	0.08	0.12	-0.10	0.27	1.06	0.86	1.30	0.61	0.00
Desulfovibrionaceae.id.3169	family	T2D	0.13	0.12	0.09	0.15	1.12	0.93	1.35	0.22	0.00
Enterobacteriaceae.id.3469	family	T2D	-0.18	0.12	0.17	0.20	0.92	0.75	1.12	0.39	0.56
Enterococcaceae.id.1828	family	T2D	-0.11	0.11	0.07	0.23	0.92	0.76	1.13	0.43	0.00
Erysipelotrichaceae.id.2149	family	T2D	-0.40	0.12	-0.26	0.33	0.68	0.55	0.84	0.00	0.00
FamilyXIII.id.1957	family	T2D	-0.16	0.11	-1.01	0.53	0.82	0.67	1.02	0.07	0.59
Lachnospiraceae.id.1987	family	T2D	-0.05	0.11	-0.06	0.38	0.95	0.77	1.17	0.62	0.00
Lactobacillaceae.id.1836	family	T2D	0.26	0.12	-0.28	0.37	1.23	0.98	1.54	0.07	0.48
Pasteurellaceae.id.3689	family	T2D	0.13	0.09	-3.16	4.59	1.14	0.95	1.37	0.17	0.00
Peptococcaceae.id.2024	family	T2D	0.04	0.11	-0.02	0.26	1.04	0.85	1.26	0.73	0.00
Peptostreptococcaceae.id.2042	family	T2D	0.00	0.11	0.01	0.23	1.00	0.82	1.22	0.98	0.00
Porphyromonadaceae.id.943	family	T2D	-0.11	0.11	-0.05	0.26	0.90	0.74	1.10	0.31	0.00
Prevotellaceae.id.960	family	T2D	-0.03	0.12	-2.68	4.20	0.97	0.77	1.22	0.79	0.00
Rhodospirillaceae.id.2717	family	T2D	-0.05	0.11	0.02	0.23	0.96	0.79	1.17	0.70	0.00
Rikenellaceae.id.967	family	T2D	-0.33	0.11	-0.10	0.29	0.74	0.61	0.90	0.00	0.00
Ruminococcaceae.id.2050	family	T2D	-0.01	0.12	-0.42	0.34	0.95	0.76	1.17	0.61	0.24

© 2021 Chen Z et al. *JAMA Network Open.*

Streptococcaceae.id.1850	family	T2D	-0.15	0.11	-0.59	0.44	0.84	0.67	1.04	0.11	0.00
Succinivibrionaceae.id.3326	family	T2D	0.06	0.12	0.26	0.23	1.11	0.90	1.36	0.32	0.00

© 2021 Chen Z et al. *JAMA Network Open.*

Veillonellaceae.id.2172	family	T2D	0.28	0.12	-0.13	0.29	1.24	1.00	1.55	0.05	0.40
Verrucomicrobiaceae.id.4036	family	T2D	0.10	0.11	-0.62	0.44	1.06	0.86	1.31	0.59	0.59
unknownfamily.id.1000001214	family	T2D	0.06	0.11	-0.24	0.40	1.04	0.84	1.28	0.73	0.00
unknownfamily.id.1000005471	family	T2D	-0.15	0.12	-0.48	0.64	0.85	0.67	1.07	0.17	0.00
unknownfamily.id.987	family	T2D	0.08	0.10	-1.58	1.56	1.07	0.88	1.31	0.50	0.12
Bacteroidespectinophilusgroup.id.14371	genus	T2D	-0.34	0.10	-0.32	0.35	0.71	0.58	0.86	0.00	0.00
Eubacteriumcoprostanoligenesgroup.id.1137	genus	T2D	-0.85	0.13	-0.14	0.26	0.49	0.39	0.61	0.00	0.83
Eubacteriumeligensgroup.id.14372	genus	T2D	0.08	0.11	0.26	0.22	1.12	0.92	1.36	0.25	0.00
Eubacteriumhalliigroup.id.11338	genus	T2D	0.06	0.12	0.12	0.24	1.08	0.88	1.32	0.47	0.00
Eubacteriumrectalegroup.id.14374	genus	T2D	-0.19	0.11	-0.22	0.35	0.83	0.67	1.02	0.08	0.00
Eubacteriumruminantiumgroup.id.11340	genus	T2D	0.10	0.12	-0.51	0.34	1.03	0.83	1.29	0.77	0.66
Eubacteriumventriosumgroup.id.11341	genus	T2D	-0.03	0.11	-0.14	0.36	0.96	0.78	1.19	0.74	0.00
Eubacteriumxylanophilumgroup.id.14375	genus	T2D	-0.05	0.11	0.22	0.25	0.99	0.81	1.22	0.95	0.00
Ruminococcusgauvreauiigroup.id.11342	genus	T2D	-0.07	0.11	-0.25	0.29	0.91	0.75	1.11	0.35	0.00
Ruminococcusgnavusgroup.id.14376	genus	T2D	0.07	0.11	-0.72	0.91	1.06	0.86	1.32	0.59	0.00
Ruminococcustorquesgroup.id.14377	genus	T2D	0.05	0.11	0.21	0.15	1.11	0.94	1.32	0.21	0.00
Acidaminococcus.id.2167	genus	T2D	0.16	0.09	-3.22	8.23	1.17	0.98	1.40	0.08	0.00
Adlercreutzia.id.812	genus	T2D	0.03	0.11	-0.33	0.37	1.00	0.81	1.22	0.97	0.00
Akkermansia.id.4037	genus	T2D	-0.10	0.11	0.24	0.14	1.04	0.87	1.23	0.70	0.71
Alistipes.id.968	genus	T2D	-0.14	0.11	-0.02	0.31	0.88	0.72	1.08	0.23	0.00
Alloprevotella.id.961	genus	T2D	-0.11	0.10	-0.05	0.27	0.90	0.74	1.09	0.27	0.00
Anaerostipes.id.1991	genus	T2D	-0.17	0.11	-0.92	0.47	0.82	0.67	1.00	0.05	0.59
Anaerotruncus.id.2054	genus	T2D	-0.16	0.11	-0.10	0.27	0.86	0.70	1.05	0.14	0.00
Bacteroides.id.918	genus	T2D	-0.13	0.10	0.25	0.18	0.96	0.81	1.15	0.67	0.70
Barnesiella.id.944	genus	T2D	-0.08	0.12	0.17	0.15	1.02	0.85	1.23	0.82	0.42
Bifidobacterium.id.436	genus	T2D	0.12	0.11	-0.06	0.26	1.10	0.90	1.33	0.36	0.00
Blautia.id.1992	genus	T2D	-0.69	0.13	-0.11	0.22	0.58	0.47	0.72	0.00	0.81
Butyricicoccus.id.2055	genus	T2D	-0.13	0.11	0.18	0.22	0.93	0.77	1.13	0.47	0.35
Butyricimonas.id.945	genus	T2D	0.04	0.11	0.14	0.19	1.07	0.88	1.29	0.50	0.00
Butyrivibrio.id.1993	genus	T2D	-0.28	0.11	-0.32	0.37	0.75	0.61	0.93	0.01	0.00
Catenibacterium.id.2153	genus	T2D	-0.10	0.11	0.26	0.18	1.00	0.83	1.20	1.00	0.64
ChristensenellaceaeR7group.id.11283	genus	T2D	-0.35	0.10	0.27	0.23	0.79	0.65	0.95	0.01	0.83

© 2021 Chen Z et al. *JAMA Network Open.*

Clostridiumsensustricto1.id.1873	genus	T2D	-0.31	0.11	0.03	0.22	0.78	0.65	0.95	0.01	0.47
Collinsella.id.815	genus	T2D	0.03	0.11	0.04	0.19	1.03	0.86	1.25	0.72	0.00

© 2021 Chen Z et al. *JAMA Network Open.*

Coprococcus1.id.11301	genus	T2D	-0.05	0.11	-0.67	0.51	0.92	0.75	1.15	0.47	0.31
Coprococcus2.id.11302	genus	T2D	0.10	0.11	-0.29	0.41	1.07	0.87	1.32	0.51	0.00
Coprococcus3.id.11303	genus	T2D	0.25	0.10	-0.02	0.15	1.18	1.01	1.39	0.04	0.53
Desulfovibrio.id.3173	genus	T2D	-0.11	0.11	-0.56	0.42	0.87	0.71	1.07	0.19	0.09
Dialister.id.2183	genus	T2D	-0.03	0.11	-716.05	68684.93	0.97	0.78	1.21	0.76	0.00
Dorea.id.1997	genus	T2D	-0.11	0.12	-0.60	0.89	0.89	0.70	1.13	0.34	0.00
Eggerthella.id.819	genus	T2D	-0.01	0.11	-0.35	0.58	0.98	0.80	1.20	0.84	0.00
Eisenbergiella.id.11304	genus	T2D	0.06	0.11	-0.11	0.31	1.04	0.84	1.28	0.71	0.00
Enterococcus.id.1831	genus	T2D	0.12	0.11	-8.32	6.35	1.13	0.92	1.39	0.26	0.43
Enterorhabdus.id.820	genus	T2D	-0.14	0.12	0.04	0.27	0.89	0.72	1.10	0.28	0.00
Erysipelatoclostridium.id.11381	genus	T2D	-0.13	0.11	-0.44	0.41	0.86	0.69	1.06	0.16	0.00
ErysipelotrichaceaeUCG003.id.11384	genus	T2D	-0.06	0.11	-0.13	0.35	0.94	0.76	1.15	0.55	0.00
Faecalibacterium.id.2057	genus	T2D	-0.04	0.12	0.13	0.21	1.00	0.82	1.22	1.00	0.00
FamilyXIIIAD3011group.id.11293	genus	T2D	0.00	0.11	-0.66	0.59	0.97	0.79	1.21	0.81	0.17
FamilyXIIIUCG001.id.11294	genus	T2D	-0.12	0.11	-0.91	0.62	0.86	0.70	1.07	0.18	0.37
Fusicatenibacter.id.11305	genus	T2D	0.07	0.11	-371.34	31676.30	1.08	0.87	1.33	0.50	0.00
Haemophilus.id.3698	genus	T2D	-0.03	0.11	0.00	0.17	0.98	0.81	1.17	0.80	0.00
Holdemanella.id.11393	genus	T2D	0.05	0.11	-0.24	0.47	1.03	0.84	1.27	0.76	0.00
Intestinibacter.id.11345	genus	T2D	-0.01	0.11	-0.41	0.88	0.98	0.79	1.22	0.88	0.00
Intestinimonas.id.2062	genus	T2D	-0.01	0.11	-1.33	3.97	0.99	0.79	1.24	0.90	0.00
Lachnoclostridium.id.11308	genus	T2D	-0.06	0.11	0.10	0.20	0.98	0.80	1.18	0.80	0.00
Lachnospira.id.2004	genus	T2D	-0.67	0.11	-1.08	0.61	0.51	0.41	0.63	0.00	0.00
LachnospiraceaeFCS020group.id.11314	genus	T2D	-0.03	0.11	-0.35	0.33	0.94	0.77	1.15	0.55	0.00
LachnospiraceaeND3007group.id.11317	genus	T2D	-0.36	0.11	0.23	0.17	0.84	0.70	1.00	0.05	0.88
LachnospiraceaeNK4A136group.id.11319	genus	T2D	-0.36	0.11	0.01	0.27	0.74	0.60	0.91	0.00	0.36
LachnospiraceaeUCG001.id.11321	genus	T2D	-0.16	0.11	-0.01	0.28	0.87	0.72	1.07	0.19	0.00
LachnospiraceaeUCG004.id.11324	genus	T2D	-0.31	0.11	-0.38	0.41	0.73	0.60	0.90	0.00	0.00
LachnospiraceaeUCG010.id.11330	genus	T2D	-0.19	0.10	-0.23	0.33	0.83	0.68	1.01	0.06	0.00
Lactobacillus.id.1837	genus	T2D	-0.12	0.11	-0.12	0.32	0.89	0.72	1.10	0.27	0.00
Marvinbryantia.id.2005	genus	T2D	0.02	0.11	0.01	0.25	1.02	0.84	1.24	0.84	0.00
Megamonas.id.2184	genus	T2D	-0.30	0.11	-0.20	0.31	0.75	0.62	0.91	0.00	0.00
Megasphaera.id.2185	genus	T2D	-0.14	0.12	0.02	0.21	0.90	0.74	1.10	0.33	0.00

© 2021 Chen Z et al. *JAMA Network Open.*

Mitsuokella.id.2186	genus	T2D	-0.33	0.12	-0.03	0.28	0.75	0.61	0.93	0.01	0.00
Odoribacter.id.952	genus	T2D	-0.14	0.11	-0.31	0.31	0.86	0.70	1.05	0.13	0.00

© 2021 Chen Z et al. *JAMA Network Open.*

Olsenella.id.822		genus	T2D	-0.16	0.11	0.05	0.24	0.89	0.73	1.07	0.21	0.00
Oscillospira.id.2064		genus	T2D	-0.18	0.11	0.07	0.24	0.87	0.72	1.06	0.17	0.00
Parabacteroides.id.954		genus	T2D	-0.11	0.11	0.15	0.24	0.93	0.77	1.13	0.46	0.00
Paraprevotella.id.962		genus	T2D	-0.16	0.13	0.05	0.17	0.92	0.76	1.13	0.43	0.02
Parasutterella.id.2892		genus	T2D	-0.13	0.12	0.22	0.19	0.96	0.79	1.17	0.70	0.60
Peptococcus.id.2037		genus	T2D	-0.17	0.12	-0.34	0.38	0.83	0.67	1.03	0.10	0.00
Phascolarctobacterium.id.2168		genus	T2D	0.34	0.11	0.41	0.16	1.43	1.20	1.71	0.00	0.00
Prevotella2.id.11180		genus	T2D	0.04	0.11	0.34	0.21	1.11	0.91	1.35	0.29	0.33
Prevotella7.id.11182		genus	T2D	-0.01	0.11	-295.81	23456.74	0.99	0.79	1.22	0.90	0.00
Prevotella9.id.11183		genus	T2D	-0.03	0.12	-0.69	0.67	0.95	0.76	1.19	0.65	0.00
PrevotellaceaeNK3B31group.id.11185		genus	T2D	-0.42	0.13	0.15	0.12	0.89	0.74	1.06	0.19	0.90
PrevotellaceaeUCG001.id.11186		genus	T2D	-0.25	0.12	0.05	0.19	0.84	0.69	1.03	0.09	0.44
RikenellaceaeRC9gutgroup.id.11191		genus	T2D	0.14	0.10	-133.93	10258.92	1.15	0.94	1.40	0.17	0.00
Romboutsia.id.11347		genus	T2D	0.01	0.11	0.13	0.15	1.05	0.89	1.25	0.56	0.00
Roseburia.id.2012		genus	T2D	0.03	0.10	-399.25	40424.84	1.04	0.84	1.27	0.74	0.00
Ruminiclostridium5.id.11355		genus	T2D	-0.08	0.11	-0.57	0.35	0.88	0.72	1.09	0.25	0.44
Ruminiclostridium6.id.11356		genus	T2D	0.07	0.12	0.25	0.23	1.11	0.90	1.36	0.32	0.00
Ruminiclostridium9.id.11357		genus	T2D	-0.02	0.12	-0.07	0.27	0.97	0.79	1.19	0.78	0.00
RuminococcaceaeNK4A214group.id.11358		genus	T2D	-0.19	0.10	-0.15	0.22	0.84	0.70	1.00	0.05	0.00
RuminococcaceaeUCG002.id.11360		genus	T2D	-0.07	0.11	0.14	0.24	0.97	0.79	1.19	0.77	0.00
RuminococcaceaeUCG003.id.11361		genus	T2D	0.04	0.11	-0.04	0.31	1.03	0.84	1.27	0.75	0.00
RuminococcaceaeUCG004.id.11362		genus	T2D	0.09	0.11	0.12	0.14	1.11	0.93	1.32	0.24	0.00
RuminococcaceaeUCG005.id.11363		genus	T2D	0.00	0.11	-0.18	0.28	0.98	0.80	1.20	0.83	0.00
RuminococcaceaeUCG008.id.11365		genus	T2D	-0.11	0.12	-0.01	0.25	0.92	0.74	1.13	0.41	0.00
RuminococcaceaeUCG010.id.11367		genus	T2D	0.35	0.11	0.36	0.17	1.43	1.19	1.71	0.00	0.00
RuminococcaceaeUCG013.id.11370		genus	T2D	-0.19	0.12	-0.29	0.39	0.82	0.66	1.02	0.08	0.00
RuminococcaceaeUCG014.id.11371		genus	T2D	-0.09	0.12	0.18	0.16	1.01	0.84	1.22	0.89	0.45
Ruminococcus1.id.11373		genus	T2D	-0.05	0.11	-0.03	0.28	0.95	0.78	1.17	0.65	0.00
Ruminococcus2.id.11374		genus	T2D	-0.13	0.11	0.09	0.28	0.91	0.74	1.11	0.33	0.00
Sellimonas.id.14369		genus	T2D	0.00	0.11	0.01	0.23	1.00	0.82	1.22	0.98	0.00
Senegalimassilia.id.11160		genus	T2D	0.04	0.11	0.29	0.24	1.09	0.89	1.32	0.41	0.00
Slackia.id.825		genus	T2D	-0.08	0.11	-0.57	0.35	0.88	0.72	1.09	0.25	0.43

© 2021 Chen Z et al. *JAMA Network Open.*

Streptococcus.id.1853	genus	T2D	-0.08	0.12	-0.05	0.27	0.93	0.75	1.14	0.47	0.00
Subdoligranulum.id.2070	genus	T2D	-0.06	0.12	-0.03	0.23	0.95	0.78	1.16	0.62	0.00

© 2021 Chen Z et al. *JAMA Network Open.*

Succiniclasticum.id.2169	genus	T2D	0.05	0.11	0.11	0.16	1.07	0.89	1.28	0.46	0.00
Sutterella.id.2896	genus	T2D	-0.23	0.12	-0.27	0.38	0.79	0.64	0.99	0.04	0.00
Terrisporobacter.id.11348	genus	T2D	-0.01	0.11	0.01	0.23	1.00	0.82	1.22	0.98	0.00
Turicibacter.id.2162	genus	T2D	-0.11	0.12	-0.01	0.25	0.92	0.74	1.13	0.41	0.00
Tyzzerella3.id.11335	genus	T2D	-0.19	0.12	-0.29	0.39	0.82	0.66	1.02	0.08	0.00
Veillonella.id.2198	genus	T2D	-0.07	0.12	-0.67	0.96	0.92	0.73	1.16	0.49	0.00
unknowngenus.id.1000000073	genus	T2D	-0.19	0.13	0.03	0.23	0.87	0.70	1.08	0.22	0.00
unknowngenus.id.1000001215	genus	T2D	-0.11	0.12	-0.01	0.25	0.92	0.74	1.13	0.41	0.00
unknowngenus.id.1000005472	genus	T2D	-0.19	0.12	-0.29	0.39	0.82	0.66	1.02	0.08	0.00
unknowngenus.id.1000005479	genus	T2D	-0.05	0.11	-1.02	0.85	0.93	0.75	1.16	0.53	0.21
unknowngenus.id.1000013899	genus	T2D	-0.07	0.12	-0.67	0.96	0.92	0.73	1.16	0.49	0.00
unknowngenus.id.2071	genus	T2D	-0.33	0.12	0.11	0.19	0.81	0.66	0.99	0.04	0.73
unknowngenus.id.2755	genus	T2D	-0.05	0.11	-0.05	0.31	0.95	0.77	1.17	0.62	0.00
unknowngenus.id.826	genus	T2D	-0.06	0.11	-0.16	0.30	0.93	0.76	1.14	0.50	0.00
unknowngenus.id.964	genus	T2D	-0.06	0.12	-228.80	24024.42	0.95	0.74	1.20	0.65	0.00

**Taxa in RS only**	**Clade**	**Phenotype**	**Beta_RS**	**Se_RS**
family.BacteroidalesS247group.id.11173	family	T2D	-0.03	0.06
family.Veillonellaceae.id.2172	family	T2D	-0.01	0.05
genus..Bacteroidespectinophilusgroup.id.143	family	T2D	-0.99	0.69
genus.Enterobacter.id.3502	genus	T2D	0.22	0.15
genus.EscherichiaShigella.id.3504	genus	T2D	0.01	0.04
genus..Eubacteriumcoprostanoligenesgroup.i	genus	T2D	-0.25	0.08
genus..Eubacteriumeligensgroup.id.14372	genus	T2D	-0.18	0.06
genus..Eubacteriumhalliigroup.id.11338	genus	T2D	0.08	0.12
genus..Eubacteriumoxidoreducensgroup.id.1	genus	T2D	-0.18	0.11
genus..Eubacteriumxylanophilumgroup.id.14	genus	T2D	-0.27	0.08
genus.Hungatella.id.11306	genus	T2D	-0.03	0.14
genus.Klebsiella.id.3507	genus	T2D	0.13	0.09
genus.LachnospiraceaeNC2004group.id.1131	genus	T2D	-0.34	0.11

© 2021 Chen Z et al. *JAMA Network Open.*

genus.LachnospiraceaeUCG008.id.11328	genus	T2D	-0.09	0.13
genus.unknowngenus.id.1868	genus	T2D	-0.27	0.12

© 2021 Chen Z et al. *JAMA Network Open.*

Taxa in LLD only	Clade	Phenotype	Beta_LLD	Se_LLD
domain.Archaea.id.2	Clade	T2D	0.07	0.22
phylum.Euryarchaeota.id.55	domain	T2D	0.07	0.22
phylum.Lentisphaerae.id.2238	phylum	T2D	-0.04	0.22
order.Actinomycetales.id.420	phylum	T2D	0.12	0.15
order.Mycoplasmatales.id.3946	order	T2D	-0.40	53.21
order.NB1.n.id.3953	order	T2D	0.07	0.17
order.Pseudomonadales.id.3709	order	T2D	-0.16	2.54
order.Spirochaetales.id.3870	order	T2D	-17.48	2064.40
order.Synergistales.id.3900	order	T2D	-1.18	1.73
order.Thermoplasmatales.id.220	order	T2D	0.20	0.10
order.unknownorder.id.1000000003	order	T2D	-0.27	0.66
order.Verrucomicrobiales.id.4030	order	T2D	0.01	0.23
order.Victivallales.id.2254	order	T2D	-0.04	0.22
order.Xanthomonadales.id.3786	order	T2D	-21.14	2353.29
class.Spirochaetes.id.3856	order	T2D	-17.48	2064.40
class.Synergistia.id.3899	class	T2D	-1.18	1.73
class.Thermoplasmata.id.210	class	T2D	0.20	0.10
family.Actinomycetaceae.id.421	class	T2D	0.12	0.15
family.BacteroidalesS24.7group.id.11173	family	T2D	-1.02	0.85
family.Christensenellaceae.id.1866	family	T2D	-0.33	0.34
family.FamilyXI.id.1936	family	T2D	-0.54	0.82
family.unknownfamily.id.1000000004	family	T2D	-0.27	0.66
family.unknownfamily.id.1000006161	family	T2D	0.07	0.17
family.unknownfamily.id.1855	family	T2D	0.14	0.08
family.vadinBE97.id.14446	family	T2D	-0.53	1.53
family.Veillonellaceae.id.2172	family	T2D	0.18	0.26
family.Xanthomonadaceae.id.3799	family	T2D	-21.14	2353.29
genus..Clostridiuminnocuumgroup.id.14397	family	T2D	0.14	0.12
genus..Eubacteriumbrachygroup.id.11296	family	T2D	0.36	0.13
genus.Abiotrophia.id.1803	genus	T2D	-12.81	1010.99
genus.Actinomyces.id.423	genus	T2D	0.08	0.15

© 2021 Chen Z et al. *JAMA Network Open.*

genus.Actinotignum.id.11137	genus	T2D	-13.70	1171.94
genus.Allisonella.id.2174	genus	T2D	-0.23	0.36

© 2021 Chen Z et al. *JAMA Network Open.*

genus.Arcanobacterium.id.424	genus	T2D	-17.67	1662.07
genus.Asaccharobacter.id.813	genus	T2D	0.15	0.22
genus.Bilophila.id.3170	genus	T2D	0.14	0.24
genus.CandidatusMethanogranum.id.11110	genus	T2D	-4.30	423.74
genus.Catenisphaera.id.14395	genus	T2D	-796.47	70906.35
genus.Cellulosilyticum.id.1995	genus	T2D	-81.38	6776.06
genus.Cetobacterium.id.2209	genus	T2D	-0.40	53.21
genus.Cloacibacillus.id.3908	genus	T2D	-0.57	0.94
genus.Coprobacillus.id.2154	genus	T2D	-0.74	1.62
genus.CoriobacteriaceaeUCG.002.id.11158	genus	T2D	-0.36	0.59
genus.CoriobacteriaceaeUCG.003.id.11159	genus	T2D	-286.12	29432.75
genus.Denitrobacterium.id.818	genus	T2D	0.19	0.13
genus.dgA.11gutgroup.id.978	genus	T2D	-0.46	53.21
genus.Dielma.id.11380	genus	T2D	-0.21	1.79
genus.Epulopiscium.id.1998	genus	T2D	-2.27	3.23
genus.ErysipelotrichaceaeUCG006.id.11386	genus	T2D	-6.92	589.73
genus.ErysipelotrichaceaeUCG009.id.11389	genus	T2D	-0.49	53.21
genus.Escherichia.Shigella.id.3504	genus	T2D	0.10	0.14
genus.Eubacterium.id.1932	genus	T2D	0.26	0.19
genus.Faecalicoccus.id.11391	genus	T2D	-70.01	4858.27
genus.Faecalitalea.id.11392	genus	T2D	0.16	0.10
genus.Flavonifractor.id.2059	genus	T2D	-0.13	0.47
genus.Gardnerella.id.437	genus	T2D	-15.75	1536.96
genus.Gordonibacter.id.821	genus	T2D	-0.29	0.44
genus.Hafnia.Obesumbacterium.id.14636	genus	T2D	0.31	0.48
genus.Howardella.id.2000	genus	T2D	-0.48	0.41
genus.LachnospiraceaeNK3A20group.id.1131	genus	T2D	-2.54	322.11
genus.Lactococcus.id.1851	genus	T2D	-0.36	0.67
genus.Lautropia.id.2905	genus	T2D	-38.98	5070.09
genus.Leuconostoc.id.1841	genus	T2D	-0.71	0.77
genus.Methanobrevibacter.id.123	genus	T2D	0.10	0.21
genus.Methanosphaera.id.124	genus	T2D	-0.37	0.52

© 2021 Chen Z et al. *JAMA Network Open.*

genus.Mogibacterium.id.1960	genus	T2D	-1.64	1.50
genus.Morganella.id.3512	genus	T2D	-0.33	53.21

© 2021 Chen Z et al. *JAMA Network Open.*

genus.Porphyromonas.id.956	genus	T2D	-1.66	1.96
genus.Prevotella1.id.11179	genus	T2D	-1.06	156.90
genus.PrevotellaceaeUCG003.id.11187	genus	T2D	-58.76	7137.04
genus.PrevotellaceaeUCG004.id.11188	genus	T2D	-6.17	507.98
genus.Pseudomonas.id.3723	genus	T2D	-1030.30	83303.57
genus.Rikenella.id.973	genus	T2D	-15.35	1241.32
genus.RuminococcaceaeNK4A214group.id.11	genus	T2D	-0.38	0.41
genus.RuminococcaceaeUCG005.id.11363	genus	T2D	-0.20	0.31
genus.RuminococcaceaeUCG008.id.11365	genus	T2D	0.02	0.21
genus.RuminococcaceaeUCG010.id.11367	genus	T2D	-0.03	0.28
genus.Sarcina.id.1896	genus	T2D	-55.99	5591.92
genus.Solobacterium.id.2161	genus	T2D	-2.95	4.22
genus.Staphylococcus.id.1780	genus	T2D	0.00	0.25
genus.Stenotrophomonas.id.3818	genus	T2D	-0.42	53.21
genus.Succinivibrio.id.3331	genus	T2D	-260.73	22878.78
genus.Synergistes.id.3913	genus	T2D	-25.46	2062.61
genus.Syntrophococcus.id.2016	genus	T2D	-0.87	89.99
genus.Treponema2.id.11577	genus	T2D	-15.52	1932.94
genus.Tyzzerella4.id.11336	genus	T2D	-2.95	7.15
genus.unknowngenus.id.1000000005	genus	T2D	-0.27	0.66
genus.unknowngenus.id.1000027565	genus	T2D	-0.53	1.53
genus.unknowngenus.id.1000043796	genus	T2D	0.14	0.08
genus.unknowngenus.id.1854	genus	T2D	-41.03	3041.97
genus.unknowngenus.id.2163	genus	T2D	0.15	0.11
genus.unknowngenus.id.3332	genus	T2D	-35.59	4287.44
genus.unknowngenus.id.441	genus	T2D	-0.18	0.38
genus.Ureaplasma.id.3951	genus	T2D	-0.40	53.21
genus.Varibaculum.id.427	genus	T2D	0.28	0.14
genus.Veillonella.id.2198	genus	T2D	0.13	0.15
genus.Weissella.id.1843	genus	T2D	0.09	0.14

© 2021 Chen Z et al. *JAMA Network Open.*

Supplementary Table 3 - Associations of taxa and type 2 diabetes

Effect estimates in RS and LLD were calculated using logistic regression; pooled estimates were calculated based on a mixed-effect meta-analysis.

Model 3: Adjusted for age, sex, Time in mail (RS), Batch (RS), smoking, education level (RS), physical activity, alcohol intake, total energy intake, and BMI.
Abbreviation RS, Rotterdam Study, LLD, Lifelines-Deep Study. P<0.0005 indicates statistical significance

Overlapping taxa in RS and LLD	Clade	Phenotype	Beta_RS	Se_RS	Beta_LLD	Se_LLD	OR_Meta	Lower95%CI_Meta	Upper95%CI_Meta	P_Meta	I^2
Bacteria.id.3	domain	T2D	-0.09	0.11	0.22	0.50	1.02	0.97	1.08	0.37	0.00
Actinobacteria.id.400	phylum	T2D	0.02	0.11	0.31	0.24	1.08	0.88	1.32	0.47	0.12
Bacteroidetes.id.905	phylum	T2D	-0.05	0.11	-0.49	0.34	0.91	0.74	1.13	0.39	0.36
Cyanobacteria.id.1500	phylum	T2D	-0.02	0.12	-0.02	0.27	0.98	0.79	1.22	0.87	0.00
Firmicutes.id.1672	phylum	T2D	-0.08	0.12	-0.11	0.24	0.92	0.75	1.13	0.43	0.00
Proteobacteria.id.2375	phylum	T2D	0.10	0.12	0.12	0.15	1.12	0.93	1.34	0.23	0.00
Tenericutes.id.3919	phylum	T2D	-0.09	0.12	-0.19	0.36	0.90	0.72	1.13	0.36	0.00
Verrucomicrobia.id.3982	phylum	T2D	0.06	0.12	0.09	0.23	1.07	0.87	1.30	0.53	0.00
Actinobacteria.id.419	class	T2D	0.08	0.12	0.31	0.23	1.14	0.92	1.40	0.23	0.00
Alphaproteobacteria.id.2379	class	T2D	0.02	0.12	-0.01	0.32	1.01	0.81	1.26	0.91	0.00
Bacilli.id.1673	class	T2D	0.26	0.12	0.31	0.18	1.32	1.09	1.59	0.00	0.00
Bacteroidia.id.912	class	T2D	-0.05	0.11	-0.50	0.34	0.91	0.74	1.13	0.39	0.37
Betaproteobacteria.id.2867	class	T2D	0.06	0.12	-0.02	0.26	1.05	0.85	1.30	0.64	0.00
Clostridia.id.1859	class	T2D	-0.17	0.11	-0.20	0.23	0.84	0.70	1.02	0.07	0.00
Coriobacteriia.id.809	class	T2D	-0.06	0.12	0.11	0.25	0.97	0.79	1.19	0.78	0.00
Deltaproteobacteria.id.3087	class	T2D	0.10	0.12	0.00	0.30	1.10	0.89	1.36	0.40	0.00
Erysipelotrichia.id.2147	class	T2D	-0.02	0.12	-0.24	0.30	0.95	0.77	1.18	0.66	0.00
Gammaproteobacteria.id.3303	class	T2D	0.11	0.12	0.12	0.14	1.12	0.94	1.34	0.21	0.00
Melainabacteria.id.1589	class	T2D	-0.05	0.12	0.02	0.25	0.96	0.78	1.19	0.73	0.00
Mollicutes.id.3920	class	T2D	-0.09	0.12	-0.19	0.36	0.90	0.72	1.13	0.36	0.00
Negativicutes.id.2164	class	T2D	-0.11	0.11	0.14	0.27	0.93	0.76	1.14	0.48	0.00
Verrucomicrobiae.id.4029	class	T2D	0.06	0.12	0.09	0.23	1.07	0.87	1.31	0.51	0.00
Aeromonadales.id.3316	order	T2D	0.07	0.11	-413.18	40214.87	1.07	0.87	1.32	0.52	0.00
Bacteroidales.id.913	order	T2D	-0.05	0.11	-0.50	0.34	0.91	0.74	1.13	0.39	0.37
Bifidobacteriales.id.432	order	T2D	0.04	0.12	0.30	0.24	1.09	0.89	1.35	0.40	0.00
Burkholderiales.id.2874	order	T2D	0.06	0.12	-0.02	0.26	1.05	0.85	1.30	0.66	0.00
Clostridiales.id.1863	order	T2D	-0.17	0.11	-0.20	0.23	0.84	0.70	1.02	0.07	0.00
Coriobacteriales.id.810	order	T2D	-0.06	0.12	0.11	0.25	0.97	0.79	1.19	0.78	0.00
Desulfovibrionales.id.3156	order	T2D	0.10	0.12	0.01	0.30	1.10	0.89	1.36	0.40	0.00

© 2021 Chen Z et al. *JAMA Network Open.*

Enterobacteriales.id.3468	order	T2D	0.12	0.12	0.14	0.14	1.14	0.95	1.36	0.16	0.00
Erysipelotrichales.id.2148	order	T2D	-0.02	0.12	-0.24	0.30	0.95	0.77	1.18	0.66	0.00
Gastranaerophilales.id.1591	order	T2D	-0.05	0.12	0.02	0.25	0.96	0.78	1.20	0.73	0.00
Lactobacillales.id.1800	order	T2D	0.25	0.12	0.32	0.18	1.31	1.08	1.58	0.01	0.00
MollicutesRF9.id.11579	order	T2D	-0.07	0.12	-0.20	0.37	0.92	0.74	1.15	0.48	0.00
Pasteurellales.id.3688	order	T2D	-0.07	0.12	0.19	0.16	1.03	0.85	1.24	0.79	0.45
Rhodospirillales.id.2667	order	T2D	0.03	0.12	0.01	0.29	1.03	0.83	1.27	0.82	0.00
Selenomonadales.id.2165	order	T2D	-0.11	0.11	0.14	0.27	0.93	0.76	1.14	0.48	0.00
Verrucomicrobiales.id.4030	order	T2D	0.06	0.12	0.09	0.23	1.07	0.87	1.31	0.51	0.00
Acidaminococcaceae.id.2166	family	T2D	-0.11	0.11	-1.26	0.72	0.87	0.70	1.09	0.23	0.60
Alcaligenaceae.id.2875	family	T2D	0.05	0.12	-0.04	0.27	1.04	0.84	1.28	0.72	0.00
Bacteroidaceae.id.917	family	T2D	0.00	0.12	-0.36	0.33	0.96	0.77	1.19	0.70	0.03
Bifidobacteriaceae.id.433	family	T2D	0.04	0.12	0.30	0.24	1.09	0.89	1.35	0.40	0.00
Christensenellaceae.id.1866	family	T2D	-0.25	0.11	-0.24	0.35	0.78	0.64	0.96	0.02	0.00
Clostridiaceae1.id.1869	family	T2D	-0.83	0.13	-0.11	0.25	0.51	0.40	0.64	0.00	0.84
ClostridialesvadinBB60group.id.11286	family	T2D	-0.08	0.13	0.08	0.22	0.96	0.77	1.19	0.71	0.00
Coriobacteriaceae.id.811	family	T2D	-0.06	0.12	0.11	0.25	0.97	0.79	1.19	0.78	0.00
Desulfovibrionaceae.id.3169	family	T2D	0.10	0.12	0.00	0.30	1.10	0.89	1.36	0.40	0.00
Enterobacteriaceae.id.3469	family	T2D	0.12	0.12	0.14	0.14	1.14	0.95	1.36	0.16	0.00
Enterococcaceae.id.1828	family	T2D	0.12	0.09	-0.44	1.11	1.13	0.94	1.36	0.21	0.00
Erysipelotrichaceae.id.2149	family	T2D	-0.02	0.12	-0.24	0.30	0.95	0.77	1.18	0.66	0.00
FamilyXIII.id.1957	family	T2D	-0.16	0.11	-0.41	0.34	0.84	0.68	1.02	0.08	0.00
Lachnospiraceae.id.1987	family	T2D	0.09	0.12	-0.16	0.26	1.05	0.84	1.30	0.68	0.00
Lactobacillaceae.id.1836	family	T2D	0.20	0.10	-0.06	0.14	1.11	0.94	1.30	0.21	0.59
Pasteurellaceae.id.3689	family	T2D	-0.07	0.12	0.19	0.16	1.03	0.85	1.24	0.79	0.45
Peptococcaceae.id.2024	family	T2D	-0.08	0.12	-0.55	0.55	0.90	0.72	1.13	0.37	0.00
Peptostreptococcaceae.id.2042	family	T2D	-0.67	0.11	-0.33	0.30	0.53	0.43	0.66	0.00	0.13
Porphyromonadaceae.id.943	family	T2D	-0.09	0.12	-0.44	0.36	0.88	0.71	1.10	0.28	0.00
Prevotellaceae.id.960	family	T2D	0.04	0.12	-0.40	0.48	1.02	0.82	1.27	0.86	0.00
Rhodospirillaceae.id.2717	family	T2D	0.03	0.12	0.01	0.29	1.03	0.83	1.27	0.82	0.00
Rikenellaceae.id.967	family	T2D	-0.10	0.11	0.10	0.25	0.93	0.76	1.14	0.50	0.00
Ruminococcaceae.id.2050	family	T2D	-0.18	0.11	0.20	0.28	0.88	0.72	1.07	0.19	0.37

© 2021 Chen Z et al. *JAMA Network Open.*

Streptococcaceae.id.1850	family	T2D	0.23	0.11	0.39	0.17	1.32	1.10	1.59	0.00	0.00
Succinivibrionaceae.id.3326	family	T2D	0.07	0.11	-413.19	40223.44	1.07	0.87	1.32	0.52	0.00

© 2021 Chen Z et al. *JAMA Network Open.*

Veillonellaceae.id.2172	family	T2D	-0.08	0.12	0.22	0.25	0.97	0.79	1.19	0.78	0.15
Verrucomicrobiaceae.id.4036	family	T2D	0.06	0.12	0.09	0.23	1.07	0.87	1.31	0.51	0.00
unknownfamily.id.1000001214	family	T2D	-0.05	0.12	0.02	0.25	0.96	0.78	1.20	0.73	0.00
unknownfamily.id.1000005471	family	T2D	-0.07	0.12	-0.20	0.37	0.92	0.74	1.15	0.48	0.00
unknownfamily.id.987	family	T2D	-0.09	0.12	-0.90	1.09	0.91	0.71	1.15	0.43	0.00
Bacteroidespectinophilusgroup.id.14371	genus	T2D	-0.74	0.57	-0.08	0.35	0.77	0.43	1.38	0.38	0.00
Eubacteriumcoprostanoligenesgroup.id.11375	genus	T2D	-0.25	0.10	-0.31	0.31	0.77	0.64	0.93	0.01	0.00
Eubacteriumeligensgroup.id.14372	genus	T2D	-0.27	0.12	0.20	0.18	0.88	0.73	1.07	0.20	0.79
Eubacteriumhalliigroup.id.11338	genus	T2D	0.03	0.12	-0.21	0.29	0.99	0.80	1.23	0.95	0.00
Eubacteriumrectalegroup.id.14374	genus	T2D	0.02	0.12	0.09	0.16	1.05	0.87	1.27	0.62	0.00
Eubacteriumruminantiumgroup.id.11340	genus	T2D	-0.15	0.12	0.17	0.20	0.94	0.77	1.16	0.58	0.46
Eubacteriumventriosumgroup.id.11341	genus	T2D	-0.12	0.12	0.06	0.22	0.92	0.75	1.13	0.43	0.00
Eubacteriumxylanophilumgroup.id.14375	genus	T2D	-0.30	0.12	-0.26	0.34	0.75	0.60	0.93	0.01	0.00
Ruminococcusgauvreauiigroup.id.11342	genus	T2D	-0.19	0.11	-1.29	0.54	0.79	0.64	0.98	0.03	0.75
Ruminococcusgnavusgroup.id.14376	genus	T2D	-0.09	0.12	-0.08	0.39	0.92	0.74	1.14	0.44	0.00
Ruminococcustorquesgroup.id.14377	genus	T2D	0.23	0.12	-0.39	0.42	1.20	0.95	1.51	0.13	0.51
Acidaminococcus.id.2167	genus	T2D	0.09	0.10	-4.15	5.19	1.10	0.91	1.33	0.34	0.00
Adlercreutzia.id.812	genus	T2D	0.03	0.11	-0.08	0.27	1.01	0.83	1.24	0.89	0.00
Akkermansia.id.4037	genus	T2D	0.06	0.12	0.09	0.23	1.07	0.87	1.31	0.51	0.00
Alistipes.id.968	genus	T2D	-0.06	0.12	0.06	0.26	0.96	0.78	1.18	0.72	0.00
Alloprevotella.id.961	genus	T2D	-0.03	0.12	-2.54	4.04	0.97	0.76	1.23	0.79	0.00
Anaerostipes.id.1991	genus	T2D	-0.07	0.11	-0.02	0.24	0.94	0.77	1.15	0.54	0.00
Anaerotruncus.id.2054	genus	T2D	-0.26	0.11	-0.10	0.30	0.79	0.64	0.97	0.02	0.00
Bacteroides.id.918	genus	T2D	0.00	0.12	-0.36	0.33	0.96	0.77	1.19	0.70	0.03
Barnesiella.id.944	genus	T2D	-0.09	0.12	-0.49	0.42	0.88	0.71	1.11	0.28	0.00
Bifidobacterium.id.436	genus	T2D	0.04	0.12	0.29	0.24	1.09	0.89	1.35	0.41	0.00
Blautia.id.1992	genus	T2D	0.21	0.12	-0.21	0.31	1.17	0.93	1.47	0.17	0.37
Butyricicoccus.id.2055	genus	T2D	0.02	0.11	-0.63	0.45	0.98	0.79	1.22	0.88	0.50
Butyricimonas.id.945	genus	T2D	0.13	0.12	-0.20	0.41	1.11	0.89	1.38	0.35	0.00
Butyrivibrio.id.1993	genus	T2D	-0.09	0.13	-0.37	0.60	0.90	0.71	1.15	0.40	0.00
Catenibacterium.id.2153	genus	T2D	0.04	0.11	-1.87	1.52	1.03	0.83	1.27	0.80	0.36
ChristensenellaceaeR7group.id.11283	genus	T2D	-0.24	0.11	-0.24	0.36	0.79	0.64	0.97	0.02	0.00

© 2021 Chen Z et al. *JAMA Network Open.*

Clostridiumsensustricto1.id.1873	genus	T2D	-0.84	0.13	-0.11	0.26	0.50	0.40	0.63	0.00	0.84
Collinsella.id.815	genus	T2D	0.01	0.12	0.21	0.23	1.05	0.86	1.29	0.63	0.00

© 2021 Chen Z et al. *JAMA Network Open.*

Coprococcus1.id.11301		genus	T2D	0.06	0.12	0.14	0.25	1.07	0.87	1.32	0.50	0.00
Coprococcus2.id.11302		genus	T2D	-0.14	0.12	-0.18	0.34	0.86	0.69	1.07	0.18	0.00
Coprococcus3.id.11303		genus	T2D	0.05	0.12	-0.60	0.35	0.98	0.78	1.23	0.86	0.68
Desulfovibrio.id.3173		genus	T2D	0.04	0.12	-0.11	0.35	1.03	0.83	1.27	0.81	0.00
Dialister.id.2183		genus	T2D	-0.07	0.12	0.27	0.24	0.99	0.80	1.22	0.92	0.36
Dorea.id.1997		genus	T2D	-0.15	0.11	-0.29	0.30	0.85	0.69	1.03	0.10	0.00
Eggerthella.id.819		genus	T2D	0.04	0.12	-1.16	1.15	1.03	0.82	1.30	0.78	0.08
Eisenbergiella.id.11304		genus	T2D	0.11	0.11	0.22	0.15	1.16	0.97	1.37	0.10	0.00
Enterococcus.id.1831		genus	T2D	0.11	0.10	-2.68	7.94	1.11	0.92	1.35	0.26	0.00
Enterorhabdus.id.820		genus	T2D	0.07	0.11	-0.29	0.37	1.04	0.84	1.29	0.72	0.00
Erysipelatoclostridium.id.11381		genus	T2D	-0.06	0.12	0.22	0.15	1.05	0.87	1.26	0.61	0.57
ErysipelotrichaceaeUCG003.id.11384		genus	T2D	-0.15	0.11	-0.12	0.32	0.87	0.70	1.07	0.18	0.00
Faecalibacterium.id.2057		genus	T2D	-0.13	0.10	-0.12	0.28	0.88	0.73	1.06	0.19	0.00
FamilyXIIIAD3011group.id.11293		genus	T2D	-0.08	0.11	-0.84	0.47	0.89	0.72	1.10	0.28	0.60
FamilyXIIIUCG001.id.11294		genus	T2D	-0.12	0.12	-0.10	0.28	0.89	0.72	1.09	0.26	0.00
Fusicatenibacter.id.11305		genus	T2D	-0.22	0.11	0.22	0.19	0.89	0.74	1.07	0.22	0.75
Haemophilus.id.3698		genus	T2D	-0.07	0.12	0.18	0.15	1.03	0.85	1.24	0.77	0.40
Holdemanella.id.11393		genus	T2D	0.07	0.11	-0.04	0.26	1.06	0.86	1.30	0.59	0.00
Intestinibacter.id.11345		genus	T2D	-0.67	0.13	-0.10	0.23	0.59	0.47	0.74	0.00	0.80
Intestinimonas.id.2062		genus	T2D	-0.10	0.11	0.15	0.23	0.95	0.78	1.16	0.62	0.00
Lachnoclostridium.id.11308		genus	T2D	-0.02	0.12	0.13	0.20	1.02	0.84	1.24	0.85	0.00
Lachnospira.id.2004		genus	T2D	-0.26	0.11	-0.33	0.38	0.76	0.62	0.94	0.01	0.00
LachnospiraceaeFCS020group.id.11314		genus	T2D	-0.10	0.12	0.23	0.18	0.99	0.82	1.20	0.94	0.57
LachnospiraceaeND3007group.id.11317		genus	T2D	-0.32	0.11	0.26	0.24	0.80	0.66	0.98	0.03	0.80
LachnospiraceaeNK4A136group.id.11319		genus	T2D	-0.31	0.11	-0.05	0.23	0.77	0.63	0.94	0.01	0.00
LachnospiraceaeUCG001.id.11321		genus	T2D	0.04	0.12	0.03	0.19	1.04	0.85	1.26	0.72	0.00
LachnospiraceaeUCG004.id.11324		genus	T2D	-0.06	0.12	-0.60	0.50	0.92	0.73	1.15	0.44	0.10
LachnospiraceaeUCG010.id.11330		genus	T2D	0.12	0.11	-0.32	0.41	1.09	0.88	1.35	0.44	0.04
Lactobacillus.id.1837		genus	T2D	0.18	0.10	-0.09	0.15	1.09	0.93	1.29	0.29	0.53
Marvinbryantia.id.2005		genus	T2D	-0.06	0.11	-0.54	0.42	0.91	0.73	1.13	0.39	0.19
Megamonas.id.2184		genus	T2D	-0.04	0.11	-710.93	69209.72	0.96	0.77	1.20	0.73	0.00
Megasphaera.id.2185		genus	T2D	-0.13	0.13	-0.72	0.86	0.87	0.68	1.12	0.28	0.00

© 2021 Chen Z et al. *JAMA Network Open.*

Mitsuokella.id.2186	genus	T2D	-0.07	0.12	-0.31	0.58	0.92	0.74	1.16	0.50	0.00
Odoribacter.id.952	genus	T2D	0.11	0.12	-0.06	0.31	1.09	0.88	1.36	0.43	0.00

© 2021 Chen Z et al. *JAMA Network Open.*

Olsenella.id.822	genus	T2D	0.08	0.11	-8.31	6.43	1.08	0.87	1.35	0.48	0.41
Oscillospira.id.2064	genus	T2D	-0.10	0.12	0.09	0.26	0.93	0.75	1.15	0.52	0.00
Parabacteroides.id.954	genus	T2D	-0.08	0.12	-0.36	0.36	0.90	0.72	1.13	0.36	0.00
Paraprevotella.id.962	genus	T2D	-0.04	0.11	-0.10	0.35	0.95	0.77	1.18	0.66	0.00
Parasutterella.id.2892	genus	T2D	-0.02	0.12	0.15	0.20	1.02	0.84	1.25	0.81	0.00
Peptococcus.id.2037	genus	T2D	-0.02	0.12	-0.64	0.60	0.96	0.77	1.20	0.72	0.03
Phascolarctobacterium.id.2168	genus	T2D	-0.10	0.11	-1.01	0.65	0.88	0.71	1.10	0.26	0.49
Prevotella2.id.11180	genus	T2D	0.08	0.11	-396.57	30961.40	1.08	0.87	1.35	0.47	0.00
Prevotella7.id.11182	genus	T2D	-0.05	0.12	-0.05	0.21	0.95	0.78	1.16	0.61	0.00
Prevotella9.id.11183	genus	T2D	0.02	0.11	-0.28	0.46	1.00	0.81	1.24	1.00	0.00
PrevotellaceaeNK3B31group.id.11185	genus	T2D	-0.02	0.12	-0.37	0.88	0.98	0.78	1.23	0.85	0.00
PrevotellaceaeUCG001.id.11186	genus	T2D	0.00	0.12	-1.01	3.17	1.00	0.80	1.26	0.98	0.00
RikenellaceaeRC9gutgroup.id.11191	genus	T2D	-0.05	0.12	0.11	0.20	0.99	0.82	1.21	0.95	0.00
Romboutsia.id.11347	genus	T2D	-0.63	0.12	-1.06	0.61	0.52	0.42	0.66	0.00	0.00
Roseburia.id.2012	genus	T2D	-0.07	0.11	-0.47	0.36	0.90	0.72	1.11	0.31	0.08
Ruminiclostridium5.id.11355	genus	T2D	-0.32	0.11	0.21	0.17	0.86	0.71	1.03	0.10	0.85
Ruminiclostridium6.id.11356	genus	T2D	-0.22	0.12	-0.01	0.29	0.83	0.67	1.02	0.08	0.00
Ruminiclostridium9.id.11357	genus	T2D	-0.05	0.12	0.09	0.27	0.98	0.79	1.20	0.81	0.00
RuminococcaceaeNK4A214group.id.11358	genus	T2D	-0.22	0.11	-0.28	0.41	0.80	0.64	0.99	0.04	0.00
RuminococcaceaeUCG002.id.11360	genus	T2D	-0.12	0.11	-0.15	0.33	0.89	0.72	1.09	0.25	0.00
RuminococcaceaeUCG003.id.11361	genus	T2D	-0.08	0.12	-0.08	0.32	0.92	0.74	1.15	0.48	0.00
RuminococcaceaeUCG004.id.11362	genus	T2D	0.05	0.12	0.07	0.25	1.05	0.86	1.29	0.63	0.00
RuminococcaceaeUCG005.id.11363	genus	T2D	-0.19	0.11	-0.11	0.29	0.84	0.68	1.03	0.10	0.00
RuminococcaceaeUCG008.id.11365	genus	T2D	-0.05	0.12	0.05	0.21	0.97	0.80	1.19	0.80	0.00
RuminococcaceaeUCG010.id.11367	genus	T2D	-0.20	0.12	0.05	0.27	0.85	0.69	1.06	0.15	0.00
RuminococcaceaeUCG013.id.11370	genus	T2D	-0.17	0.11	-0.39	0.33	0.82	0.67	1.02	0.07	0.00
RuminococcaceaeUCG014.id.11371	genus	T2D	-0.06	0.11	0.09	0.23	0.97	0.79	1.18	0.75	0.00
Ruminococcus1.id.11373	genus	T2D	-0.12	0.12	0.08	0.24	0.92	0.75	1.12	0.41	0.00
Ruminococcus2.id.11374	genus	T2D	-0.07	0.11	0.10	0.25	0.96	0.79	1.17	0.70	0.00
Sellimonas.id.14369	genus	T2D	-0.15	0.13	0.05	0.17	0.92	0.75	1.13	0.44	0.00
Senegalimassilia.id.11160	genus	T2D	-0.10	0.12	0.20	0.20	0.97	0.80	1.19	0.80	0.41
Slackia.id.825	genus	T2D	-0.17	0.12	-0.35	0.38	0.83	0.66	1.04	0.11	0.00

© 2021 Chen Z et al. *JAMA Network Open.*

Streptococcus.id.1853	genus	T2D	0.23	0.11	0.39	0.17	1.32	1.10	1.59	0.00	0.00
Subdoligranulum.id.2070	genus	T2D	0.03	0.12	0.33	0.22	1.10	0.90	1.34	0.36	0.35

© 2021 Chen Z et al. *JAMA Network Open.*

Succiniclasticum.id.2169	genus	T2D	0.01	0.12	-287.75	23111.13	1.01	0.80	1.29	0.92	0.00
Sutterella.id.2896	genus	T2D	0.04	0.12	-0.72	0.69	1.02	0.81	1.28	0.89	0.16
Terrisporobacter.id.11348	genus	T2D	-0.43	0.13	0.15	0.13	0.89	0.74	1.06	0.20	0.90
Turicibacter.id.2162	genus	T2D	-0.23	0.12	0.11	0.20	0.87	0.71	1.07	0.19	0.54
Tyzzerella3.id.11335	genus	T2D	0.09	0.11	-130.98	9906.14	1.09	0.89	1.35	0.40	0.00
Veillonella.id.2198	genus	T2D	-0.01	0.11	0.15	0.15	1.04	0.88	1.24	0.62	0.00
unknowngenus.id.1000000073	genus	T2D	-0.08	0.13	0.08	0.22	0.96	0.77	1.19	0.71	0.00
unknowngenus.id.1000001215	genus	T2D	-0.05	0.12	0.02	0.25	0.96	0.78	1.20	0.73	0.00
unknowngenus.id.1000005472	genus	T2D	-0.07	0.12	-0.20	0.37	0.92	0.74	1.15	0.48	0.00
unknowngenus.id.1000005479	genus	T2D	-0.06	0.12	-1.11	0.87	0.92	0.73	1.16	0.50	0.30
unknowngenus.id.1000013899	genus	T2D	-0.09	0.12	-0.90	1.09	0.91	0.71	1.15	0.43	0.00
unknowngenus.id.2071	genus	T2D	-0.23	0.13	0.17	0.19	0.89	0.73	1.10	0.28	0.68
unknowngenus.id.2755	genus	T2D	0.02	0.12	-0.01	0.32	1.02	0.82	1.27	0.86	0.00
unknowngenus.id.826	genus	T2D	-0.04	0.12	-0.12	0.30	0.95	0.77	1.18	0.64	0.00
unknowngenus.id.964	genus	T2D	-0.01	0.13	-223.38	23574.05	0.99	0.77	1.27	0.95	0.00

**Taxa in RS only**	**Clade**	**Phenotype**	**Beta_RS**	**Se_RS**
family.BacteroidalesS247group.id.11173	family	T2D	-0.06	0.12
family.Veillonellaceae.id.2172	family	T2D	-0.08	0.12
genus..Bacteroidespectinophilusgroup.id.1437	family	T2D	-0.74	0.57
genus.Enterobacter.id.3502	genus	T2D	0.15	0.11
genus.EscherichiaShigella.id.3504	genus	T2D	0.03	0.12
genus..Eubacteriumcoprostanoligenesgroup.id	genus	T2D	-0.25	0.10
genus..Eubacteriumeligensgroup.id.14372	genus	T2D	-0.27	0.12
genus..Eubacteriumhalliigroup.id.11338	genus	T2D	0.03	0.12
genus..Eubacteriumoxidoreducensgroup.id.11	genus	T2D	-0.06	0.12
genus..Eubacteriumxylanophilumgroup.id.143	genus	T2D	-0.30	0.12
genus.Hungatella.id.11306	genus	T2D	0.02	0.13
genus.Klebsiella.id.3507	genus	T2D	0.13	0.09
genus.LachnospiraceaeNC2004group.id.11316	genus	T2D	-0.26	0.12

© 2021 Chen Z et al. *JAMA Network Open.*

genus.LachnospiraceaeUCG008.id.11328	genus	T2D	-0.05	0.12
genus.unknowngenus.id.1868	genus	T2D	-0.23	0.09

© 2021 Chen Z et al. *JAMA Network Open.*

Taxa in LLD only	Clade	Phenotype	Beta_LLD	Se_LLD
domain.Archaea.id.2	Clade	T2D	0.15	0.22
phylum.Euryarchaeota.id.55	domain	T2D	0.15	0.22
phylum.Lentisphaerae.id.2238	phylum	T2D	-0.02	0.20
order.Actinomycetales.id.420	phylum	T2D	0.09	0.17
order.Mycoplasmatales.id.3946	order	T2D	-0.41	53.21
order.NB1.n.id.3953	order	T2D	0.09	0.16
order.Pseudomonadales.id.3709	order	T2D	-0.18	2.68
order.Spirochaetales.id.3870	order	T2D	-17.61	2014.59
order.Synergistales.id.3900	order	T2D	-0.96	1.66
order.Thermoplasmatales.id.220	order	T2D	0.21	0.09
order.unknownorder.id.1000000003	order	T2D	-0.27	0.68
order.Verrucomicrobiales.id.4030	order	T2D	0.09	0.23
order.Victivallales.id.2254	order	T2D	-0.02	0.20
order.Xanthomonadales.id.3786	order	T2D	-21.18	2344.91
class.Spirochaetes.id.3856	order	T2D	-17.61	2014.59
class.Synergistia.id.3899	class	T2D	-0.96	1.66
class.Thermoplasmata.id.210	class	T2D	0.21	0.09
family.Actinomycetaceae.id.421	class	T2D	0.09	0.17
family.BacteroidalesS24.7group.id.11173	family	T2D	-1.11	0.87
family.Christensenellaceae.id.1866	family	T2D	-0.24	0.35
family.FamilyXI.id.1936	family	T2D	-0.49	0.81
family.unknownfamily.id.1000000004	family	T2D	-0.27	0.68
family.unknownfamily.id.1000006161	family	T2D	0.09	0.16
family.unknownfamily.id.1855	family	T2D	0.13	0.08
family.vadinBE97.id.14446	family	T2D	-0.39	1.41
family.Veillonellaceae.id.2172	family	T2D	0.22	0.25
family.Xanthomonadaceae.id.3799	family	T2D	-21.18	2344.91
genus..Clostridiuminnocuumgroup.id.14397	family	T2D	0.17	0.12
genus..Eubacteriumbrachygroup.id.11296	family	T2D	0.39	0.14
genus.Abiotrophia.id.1803	genus	T2D	-12.52	953.72
genus.Actinomyces.id.423	genus	T2D	0.03	0.19

© 2021 Chen Z et al. *JAMA Network Open.*

genus.Actinotignum.id.11137	genus	T2D	-13.58	1197.75
genus.Allisonella.id.2174	genus	T2D	-0.27	0.36

© 2021 Chen Z et al. *JAMA Network Open.*

genus.Arcanobacterium.id.424	genus	T2D	-17.45	1669.68
genus.Asaccharobacter.id.813	genus	T2D	0.12	0.23
genus.Bilophila.id.3170	genus	T2D	0.19	0.23
genus.CandidatusMethanogranum.id.11110	genus	T2D	-4.21	436.67
genus.Catenisphaera.id.14395	genus	T2D	-807.94	70837.85
genus.Cellulosilyticum.id.1995	genus	T2D	-84.84	7287.37
genus.Cetobacterium.id.2209	genus	T2D	-0.43	53.21
genus.Cloacibacillus.id.3908	genus	T2D	-0.44	0.89
genus.Coprobacillus.id.2154	genus	T2D	-0.74	1.64
genus.CoriobacteriaceaeUCG.002.id.11158	genus	T2D	-0.29	0.57
genus.CoriobacteriaceaeUCG.003.id.11159	genus	T2D	-279.82	29054.73
genus.Denitrobacterium.id.818	genus	T2D	0.18	0.13
genus.dgA.11gutgroup.id.978	genus	T2D	-0.46	53.21
genus.Dielma.id.11380	genus	T2D	-0.16	1.79
genus.Epulopiscium.id.1998	genus	T2D	-2.20	3.28
genus.ErysipelotrichaceaeUCG006.id.11386	genus	T2D	-7.03	579.02
genus.ErysipelotrichaceaeUCG009.id.11389	genus	T2D	-0.49	53.21
genus.Escherichia.Shigella.id.3504	genus	T2D	0.11	0.14
genus.Eubacterium.id.1932	genus	T2D	0.27	0.20
genus.Faecalicoccus.id.11391	genus	T2D	-70.19	5031.35
genus.Faecalitalea.id.11392	genus	T2D	0.14	0.10
genus.Flavonifractor.id.2059	genus	T2D	-0.15	0.49
genus.Gardnerella.id.437	genus	T2D	-15.54	1509.59
genus.Gordonibacter.id.821	genus	T2D	-0.30	0.47
genus.Hafnia.Obesumbacterium.id.14636	genus	T2D	0.29	0.47
genus.Howardella.id.2000	genus	T2D	-0.54	0.43
genus.LachnospiraceaeNK3A20group.id.11318	genus	T2D	-2.52	318.19
genus.Lactococcus.id.1851	genus	T2D	-0.32	0.68
genus.Lautropia.id.2905	genus	T2D	-37.88	5179.50
genus.Leuconostoc.id.1841	genus	T2D	-0.86	0.85
genus.Methanobrevibacter.id.123	genus	T2D	0.18	0.22
genus.Methanosphaera.id.124	genus	T2D	-0.31	0.52

© 2021 Chen Z et al. *JAMA Network Open.*

genus.Mogibacterium.id.1960	genus	T2D	-2.14	1.60
genus.Morganella.id.3512	genus	T2D	-0.39	53.21

© 2021 Chen Z et al. *JAMA Network Open.*

genus.Porphyromonas.id.956	genus	T2D	-1.42	1.85
genus.Prevotella1.id.11179	genus	T2D	-1.07	156.52
genus.PrevotellaceaeUCG003.id.11187	genus	T2D	-57.99	7076.11
genus.PrevotellaceaeUCG004.id.11188	genus	T2D	-6.12	515.91
genus.Pseudomonas.id.3723	genus	T2D	-1029.29	83993.69
genus.Rikenella.id.973	genus	T2D	-15.17	1263.33
genus.RuminococcaceaeNK4A214group.id.113	genus	T2D	-0.28	0.41
genus.RuminococcaceaeUCG005.id.11363	genus	T2D	-0.11	0.29
genus.RuminococcaceaeUCG008.id.11365	genus	T2D	0.05	0.21
genus.RuminococcaceaeUCG010.id.11367	genus	T2D	0.05	0.27
genus.Sarcina.id.1896	genus	T2D	-52.89	5540.50
genus.Solobacterium.id.2161	genus	T2D	-3.26	4.06
genus.Staphylococcus.id.1780	genus	T2D	0.01	0.28
genus.Stenotrophomonas.id.3818	genus	T2D	-0.43	53.21
genus.Succinivibrio.id.3331	genus	T2D	-258.74	22480.30
genus.Synergistes.id.3913	genus	T2D	-25.30	2076.07
genus.Syntrophococcus.id.2016	genus	T2D	-0.89	89.40
genus.Treponema2.id.11577	genus	T2D	-15.36	1910.08
genus.Tyzzerella4.id.11336	genus	T2D	-3.64	8.45
genus.unknowngenus.id.1000000005	genus	T2D	-0.27	0.68
genus.unknowngenus.id.1000027565	genus	T2D	-0.39	1.41
genus.unknowngenus.id.1000043796	genus	T2D	0.13	0.08
genus.unknowngenus.id.1854	genus	T2D	-41.00	2997.88
genus.unknowngenus.id.2163	genus	T2D	0.15	0.11
genus.unknowngenus.id.3332	genus	T2D	-37.00	4333.76
genus.unknowngenus.id.441	genus	T2D	-0.10	0.38
genus.Ureaplasma.id.3951	genus	T2D	-0.41	53.21
genus.Varibaculum.id.427	genus	T2D	0.33	0.14
genus.Veillonella.id.2198	genus	T2D	0.15	0.15
genus.Weissella.id.1843	genus	T2D	0.09	0.13

© 2021 Chen Z et al. *JAMA Network Open.*

Supplementary Table 3 - Associations of taxa and type 2 diabetes

Effect estimates in RS and LLD were calculated using logistic regression; pooled estimates were calculated based on a mixed-effect meta-analysis.

Model 4: Adjusted for age, sex, Time in mail (RS), Batch (RS), smoking, education level (RS), physical activity, alcohol intake, total energy intake, BMI, PPI, and lipid-lowering medication.

Abbreviation RS, Rotterdam Study, LLD, Lifelines-Deep Study. P<0.0005 indicates statistical significance

Overlapping taxa in RS and LLD	Clade	Phenotype	Beta_RS	Se_RS	Beta_LLD	Se_LLD	OR_Meta	Lower95%CI_Meta	Upper95%CI_Meta	p_Meta	I^2
Bacteria.id.3	domain	T2D	-0.12	0.12	0.22	0.44	1.03	0.98	1.09	0.24	0.00
Actinobacteria.id.400	phylum	T2D	0.03	0.12	0.39	0.26	1.09	0.88	1.35	0.41	0.37
Bacteroidetes.id.905	phylum	T2D	0.00	0.12	-0.56	0.35	0.94	0.76	1.18	0.61	0.57
Cyanobacteria.id.1500	phylum	T2D	0.11	0.13	0.06	0.28	1.10	0.88	1.38	0.40	0.00
Firmicutes.id.1672	phylum	T2D	-0.09	0.12	-0.05	0.24	0.92	0.74	1.14	0.45	0.00
Proteobacteria.id.2375	phylum	T2D	0.06	0.12	0.03	0.17	1.05	0.87	1.28	0.61	0.00
Tenericutes.id.3919	phylum	T2D	-0.05	0.13	-0.09	0.35	0.95	0.75	1.19	0.63	0.00
Verrucomicrobia.id.3982	phylum	T2D	0.07	0.12	-0.10	0.26	1.04	0.84	1.28	0.70	0.00
Actinobacteria.id.419	class	T2D	0.06	0.13	0.37	0.25	1.13	0.91	1.41	0.26	0.18
Alphaproteobacteria.id.2379	class	T2D	0.05	0.13	-0.21	0.43	1.03	0.81	1.30	0.83	0.00
Bacilli.id.1673	class	T2D	0.21	0.13	0.22	0.18	1.24	1.01	1.51	0.04	0.00
Bacteroidia.id.912	class	T2D	0.00	0.12	-0.57	0.35	0.94	0.76	1.18	0.61	0.58
Betaproteobacteria.id.2867	class	T2D	0.12	0.12	-0.01	0.29	1.11	0.89	1.38	0.37	0.00
Clostridia.id.1859	class	T2D	-0.16	0.11	-0.08	0.23	0.87	0.71	1.06	0.17	0.00
Coriobacteriia.id.809	class	T2D	-0.04	0.12	0.15	0.26	0.99	0.80	1.23	0.94	0.00
Deltaproteobacteria.id.3087	class	T2D	0.17	0.12	-0.01	0.31	1.16	0.93	1.45	0.19	0.00
Erysipelotrichia.id.2147	class	T2D	-0.09	0.12	-0.28	0.28	0.89	0.71	1.10	0.28	0.00
Gammaproteobacteria.id.3303	class	T2D	0.03	0.13	0.03	0.16	1.03	0.85	1.25	0.76	0.00
Melainabacteria.id.1589	class	T2D	0.07	0.13	0.09	0.25	1.07	0.86	1.34	0.53	0.00
Mollicutes.id.3920	class	T2D	-0.05	0.13	-0.09	0.35	0.95	0.75	1.19	0.63	0.00
Negativicutes.id.2164	class	T2D	-0.08	0.12	0.13	0.29	0.95	0.77	1.17	0.63	0.00
Verrucomicrobiae.id.4029	class	T2D	0.07	0.12	-0.10	0.26	1.04	0.85	1.29	0.68	0.00
Aeromonadales.id.3316	order	T2D	0.08	0.12	-412.55	39640.87	1.08	0.86	1.35	0.51	0.00
Bacteroidales.id.913	order	T2D	0.00	0.12	-0.57	0.35	0.94	0.76	1.18	0.61	0.58
Bifidobacteriales.id.432	order	T2D	0.02	0.12	0.37	0.25	1.09	0.88	1.36	0.42	0.33
Burkholderiales.id.2874	order	T2D	0.12	0.12	-0.02	0.29	1.11	0.89	1.38	0.37	0.00
Clostridiales.id.1863	order	T2D	-0.16	0.11	-0.08	0.23	0.87	0.71	1.06	0.17	0.00
Coriobacteriales.id.810	order	T2D	-0.04	0.12	0.15	0.26	0.99	0.80	1.23	0.94	
Desulfovibrionales.id.3156	order	T2D	0.17	0.12	0.00	0.31	1.16	0.93	1.45	0.19	

© 2021 Chen Z et al. *JAMA Network Open*.

0.00

0.00

© 2021 Chen Z et al. *JAMA Network Open.*

Enterobacteriales.id.3468	order	T2D	0.04	0.13	0.10	0.16	1.06	0.87	1.29	0.54	0.00
Erysipelotrichales.id.2148	order	T2D	-0.09	0.12	-0.28	0.28	0.89	0.71	1.10	0.28	0.00
Gastranaerophilales.id.1591	order	T2D	0.07	0.13	0.09	0.25	1.07	0.86	1.35	0.53	0.00
Lactobacillales.id.1800	order	T2D	0.20	0.13	0.22	0.18	1.23	1.01	1.50	0.04	0.00
MollicutesRF9.id.11579	order	T2D	-0.04	0.12	-0.10	0.35	0.95	0.76	1.20	0.67	0.00
Pasteurellales.id.3688	order	T2D	-0.11	0.13	0.17	0.17	0.99	0.81	1.21	0.91	0.40
Rhodospirillales.id.2667	order	T2D	0.06	0.12	-0.17	0.40	1.04	0.82	1.31	0.74	0.00
Selenomonadales.id.2165	order	T2D	-0.08	0.12	0.13	0.29	0.95	0.77	1.17	0.63	0.00
Verrucomicrobiales.id.4030	order	T2D	0.07	0.12	-0.10	0.26	1.04	0.85	1.29	0.68	0.00
Acidaminococcaceae.id.2166	family	T2D	-0.11	0.12	-1.29	0.72	0.87	0.69	1.09	0.23	0.62
Alcaligenaceae.id.2875	family	T2D	0.11	0.12	-0.05	0.30	1.09	0.87	1.37	0.44	0.00
Bacteroidaceae.id.917	family	T2D	0.08	0.13	-0.54	0.36	1.01	0.80	1.28	0.93	0.61
Bifidobacteriaceae.id.433	family	T2D	0.02	0.12	0.37	0.25	1.09	0.88	1.36	0.42	0.33
Christensenellaceae.id.1866	family	T2D	-0.24	0.11	-0.06	0.35	0.80	0.65	0.99	0.04	0.00
Clostridiaceae1.id.1869	family	T2D	-0.88	0.14	0.07	0.26	0.51	0.41	0.65	0.00	0.91
ClostridialesvadinBB60group.id.11286	family	T2D	-0.03	0.13	0.12	0.19	1.02	0.82	1.26	0.88	0.00
Coriobacteriaceae.id.811	family	T2D	-0.04	0.12	0.15	0.26	0.99	0.80	1.23	0.94	0.00
Desulfovibrionaceae.id.3169	family	T2D	0.17	0.12	-0.01	0.31	1.16	0.93	1.45	0.19	0.00
Enterobacteriaceae.id.3469	family	T2D	0.04	0.13	0.10	0.16	1.06	0.87	1.29	0.54	0.00
Enterococcaceae.id.1828	family	T2D	0.02	0.10	-1.27	2.42	1.02	0.84	1.25	0.83	0.00
Erysipelotrichaceae.id.2149	family	T2D	-0.09	0.12	-0.28	0.28	0.89	0.71	1.10	0.28	0.00
FamilyXIII.id.1957	family	T2D	-0.14	0.11	-0.37	0.32	0.85	0.69	1.04	0.12	0.00
Lachnospiraceae.id.1987	family	T2D	0.09	0.13	-0.28	0.28	1.03	0.82	1.29	0.82	0.28
Lactobacillaceae.id.1836	family	T2D	0.18	0.11	0.01	0.14	1.12	0.94	1.33	0.19	0.00
Pasteurellaceae.id.3689	family	T2D	-0.11	0.13	0.17	0.17	0.99	0.81	1.21	0.91	0.40
Peptococcaceae.id.2024	family	T2D	-0.15	0.12	-0.45	0.56	0.85	0.67	1.08	0.18	0.00
Peptostreptococcaceae.id.2042	family	T2D	-0.65	0.12	-0.11	0.29	0.56	0.45	0.70	0.00	0.65
Porphyromonadaceae.id.943	family	T2D	-0.05	0.12	-0.57	0.36	0.90	0.72	1.13	0.37	0.45
Prevotellaceae.id.960	family	T2D	0.03	0.12	-0.31	0.45	1.00	0.80	1.26	0.98	0.00
Rhodospirillaceae.id.2717	family	T2D	0.06	0.12	-0.17	0.40	1.04	0.82	1.32	0.73	0.00
Rikenellaceae.id.967	family	T2D	-0.04	0.12	0.07	0.28	0.98	0.79	1.22	0.86	0.00
Ruminococcaceae.id.2050	family	T2D	-0.10	0.11	0.33	0.29	0.95	0.78	1.16	0.63	0.47

© 2021 Chen Z et al. *JAMA Network Open.*

Streptococcaceae.id.1850	family	T2D	0.18	0.12	0.26	0.18	1.23	1.01	1.50	0.04	0.00
Succinivibrionaceae.id.3326	family	T2D	0.08	0.12	-412.58	39666.32	1.08	0.86	1.35	0.51	0.00

© 2021 Chen Z et al. *JAMA Network Open.*

Veillonellaceae.id.2172	family	T2D	-0.10	0.12	0.20	0.28	0.95	0.76	1.18	0.64	0.00
Verrucomicrobiaceae.id.4036	family	T2D	0.07	0.12	-0.10	0.26	1.04	0.85	1.29	0.68	0.00
unknownfamily.id.1000001214	family	T2D	0.07	1.07	0.09	0.25	1.07	0.86	1.35	0.53	0.00
unknownfamily.id.1000005471	family	T2D	-0.04	0.96	-0.10	0.35	0.95	0.76	1.20	0.67	0.00
unknownfamily.id.987	family	T2D	-0.13	0.88	-0.83	1.18	0.87	0.68	1.12	0.28	0.00
Bacteroidespectinophilusgroup.id.14371	genus	T2D	-0.84	0.62	-0.13	0.55	0.64	0.29	1.44	0.28	0.00
Eubacteriumcoprostanoligenesgroup.id.11375	genus	T2D	-0.20	0.11	-0.25	0.32	0.82	0.67	0.99	0.04	0.00
Eubacteriumeligensgroup.id.14372	genus	T2D	-0.27	0.12	0.20	0.18	0.88	0.72	1.07	0.21	0.79
Eubacteriumhalliigroup.id.11338	genus	T2D	0.01	0.12	-0.37	0.30	0.96	0.77	1.19	0.71	0.26
Eubacteriumrectalegroup.id.14374	genus	T2D	0.06	0.12	0.11	0.16	1.09	0.90	1.31	0.40	0.00
Eubacteriumruminantiumgroup.id.11340	genus	T2D	-0.07	0.13	0.21	0.20	1.01	0.82	1.25	0.92	0.25
Eubacteriumventriosumgroup.id.11341	genus	T2D	-0.08	0.12	0.01	0.27	0.94	0.75	1.16	0.55	0.00
Eubacteriumxylanophilumgroup.id.14375	genus	T2D	-0.18	0.13	-0.01	0.30	0.86	0.68	1.08	0.19	0.00
Ruminococcusgauvreauiigroup.id.11342	genus	T2D	-0.19	0.12	-1.08	0.51	0.79	0.63	0.98	0.04	0.66
Ruminococcusgnavusgroup.id.14376	genus	T2D	-0.06	0.12	-0.35	0.50	0.93	0.74	1.16	0.51	0.00
Ruminococcustorquesgroup.id.14377	genus	T2D	0.19	0.12	-0.63	0.45	1.14	0.90	1.44	0.28	0.67
Acidaminococcus.id.2167	genus	T2D	0.06	0.10	-3.99	5.72	1.06	0.86	1.30	0.58	0.00
Adlercreutzia.id.812	genus	T2D	0.02	0.12	-0.04	0.28	1.01	0.82	1.25	0.91	0.00
Akkermansia.id.4037	genus	T2D	0.07	0.12	-0.10	0.26	1.04	0.85	1.29	0.68	0.00
Alistipes.id.968	genus	T2D	0.03	0.12	0.00	0.30	1.02	0.82	1.28	0.83	0.00
Alloprevotella.id.961	genus	T2D	-0.05	0.13	-2.12	3.72	0.95	0.74	1.23	0.71	0.00
Anaerostipes.id.1991	genus	T2D	-0.10	0.12	-0.09	0.27	0.90	0.73	1.11	0.34	0.00
Anaerotruncus.id.2054	genus	T2D	-0.20	0.12	-0.11	0.33	0.83	0.67	1.03	0.08	0.00
Bacteroides.id.918	genus	T2D	0.08	0.13	-0.54	0.36	1.01	0.80	1.28	0.93	0.61
Barnesiella.id.944	genus	T2D	-0.01	0.12	-0.68	0.47	0.95	0.75	1.20	0.67	0.47
Bifidobacterium.id.436	genus	T2D	0.02	0.12	0.36	0.25	1.09	0.88	1.36	0.42	0.30
Blautia.id.1992	genus	T2D	0.21	0.13	-0.56	0.36	1.13	0.89	1.43	0.32	0.75
Butyricicoccus.id.2055	genus	T2D	0.02	0.12	-0.70	0.49	0.98	0.78	1.23	0.86	0.50
Butyricimonas.id.945	genus	T2D	0.19	0.12	-0.14	0.45	1.18	0.93	1.49	0.17	0.00
Butyrivibrio.id.1993	genus	T2D	-0.05	0.13	-0.45	0.71	0.94	0.73	1.21	0.61	0.00
Catenibacterium.id.2153	genus	T2D	-0.05	0.11	-1.45	1.55	0.94	0.75	1.18	0.59	0.00
ChristensenellaceaeR7group.id.11283	genus	T2D	-0.23	0.11	-0.04	0.35	0.81	0.66	1.00	0.05	0.00

© 2021 Chen Z et al. *JAMA Network Open.*

Clostridiumsensustricto1.id.1873	genus	T2D	-0.88	0.14	0.08	0.26	0.51	0.40	0.65	0.00	0.90
Collinsella.id.815	genus	T2D	0.00	0.12	0.26	0.23	1.06	0.85	1.31	0.61	0.02

© 2021 Chen Z et al. *JAMA Network Open.*

Coprococcus1.id.11301	genus	T2D	0.09	0.12	0.18	0.26	1.11	0.90	1.36	0.34	0.00
Coprococcus2.id.11302	genus	T2D	-0.13	0.12	0.00	0.32	0.90	0.71	1.12	0.34	0.00
Coprococcus3.id.11303	genus	T2D	0.07	0.12	-0.44	0.35	1.01	0.81	1.27	0.90	0.47
Desulfovibrio.id.3173	genus	T2D	0.06	0.12	-0.13	0.37	1.04	0.83	1.31	0.71	0.00
Dialister.id.2183	genus	T2D	-0.07	0.12	0.24	0.27	0.98	0.79	1.23	0.89	0.11
Dorea.id.1997	genus	T2D	-0.11	0.11	-0.09	0.29	0.90	0.73	1.10	0.29	0.00
Eggerthella.id.819	genus	T2D	0.13	0.12	-1.96	1.30	1.12	0.89	1.42	0.33	0.61
Eisenbergiella.id.11304	genus	T2D	0.10	0.11	0.13	0.24	1.11	0.91	1.36	0.31	0.00
Enterococcus.id.1831	genus	T2D	0.01	0.10	-6.24	7.70	1.01	0.83	1.23	0.93	0.00
Enterorhabdus.id.820	genus	T2D	0.07	0.12	-0.27	0.38	1.04	0.83	1.30	0.74	0.00
Erysipelatoclostridium.id.11381	genus	T2D	-0.05	0.12	0.19	0.17	1.03	0.85	1.24	0.80	0.22
ErysipelotrichaceaeUCG003.id.11384	genus	T2D	-0.10	0.12	-0.12	0.34	0.91	0.73	1.13	0.37	0.00
Faecalibacterium.id.2057	genus	T2D	-0.08	0.11	-0.04	0.28	0.93	0.76	1.12	0.43	0.00
FamilyXIIIAD3011group.id.11293	genus	T2D	-0.06	0.12	-0.80	0.50	0.90	0.72	1.13	0.38	0.51
FamilyXIIIUCG001.id.11294	genus	T2D	-0.09	0.12	-0.05	0.28	0.92	0.74	1.14	0.46	0.00
Fusicatenibacter.id.11305	genus	T2D	-0.18	0.11	0.13	0.20	0.90	0.74	1.09	0.28	0.44
Haemophilus.id.3698	genus	T2D	-0.11	0.13	0.16	0.17	0.99	0.81	1.21	0.92	0.37
Holdemanella.id.11393	genus	T2D	0.07	0.12	-0.08	0.25	1.04	0.84	1.28	0.71	0.00
Intestinibacter.id.11345	genus	T2D	-0.70	0.13	0.06	0.23	0.60	0.48	0.76	0.00	0.88
Intestinimonas.id.2062	genus	T2D	-0.05	0.11	0.19	0.23	1.00	0.82	1.22	0.98	0.00
Lachnoclostridium.id.11308	genus	T2D	0.03	0.12	-0.19	0.22	0.98	0.80	1.21	0.86	0.00
Lachnospira.id.2004	genus	T2D	-0.22	0.12	-0.22	0.38	0.80	0.65	1.00	0.05	0.00
LachnospiraceaeFCS020group.id.11314	genus	T2D	-0.07	0.12	0.22	0.21	1.00	0.82	1.22	0.99	0.34
LachnospiraceaeND3007group.id.11317	genus	T2D	-0.28	0.12	0.39	0.23	0.87	0.71	1.06	0.16	0.85
LachnospiraceaeNK4A136group.id.11319	genus	T2D	-0.25	0.12	-0.11	0.26	0.80	0.65	0.99	0.04	0.00
LachnospiraceaeUCG001.id.11321	genus	T2D	0.15	0.12	0.06	0.21	1.13	0.92	1.39	0.23	0.00
LachnospiraceaeUCG004.id.11324	genus	T2D	-0.01	0.12	-0.61	0.55	0.97	0.76	1.22	0.78	0.13
LachnospiraceaeUCG010.id.11330	genus	T2D	0.14	0.12	-0.29	0.46	1.12	0.88	1.41	0.36	0.00
Lactobacillus.id.1837	genus	T2D	0.16	0.11	-0.02	0.16	1.10	0.92	1.32	0.29	0.00
Marvinbryantia.id.2005	genus	T2D	-0.02	0.12	-0.49	0.38	0.94	0.76	1.18	0.60	0.30
Megamonas.id.2184	genus	T2D	-0.04	0.12	-798.63	67821.78	0.96	0.75	1.21	0.71	0.00
Megasphaera.id.2185	genus	T2D	-0.15	0.14	-0.48	0.86	0.85	0.66	1.11	0.24	0.00

© 2021 Chen Z et al. *JAMA Network Open.*

Mitsuokella.id.2186	genus	T2D	-0.15	0.12	-0.09	0.47	0.86	0.68	1.09	0.22	0.00
Odoribacter.id.952	genus	T2D	0.18	0.12	-0.10	0.32	1.15	0.92	1.45	0.23	0.00

© 2021 Chen Z et al. *JAMA Network Open.*

Olsenella.id.822	genus	T2D	0.07	0.12	-8.29	6.71	1.07	0.84	1.35	0.58	0.36
Oscillospira.id.2064	genus	T2D	-0.09	0.12	0.08	0.29	0.94	0.75	1.17	0.58	0.00
Parabacteroides.id.954	genus	T2D	-0.06	0.13	-0.52	0.37	0.90	0.71	1.13	0.37	0.30
Paraprevotella.id.962	genus	T2D	-0.04	0.12	-0.09	0.39	0.96	0.76	1.20	0.70	0.00
Parasutterella.id.2892	genus	T2D	0.05	0.12	0.11	0.23	1.07	0.87	1.31	0.54	0.00
Peptococcus.id.2037	genus	T2D	-0.10	0.12	-0.55	0.61	0.89	0.70	1.12	0.32	0.00
Phascolarctobacterium.id.2168	genus	T2D	-0.11	0.12	-1.07	0.65	0.87	0.69	1.09	0.23	0.52
Prevotella2.id.11180	genus	T2D	0.10	0.12	-369.75	32035.52	1.11	0.88	1.40	0.39	0.00
Prevotella7.id.11182	genus	T2D	-0.01	0.13	-0.13	0.31	0.97	0.77	1.23	0.82	0.00
Prevotella9.id.11183	genus	T2D	-0.03	0.12	-0.19	0.41	0.95	0.76	1.20	0.68	0.00
PrevotellaceaeNK3B31group.id.11185	genus	T2D	-0.05	0.12	-0.23	0.83	0.95	0.75	1.21	0.68	0.00
PrevotellaceaeUCG001.id.11186	genus	T2D	-0.02	0.12	-0.93	3.37	0.97	0.76	1.24	0.83	0.00
RikenellaceaeRC9gutgroup.id.11191	genus	T2D	-0.06	0.12	0.21	0.18	1.02	0.84	1.25	0.83	0.30
Romboutsia.id.11347	genus	T2D	-0.58	0.12	-0.82	0.61	0.55	0.44	0.70	0.00	0.00
Roseburia.id.2012	genus	T2D	-0.03	0.11	-0.49	0.36	0.93	0.75	1.15	0.50	0.30
Ruminiclostridium5.id.11355	genus	T2D	-0.27	0.12	0.13	0.19	0.85	0.70	1.03	0.10	0.71
Ruminiclostridium6.id.11356	genus	T2D	-0.17	0.12	0.05	0.27	0.87	0.70	1.09	0.23	0.00
Ruminiclostridium9.id.11357	genus	T2D	-0.04	0.12	-0.16	0.27	0.95	0.76	1.17	0.61	0.00
RuminococcaceaeNK4A214group.id.11358	genus	T2D	-0.19	0.12	-0.26	0.38	0.82	0.66	1.02	0.08	0.00
RuminococcaceaeUCG002.id.11360	genus	T2D	-0.08	0.12	-0.07	0.33	0.92	0.74	1.15	0.46	0.00
RuminococcaceaeUCG003.id.11361	genus	T2D	0.01	0.13	0.03	0.32	1.01	0.80	1.27	0.94	0.00
RuminococcaceaeUCG004.id.11362	genus	T2D	0.01	0.12	-0.08	0.27	1.00	0.80	1.24	0.99	0.00
RuminococcaceaeUCG005.id.11363	genus	T2D	-0.13	0.12	-0.10	0.31	0.89	0.71	1.10	0.27	0.00
RuminococcaceaeUCG008.id.11365	genus	T2D	-0.08	0.13	0.08	0.23	0.96	0.77	1.19	0.68	0.00
RuminococcaceaeUCG010.id.11367	genus	T2D	-0.17	0.13	0.12	0.28	0.89	0.71	1.12	0.31	0.00
RuminococcaceaeUCG013.id.11370	genus	T2D	-0.13	0.12	-0.33	0.28	0.86	0.69	1.06	0.15	0.00
RuminococcaceaeUCG014.id.11371	genus	T2D	0.00	0.12	0.21	0.24	1.04	0.84	1.28	0.71	0.00
Ruminococcus1.id.11373	genus	T2D	-0.04	0.12	0.10	0.25	0.99	0.80	1.22	0.92	0.00
Ruminococcus2.id.11374	genus	T2D	0.01	0.12	0.28	0.27	1.05	0.86	1.30	0.62	0.00
Sellimonas.id.14369	genus	T2D	-0.15	0.14	-0.17	0.19	0.86	0.69	1.06	0.16	0.00
Senegalimassilia.id.11160	genus	T2D	-0.10	0.12	0.24	0.19	0.99	0.81	1.22	0.96	0.55
Slackia.id.825	genus	T2D	-0.20	0.13	-0.40	0.40	0.81	0.64	1.02	0.07	0.00

© 2021 Chen Z et al. *JAMA Network Open.*

Streptococcus.id.1853	genus	T2D	0.18	0.12	0.26	0.18	1.23	1.01	1.50	0.04	0.00
Subdoligranulum.id.2070	genus	T2D	0.00	0.12	0.34	0.23	1.07	0.87	1.31	0.52	0.44

© 2021 Chen Z et al. *JAMA Network Open.*

Succiniclasticum.id.2169	genus	T2D	0.03	0.13	-295.78	37207.61	1.03	0.79	1.34	0.82	0.00
Sutterella.id.2896	genus	T2D	0.07	0.13	-0.86	0.77	1.04	0.82	1.33	0.74	0.29
Terrisporobacter.id.11348	genus	T2D	-0.43	0.14	0.19	0.13	0.90	0.75	1.09	0.27	0.91
Turicibacter.id.2162	genus	T2D	-0.31	0.13	0.11	0.22	0.81	0.65	1.01	0.07	0.62
Tyzzerella3.id.11335	genus	T2D	0.14	0.11	-127.52	10056.91	1.15	0.92	1.44	0.21	0.00
Veillonella.id.2198	genus	T2D	-0.02	0.12	0.11	0.16	1.02	0.85	1.23	0.80	0.00
unknowngenus.id.1000000073	genus	T2D	-0.03	0.97	0.12	0.19	1.02	0.82	1.26	0.88	0.00
unknowngenus.id.1000001215	genus	T2D	0.07	1.07	0.09	0.25	1.07	0.86	1.35	0.53	0.00
unknowngenus.id.1000005472	genus	T2D	-0.04	0.96	-0.10	0.35	0.95	0.76	1.20	0.67	0.00
unknowngenus.id.1000005479	genus	T2D	-0.05	0.95	-0.65	0.73	0.94	0.73	1.19	0.59	0.00
unknowngenus.id.1000013899	genus	T2D	-0.13	0.88	-0.83	1.18	0.87	0.68	1.12	0.28	0.00
unknowngenus.id.2071	genus	T2D	-0.18	0.84	0.00	0.22	0.88	0.71	1.09	0.23	0.00
unknowngenus.id.2755	genus	T2D	0.06	1.06	-0.20	0.42	1.04	0.82	1.31	0.75	0.00
unknowngenus.id.826	genus	T2D	-0.01	0.99	-0.19	0.31	0.97	0.77	1.21	0.77	0.00
unknowngenus.id.964	genus	T2D	-0.02	0.98	-221.95	24315.54	0.98	0.75	1.28	0.89	0.00

**Taxa in RS only**	**Clade**	**Phenotype**	**Beta_RS**	**Se_RS**
family.BacteroidalesS247group.id.11173	family	T2D	-0.05	0.13
family.Veillonellaceae.id.2172	family	T2D	-0.10	0.12
genus..Bacteroidespectinophilusgroup.id.1437	family	T2D	-0.84	0.62
genus.Enterobacter.id.3502	genus	T2D	0.08	0.12
genus.EscherichiaShigella.id.3504	genus	T2D	-0.04	0.13
genus..Eubacteriumcoprostanoligenesgroup.id	genus	T2D	-0.20	0.11
genus..Eubacteriumeligensgroup.id.14372	genus	T2D	-0.27	0.12
genus..Eubacteriumhalliigroup.id.11338	genus	T2D	0.01	0.12
genus..Eubacteriumoxidoreducensgroup.id.11	genus	T2D	-0.02	0.12
genus..Eubacteriumxylanophilumgroup.id.143	genus	T2D	-0.18	0.13
genus.Hungatella.id.11306	genus	T2D	-0.01	0.13
genus.Klebsiella.id.3507	genus	T2D	0.08	0.10
genus.LachnospiraceaeNC2004group.id.11316	genus	T2D	-0.22	0.12

© 2021 Chen Z et al. *JAMA Network Open.*

genus.LachnospiraceaeUCG008.id.11328	genus	T2D	-0.05	0.12
genus.unknowngenus.id.1868	genus	T2D	-0.10	0.12

© 2021 Chen Z et al. *JAMA Network Open.*

Taxa in LLD only	Clade	Phenotype	Beta_LLD	Se_LLD
domain.Archaea.id.2	Clade	T2D	0.17	0.22
phylum.Euryarchaeota.id.55	domain	T2D	0.17	0.22
phylum.Lentisphaerae.id.2238	phylum	T2D	0.03	0.19
order.Actinomycetales.id.420	phylum	T2D	0.10	0.18
order.Mycoplasmatales.id.3946	order	T2D	-0.39	53.21
order.NB1.n.id.3953	order	T2D	0.12	0.17
order.Pseudomonadales.id.3709	order	T2D	-0.27	4.82
order.Spirochaetales.id.3870	order	T2D	-18.00	1841.03
order.Synergistales.id.3900	order	T2D	-0.58	1.54
order.Thermoplasmatales.id.220	order	T2D	0.19	0.10
order.unknownorder.id.1000000003	order	T2D	-0.17	0.59
order.Verrucomicrobiales.id.4030	order	T2D	-27.41	68.42
order.Victivallales.id.2254	order	T2D	717.20	4624.75
order.Xanthomonadales.id.3786	order	T2D	-632578.86	77911496.93
class.Spirochaetes.id.3856	order	T2D	-956376.20	97828537.00
class.Synergistia.id.3899	class	T2D	-16550.50	43744.66
class.Thermoplasmata.id.210	class	T2D	23222.28	11796.78
family.Actinomycetaceae.id.421	class	T2D	636.93	1111.03
family.BacteroidalesS24.7group.id.11173	family	T2D	-416.06	469.83
family.Christensenellaceae.id.1866	family	T2D	-35.01	202.79
family.FamilyXI.id.1936	family	T2D	-5834.52	8493.48
family.unknownfamily.id.1000000004	family	T2D	-1961.30	6964.64
family.unknownfamily.id.1000006161	family	T2D	2624.03	3550.43
family.unknownfamily.id.1855	family	T2D	506.51	331.01
family.vadinBE97.id.14446	family	T2D	-7888.71	33513.17
family.Veillonellaceae.id.2172	family	T2D	43.39	59.81
family.Xanthomonadaceae.id.3799	family	T2D	-632578.86	77911496.93
genus..Clostridiuminnocuumgroup.id.14397	family	T2D	335.52	949.03
genus..Eubacteriumbrachygroup.id.11296	family	T2D	1848.96	873.58
genus.Abiotrophia.id.1803	genus	T2D	-975887.47	112721664.6
genus.Actinomyces.id.423	genus	T2D	-39212.65	56207.71 2
genus.Actinotignum.id.11137	genus	T2D	-826750.05	109361350.9
genus.Allisonella.id.2174	genus	T2D	-4193.58	6365.41

© 2021 Chen Z et al. *JAMA Network Open.*

9

© 2021 Chen Z et al. *JAMA Network Open.*

genus.Arcanobacterium.id.424	genus	T2D	-969578.65	85708848.26
genus.Asaccharobacter.id.813	genus	T2D	859.56	2358.04
genus.Bilophila.id.3170	genus	T2D	1621.61	1835.24
genus.CandidatusMethanogranum.id.11110	genus	T2D	-816043.57	94649879.58
genus.Catenisphaera.id.14395	genus	T2D	-979626.26	86502192.01
genus.Cellulosilyticum.id.1995	genus	T2D	-918976.00	80526402.25
genus.Cetobacterium.id.2209	genus	T2D	-224661.26	25470189.88
genus.Cloacibacillus.id.3908	genus	T2D	-12663.56	38283.89
genus.Coprobacillus.id.2154	genus	T2D	-6768.62	17082.33
genus.CoriobacteriaceaeUCG.002.id.11158	genus	T2D	-7714.46	16344.32
genus.CoriobacteriaceaeUCG.003.id.11159	genus	T2D	-901700.07	94003494.98
genus.Denitrobacterium.id.818	genus	T2D	4375.35	2627.08
genus.dgA.11gutgroup.id.978	genus	T2D	-164915.59	20376297.83
genus.Dielma.id.11380	genus	T2D	-2576.00	35410.43
genus.Epulopiscium.id.1998	genus	T2D	-8406.69	18727.22
genus.ErysipelotrichaceaeUCG006.id.11386	genus	T2D	-938246.56	73676190.55
genus.ErysipelotrichaceaeUCG009.id.11389	genus	T2D	-76702.17	8490547.39
genus.Escherichia.Shigella.id.3504	genus	T2D	49.71	102.98
genus.Eubacterium.id.1932	genus	T2D	3026.60	2853.80
genus.Faecalicoccus.id.11391	genus	T2D	-1008344.10	74862785.76
genus.Faecalitalea.id.11392	genus	T2D	389.04	412.06
genus.Flavonifractor.id.2059	genus	T2D	-9983.06	8626.36
genus.Gardnerella.id.437	genus	T2D	-991197.47	91596782.21
genus.Gordonibacter.id.821	genus	T2D	-3216.79	3980.14
genus.Hafnia.Obesumbacterium.id.14636	genus	T2D	5464.04	6963.80
genus.Howardella.id.2000	genus	T2D	-14045.62	10452.20
genus.LachnospiraceaeNK3A20group.id.11318	genus	T2D	-58692.31	7722398.64
genus.Lactococcus.id.1851	genus	T2D	-1238.62	3296.06
genus.Lautropia.id.2905	genus	T2D	-704124.14	97309311.93
genus.Leuconostoc.id.1841	genus	T2D	-12611.55	13776.11
genus.Methanobrevibacter.id.123	genus	T2D	66.48	72.36
genus.Methanosphaera.id.124	genus	T2D	-378.87	786.50

© 2021 Chen Z et al. *JAMA Network Open.*

genus.Mogibacterium.id.1960	genus	T2D	-4650.00	5006.80
genus.Morganella.id.3512	genus	T2D	-200910.72	25470194.81

© 2021 Chen Z et al. *JAMA Network Open.*

genus.Porphyromonas.id.956	genus	T2D	-67060.31	80424.85
genus.Prevotella1.id.11179	genus	T2D	-66010.75	9870113.56
genus.PrevotellaceaeUCG003.id.11187	genus	T2D	-838943.84	104622861.12
genus.PrevotellaceaeUCG004.id.11188	genus	T2D	-871625.55	113815325.59
genus.Pseudomonas.id.3723	genus	T2D	-1003764.38	75177987.73
genus.Rikenella.id.973	genus	T2D	-881182.24	116205322.14
genus.RuminococcaceaeNK4A214group.id.113	genus	T2D	-384.70	570.08
genus.RuminococcaceaeUCG005.id.11363	genus	T2D	-177.05	565.10
genus.RuminococcaceaeUCG008.id.11365	genus	T2D	211.84	636.44
genus.RuminococcaceaeUCG010.id.11367	genus	T2D	494.92	1164.56
genus.Sarcina.id.1896	genus	T2D	-717210.15	74568532.44
genus.Solobacterium.id.2161	genus	T2D	-4975.45	7793.50
genus.Staphylococcus.id.1780	genus	T2D	-1109.94	7560.90
genus.Stenotrophomonas.id.3818	genus	T2D	-15598.26	1727470.73
genus.Succinivibrio.id.3331	genus	T2D	-895534.84	80325652.35
genus.Synergistes.id.3913	genus	T2D	-819843.93	74178755.63
genus.Syntrophococcus.id.2016	genus	T2D	-88186.74	8647650.41
genus.Treponema2.id.11577	genus	T2D	-791767.74	107821373.84
genus.Tyzzerella4.id.11336	genus	T2D	-9763.48	28105.36
genus.unknowngenus.id.1000000005	genus	T2D	-1961.30	6964.64
genus.unknowngenus.id.1000027565	genus	T2D	-7888.71	33513.17
genus.unknowngenus.id.1000043796	genus	T2D	506.51	331.01
genus.unknowngenus.id.1854	genus	T2D	-1082778.03	117792911.74
genus.unknowngenus.id.2163	genus	T2D	3043.76	2539.31
genus.unknowngenus.id.3332	genus	T2D	-946963.07	107653357.20
genus.unknowngenus.id.441	genus	T2D	-795.45	4893.94
genus.Ureaplasma.id.3951	genus	T2D	-148560.76	20376295.13
genus.Varibaculum.id.427	genus	T2D	5314.59	2616.19
genus.Veillonella.id.2198	genus	T2D	959.99	1483.28
genus.Weissella.id.1843	genus	T2D	1024.17	1608.10

© 2021 Chen Z et al. *JAMA Network Open.*

eTable 4. Associations of a and β Diversity With Insulin Resistance and Type 2 Diabetes After Additionally Adjusting for Diet Quality and Blood Pressure

	HOMA-IR	T2D
	**β (95% CI)**	**OR (95% CI)**
**Shannon index**	-0.05 (-0.08, -0.02)	0.85 (0.70, 1.04)
**Richness**	-0.06 (-0.10, -0.02)	0.89 (0.79, 1.00)
**Inverse Simpson index**	-0.03 (-0.07, 0.01)	0.95 (0.86, 1.05)
	**R^2 (p value)**	**R^2 (p value)**
**Bray-Curtis distance**	0.004 (0.001)	0.003 (0.001)

β coefficients and 95% confidence intervals (CI) were from linear regression models for associations between Shannon index, richness, and Inverse Simpson index with HOMA-IR. OR and 95% CI were from logistical regression models for associations between Shannon index, Richness, and Inverse Simpson with T2D. R^2 and p value were examined using permutation analysis of variance (PERMANOVA, 1,000 permutations) for associations of beta-diversity (Bray-Curtis distance). These analyses were conducted in the Rotterdam Study and adjusted for age, sex, time in mail, Batch, smoking education, physical activity, alcohol intake, energy intake, BMI, lipid-lowering medication, PPI, blood pressure, and diet quality score.

eTable 5. Statistically Significant Associations Between Taxa and Insulin Resistance After Additionally Adjusting for Diet Quality and Blood Pressure

**Taxa**	**HOMA-IR β (95% CI)**	**P values**
family.ClostridialesvadinBB60group.id.11286	-0.08 (-0.12, -0.04)	P<0.0001
family.*Christensenellaceae.id.1866*	-0.09 (-0.14, -0.03)	P<0.0001
genus. *ChristensenellaceaeR7group.id.11283*	-0.09 (-0.15, -0.03)	P<0.0001
genus.*RuminococcaceaeUCG.005.id.11363*	-0.07 (-0.11, -0.04)	P<0.0001
genus.*RuminococcaceaeUCG.008.id.11365*	-0.08 (-0.12, -0.04)	P<0.0001
genus.*RuminococcaceaeUCG.010.id.11367*	-0.07 (-0.10, -0.04)	P<0.0001
genus.*RuminococcaceaeNK4A214group.id.11358*	-0.06 (-0.09, -0.03)	P<0.0001

β coefficients and 95% CI were calculated using linear regression model among non-diabetic participants in the Rotterdam Study, adjusted for age, sex, time in mail, Batch, alcohol intake, total energy intake, smoking status, physical activity, BMI, PPI, lipid-lowering medication, and education level, diet quality score, and blood pressure. All P values <0.0005 were set for statistical significance. Null associations were observed for other taxa and insulin resistance.

© 2021 Chen Z et al. *JAMA Network Open.*

eTable 6. Statistically Significant Associations Between Taxa and Type 2 Diabetes After Additionally Adjusting for Diet Quality and Blood Pressure

Taxa	T2D<br>OR (95% CI)	P values
family.Clostridiaceae1.id.1869	0.67 (0.59, 0.76)	P=0.0003
family.Peptostreptococcaceae.id.2042	0.74 (0.65, 0.83)	P<0.0001
genus.Clostridium sensu stricto1.id.1873	0.67 (0.58, 0.76)	P<0.0001
genus.Intestinibacter.id.11345	0.74 (0.65, 0.85)	P<0.0001
genus.Romboutsia.id.11347	0.77 (0.68, 0.88)	P<0.0001

OR and 95% CI were calculated using logistic regression model in the Rotterdam Study, adjusted for age, sex, time in mail, Batch, alcohol intake, total energy intake, smoking status, physical activity, BMI, PPI, lipid-lowering medication, and education level, diet quality score, and blood pressure. All P values <0.0005 were set for statistical significance. Null associations were observed for other taxa and T2D.

eMethods. Supplementary Methods

Collection of gut microbiome data

Details on microbiome data collection in the Rotterdam Study [1, 2] and the Lifelines-Deep Study [3, 4] are described elsewhere. Briefly, for the Rotterdam Study, participants were requested to collect a stool sample at their home using a Commode Specimen Collection System (Covidien, Mansfield, MA) and feces collection tube (Minigrip Nederland, Lelystad, The Netherlands) and to send it through regular mail to Erasmus MC. Upon arrival, samples were recorded and stored at -20°C. The time each sample was in the mail was recorded.[5], and adjusted for in our analyses. An automated stool DNA isolation kit (Diasorin, Saluggia, Italy) was used to isolate bacterial DNA. In the Rotterdam Study sample, a confounding effect driven by DNA isolation batches was observed and therefore adjusted for in analyses. The V3 and V4 hypervariable regions of the bacterial 16S rRNA gene were amplified and sequenced on the IIlumina MiSeq platform. For the Lifelines-Deep Study, the stool samples were picked up from participants' homes by students of University Medical Center Groningen. DNA was isolated with the AllPrep DNA/RNA Mini Kit (Qiagen; cat. #80204). The V4 hypervariable region of the bacterial 16S rRNA gene were amplified and

© 2021 Chen Z et al. *JAMA Network Open.*

sequenced on the Illumina MiSeq platform. To decrease domain-dependent bias related to different hypervariable regions between the two cohorts, a direct classification of 16S sequencing reads using a naive Bayesian classifier from the Ribosomal Database Project (RDP2.12), and SILVA 16S database release 128 was used to reconstruct taxonomic composition of studied communities, with binning posterior probability cutoff of 0.8 [5]. More specifically, all 16S libraries were rarefied to 10,000 reads prior to taxonomy binning. Furthermore, index sequences (12 bp) were removed from each read and concatenated to generate a unique index of 24 bp for each read-pair. Spacer and primer sequences were removed using TAGCleaner. Paired reads were merged using PEAR with the following settings: minimum overlap of 10 bp (default) and an average read quality phred-score of 20 over a 30 bp sliding window. Merged reads shorter than 200 bp were discarded. Reads were de-multiplexed using QIIME including extra quality filtering steps: merged reads were truncated before three consecutive low-quality bases; ambiguous bases were not allowed. Chimeric reads were removed using UCHIME. For each sample at average 5-6% of the total reads were removed [5]. We only analyzed taxonomical results using genus and higher taxonomic levels. All additional steps have been standardized across both cohorts, as aforementioned, including sub-sampling to 10,000 reads with fixed seed to allow for replicability, procedures of transformations, and the thresholds set for bacterial taxa to be included in the analysis (any taxon should be present in more than 10% of the cohort's samples). This filtering effectively reduced the total number of tests and also made cross-validation and meta-analysis among the two cohorts possible [3]. As a result, in the Rotterdam Study, the microbiome data contained information on 2 domains, 8 phyla, 15 classes, 18 orders, 33 families, and 126 genera. In the Lifelines-Deep Study, the microbiome data contained 2 domain, 12 phyla, 21 classes, 27 orders, 48 families, and 184 genera (Supplementary Figure 2). We also calculated a diversity (Shannon, richness, and Inverse Simpson indices), and β diversity (Bray-Curtis dissimilarity matrix) at genus level using the R package 'vegan', in both cohorts separately.

© 2021 Chen Z et al. *JAMA Network Open.*

Assessment of covariates

Information on education, smoking status, dietary intake, and physical activity were assessed through interviews and questionnaires. In the RS, physical activity was assessed with the LASA Physical Activity Questionnaire, activities were weighted by their intensity with Metabolic Equivalent of Task (MET)[6] and expressed in MET-hours per week. In the LLD, physical activity was assessed with the Short Questionnaire to assess Health-enhancing physical activity, and a physical activity composition score per week was calculated[7, 8]. To measure dietary intake, a 389-item food frequency questionnaire was used in the RS[9], a 125-item food questionnaire was used in the LLD, from which dietary risk factors (e.g., total energy intake, alcohol intake) were calculated[8]. Height and weight were measured in each study's research centers and body mass index ((kg/m^2)) was calculated. In both cohorts, information on medication use was obtained from general practitioners, pharmacies' databases, Nationwide Medical Registry, or follow-up examinations[8, 10].

eReferences

1. Radjabzadeh, D., A.G. Uitterlinden, and R. Kraaij, *Microbiome measurement: Possibilities and pitfalls.* Best Practice & Research Clinical Gastroenterology, 2017.
2. Radjabzadeh, D., et al., *Diversity, compositional and functional differences between gut microbiota of children and adults.* Scientific Reports, 2020. **10**(1): p. 1040.
3. Tigchelaar, E.F., et al., *Cohort profile: LifeLines DEEP, a prospective, general population cohort study in the northern Netherlands: study design and baseline characteristics.* BMJ open, 2015. **5**(8): p. e006772.
4. Kurilshikov, A., et al., *Gut Microbial Associations to Plasma Metabolites Linked to Cardiovascular Phenotypes and Risk: A Cross-Sectional Study.* Circulation research, 2019. **124**(12): p. 1808-1820.
5. Kurilshikov, A., et al., *Genetics of human gut microbiome composition.* bioRxiv, 2020: p. 2020.06.26.173724.
6. Chen, Z., et al., *Plant-based diet and adiposity over time in a middle-aged and elderly population: the Rotterdam Study.* Epidemiology, 2018.
7. Wendel-Vos, G.C.W., et al., *Reproducibility and relative validity of the short questionnaire to assess health-enhancing physical*

© 2021 Chen Z et al. *JAMA Network Open.*

activity. Journal of clinical epidemiology, 2003. **56**(12): p. 1163-1169.
8. Scholtens, S., et al., *Cohort Profile: LifeLines, a three-generation cohort study and biobank.* International journal of epidemiology, 2014. **44**(4): p. 1172-1180.
9. Chen, Z., et al., *Plant versus animal based diets and insulin resistance, prediabetes and type 2 diabetes: the Rotterdam Study.* European journal of epidemiology, 2018. **33**(9): p. 883-893.
10. Bos, D., et al., *Intracranial carotid artery atherosclerosis and the risk of stroke in whites: the Rotterdam Study.* JAMA Neurol, 2014. **71**(4): p. 405-11.

© 2021 Chen Z et al. *JAMA Network Open.*
